# High-Density Genetic Mapping Identifies New Major Loci for Tolerance to Low-Phosphorus Stress in Soybean

**DOI:** 10.3389/fpls.2016.00372

**Published:** 2016-03-30

**Authors:** Dan Zhang, Hongyan Li, Jinshe Wang, Hengyou Zhang, Zhenbin Hu, Shanshan Chu, Haiyan Lv, Deyue Yu

**Affiliations:** ^1^Collaborative Innovation Center of Henan Grain Crops, College of Agronomy, Henan Agricultural UniversityZhengzhou, China; ^2^Zhengzhou National Subcenter for Soybean Improvement/Key Laboratory of Oil Crops in Huanghuaihai Plains, Institute of Industrial Crops, Henan Academy of Agricultural SciencesZhengzhou, China; ^3^Department of Biological Sciences, University of North Carolina at CharlotteCharlotte, NC, USA; ^4^Agronomy Department, Kansas State UniversityManhattan, KS, USA; ^5^National Key Laboratory of Crop Genetics and Germplasm Enhancement, National Center for Soybean Improvement, Nanjing Agricultural UniversityNanjing, China

**Keywords:** soybean, SLAF-seq, high density genetic map, QTL mapping, phosphorus efficiency

## Abstract

Phosphorus (P) is essential for all living cells and organisms, and low-P stress represents a major constraint on plant growth and yield worldwide. Soybean is an important economical resource of protein and oil for human and animals, and soybean is also a high-P demand species that is sensitive to low-P stress, which is considered a major constraint on soybean production. However, P efficiency is an important complex quantitative trait involving multiple genes, and the mechanisms underlying soybean P efficiency are largely unknown. Here, we reported the construction of a high-density genetic map using a specific-locus amplified fragment sequencing (SLAF-seq) strategy in soybean. This map, spanning 3020.59 cM in length, contained 6159 markers on 20 chromosomes, with an average distance of 0.49 cM between adjacent markers. Based on this map, 20 loci, including eight novel loci, associated with P efficiency-related traits were identified across multiple years and treatments. The confidence intervals of almost all QTLs were refined significantly, and the accuracy of this map was evidenced by coincident detections of the previously identified P efficiency-related genes *GmACP1* and *GmPT1*. Notably, a highly significant novel QTL located on chromosome 4, *q4-2*, was identified across traits, years and treatments. Several candidate genes, such as a pectin methylesterase-encoding gene (*Glyma.04G214000*) and a protein kinase gene (*Glyma.13G161900*), with significantly differential expression upon low-P stress were considered as promising candidates involved in regulating soybean P efficiency. Markers that tightly associated with P efficiency could be used for marker-assisted selection in a soybean P efficient breeding program. Further, dissection of these QTLs will facilitate gene cloning underlying P efficiency in soybean, and increase our understanding of efficient use of P in enhancing crop yield.

## Introduction

Phosphorus (P) involves in all living cells and organisms through the synthesis of nucleic acids (e.g., DNA and RNA) and the cellular energy storage molecule adenine tri-phosphate (ATP) as well as all cellular phosphorylation events (Johnston et al., 2000; Khan et al., 2009). In crops, P is an essential macronutrient for optimum growth and production (Penuelas et al., 2013). However, because almost half of the world's agricultural lands are now known to be severely deficient in this element (Elser, 2012) due to its low mobility and high fixation in soils (Kochian, 2012), P has become a worldwide constraint for crop productivity. Additionally, the global reserve of rock phosphate is a non-renewable resource. Some recent studies concluded that viable mineral P reserves are expected to be depleted in the near future (Van and Steven, 2010; Elser, 2012). Furthermore, insufficient P content in soil becomes more severe in some developing countries because of rapidly expanding population growth and a lack of financial support to obtain P-containing fertilizers. To meet these present and future challenges of limited P resources, identification and dissection of P-efficient genes represent the important steps for the subsequent development of P-efficient crops, securing sustainable food supply and agriculture (Gaxiola et al., 2012).

Soybean [*Glycine max* (L.) Merrill] is one of the most important grain and oil crops, accounting for approximately 56% of global oilseed production (Wilson, 2008). Compared with nonlegumes, relatively large amounts of P are needed during soybean growth, especially during the podding period (Li et al., 2011). Low-P stress affects soybean growth in many aspects, such as decreasing soybean nodule development and nitrogen fixation, increasing flower/pod abscission, and impairing the overall growth and development of plants, consequently affecting yield and seed quality (Cassman et al., 1980; Olivera et al., 2004; Zhang et al., 2010, 2014). Therefore, low-P stress is considered an important limiting factor constraining soybean production and is also more problematic than other nutrient deficiencies, toxicities or diseases in soybean (Gowin, 1997).

In the past decade, a number of quantitative trait loci (QTLs) associated with soybean P efficiency have been identified (Li et al., 2005; Zhang et al., 2009, 2010; Liang et al., 2010), but few genes responsible for these QTLs have been cloned and applied in soybean breeding programs. To date, only one gene, *GmACP1*, underlying the QTL (*qPE8*) associated with P efficiency has been cloned using combined analyses of linkage and association mapping. The gene *GmACP1* underlying *qPE8* encodes an acid phosphatase that can increase P efficiency in soybean (Zhang et al., 2014). The main reason for the limited number of identified and cloned genes is that low coverage of markers may lead to failure to detect QTL or map QTL to large chromosomal regions (10–20 centimorgans) that often contain a great number of predicted genes.

A genetic linkage map provides the foundation for QTL mapping. In soybean, a number of genetic maps have been constructed using restriction fragment length polymorphism (RFLP) markers, simple sequence repeats (SSR) or a small amount of single nucleotide polymorphism (SNP) markers in previous studies (Keim et al., 1990; Gore et al., 2002; Hyten et al., 2010; Gutierrez-Gonzalez et al., 2011). Based on these genetic maps, more than 100 QTL associated with various traits have been identified (http://www.soybase.org). However, because of technology limitations, only a small number of molecular markers are used to construct genetic maps, which limits the efficiency and accuracy of QTL mapping.

High-density genetic maps can provide an essential framework for QTL fine mapping (Qi et al., 2014). Increasing marker density can significantly increase the resolution of a genetic map, thus enhancing the precision of QTL mapping (Yu et al., 2011). Next generation sequencing (NGS) technologies, such as restriction site-associated sequencing (RADseq) (Miller et al., 2007), whole-genome resequencing (WGRS) (Xie et al., 2010; Xu et al., 2013), and two-enzyme genotyping-by-sequencing (GBS) (Poland et al., 2012) enable researchers to quickly obtain a large number of SNPs throughout the genome for high-density genetic map construction. Recently, a specific-locus amplified fragment sequencing (SLAF-seq) technology has been developed (Sun et al., 2013) and used to create high-density genetic maps in various plant species, such as rice (Mao et al., 2015), soybean (Li et al., 2014; Qi et al., 2014), and cucumber (Xu et al., 2014), among others.

Soybean represents a model legume plant; identification of P efficiency-related genes in soybean will facilitate P efficient breeding in soybean and other legumes or plant species. In previous studies, a recombinant inbred line (RIL) population derived from a cross between varieties “Nannong 94-156” that possessed high-P efficiency and “Bogao” with low-P efficiency has been used to map QTL for P efficiency across years, traits and growth periods. However, most QTLs for P efficiency are generally localized to large chromosomal regions because of the use of low-density genetic map containing only approximately 300 SSR markers. In this study, to refine and discover novel QTL and explore P efficiency-related genes, we adopted a SLAF-seq technology in an attempt to construct a high-density genetic map using the same soybean RIL population. Based on the soybean reference genome and comparative transcriptomic analyses of parental lines under low-P conditions, several potential genes involved in tolerance to low-P stress underlying P-efficient QTLs have been identified.

## Materials and methods

### Plant material and DNA extraction

A segregating soybean population consisting of 152 F_8:12_ RILs derived from a cross between varieties “Nannong 94-156” that possessed high-P efficiency and “Bogao” with low-P efficiency, was used to construct the high-density genetic map and map QTL for P efficiency. In this study, we selected 146 RILs from this population for genotyping and mapping analyses because this population is representative of the diverse genetic variation in soybean P efficiency. Eight P efficiency-related traits were determined during soybean seedling stage in greenhouse as previously described (Zhang et al., 2009). These traits include plant height (PH), root day weight (RDW), shoot dry weight (SDW), total dry weight (TDW), phosphorus acquisition efficiency (PAE), phosphorus use efficiency (PUE), P concentration (PC) and acid phosphatase activity (APA) under different P conditions (high-P, soil available *P* > 20 mg kg^−1^, low-P, soil available *P* < 5 mg kg^−1^ and the ratio of low/high P were designated as +P, -P and −∕+P, respectively). Two independent trials at soybean seedling stage were carried out in 2005 and 2006, respectively. The experiment was in a randomized complete block design with a split-plot arrangement. The main plots were P treatment and subplots were 146 genotypes. There were three replicates and each replicate contained six plants.

Seedlings of RILs and their parents were planted in an experiment field of Henan Agriculture University in Zhengzhou, Henan Province, China, in 2014. Two-week old leaf tissue of the two parents and RIL individuals were collected. Genomic DNA was extracted by modified CTAB method (Allen et al., 2006). DNA was quantified with an ND-1000 spectrophotometer (NanoDrop, Wilmington, DE, USA) and by electrophoresis in 0.8% agarose gels with lambda DNA as a standard.

### SLAF library construction and sequencing

The whole RIL population was genotyped using an improved SLAF-seq strategy to generate genome-wide SNP markers as described by Sun et al. (2013). The soybean reference genome (*Glycine max*Wm82.a2.v1) was used to perform a SLAF pilot experiment, and the SLAF library was conducted in accordance using the predesigned scheme. Two restriction enzymes (EcoRI and MseI, New England Biolabs, NEB, USA) were used to digest the genomic DNA. Duplex tag-labeled sequencing adapters (PAGE-purified, Life Technologies, USA) were subsequently ligated to the adenine-tailed fragments using T4 DNA ligase. Polymerase chain reaction (PCR) was performed using diluted restriction-ligation DNA samples, dNTP, Taq DNA Polymerase (NEB) and MseI primer (PAGE-purified, Life Technologies). PCR products were then purified using Agencourt AMPure XP beads (Beckman Coulter, High Wycombe, UK). The purified samples were pooled and separated by 2% agarose gel electrophoresis. Fragments ranging from 364 to 414 base pairs (with indexes and adaptors) in size were excised and purified using a QIAquick gel extraction kit (Qiagen, Hilden, Germany). Gel-purified products were then diluted for pair-end sequencing (each end 125 bp) using an Illumina HiSeq 2500 system (Illumina, Inc; San Diego, CA, USA) according to the manufacturer's recommendations.

### Sequence data grouping and genotyping

The SLAF marker grouping and genotyping were performed using procedures described by Sun et al. (2013). Briefly, low-quality reads (quality score < 30e) were filtered out and all SLAF pair-end reads with clear index information were clustered based on sequence similarity as detected by BLAT (Kent, 2002). Sequences with over 95% identity were grouped in one SLAF locus as described by Sun et al. Alleles were defined in each SLAF using the minor allele frequency (MAF) evaluation. For soybean is a diploid species, one locus can only contain at most four SLAF tags, groups containing more than four tags were filtered out as repetitive SLAFs, and those with two, three, and four tags were identified as polymorphic SLAFs. Each SLAF locus were then detected between parents, and SLAFs with more than 3 SNPs were first filtered out. Alleles of each SLAF locus were then defined according to the average sequence depths of SLAF markers, which were greater than 20-fold in parents and greater than 4-fold in RIL individuals. Genotype scoring was then performed using a Bayesian approach to further ensure genotyping quality. First, a posteriori conditional probability was calculated using the coverage of each allele and the number of single nucleotide polymorphism. Then, the genotyping quality score translated from the probability was used to select qualified markers for subsequent analysis (Zhang et al., 2015). Low-quality markers for each marker and each individual were deleted during the dynamic process. When the average genotype quality scores of all SLAF markers reached the cutoff value, the process stopped. High-quality SLAF markers for the genetic mapping were filtered by the following criteria: (i) average sequence depths should be > 4-fold in each progeny and > 20-fold in the parents, (ii) markers with more than 10% missing data were filtered, and (iii) the chi-square test was performed to examine the segregation distortion. Markers with significant segregation distortion (*P* < 0.05) were initially excluded from the map construction and were then added later as accessory markers.

### Linkage map construction

After genotyping the 146 RILs, marker loci were partitioned primarily into chromosomes based on their locations on the soybean genome. The modified logarithm of odds (MLOD) scores between markers were calculated to further confirm the robustness of markers for each chromosome. Markers with MLOD scores < 5 were filtered prior to ordering. The LOD scores and recombinant frequencies were calculated by two-point analysis, which were applied to infer linkage phases. To ensure efficient construction, the HighMap strategy was utilized to order the SLAF markers and correct genotyping errors within chromosomes (Liu et al., 2014). As a result, a high-density genetic map including 20 chromosomes was constructed as described in detail by Sun et al. (2013). In addition, the collinearity of chromosomes with the soybean reference genome was examined by aligning the sequence of each SLAF marker with genome sequences of Williams 82 using the BLASTN program at the National Center for Biotechnology Information (NCBI) (http://www.ncbi.nlm.nih.gov/).

### QTL mapping and candidate genes prediction

The composite interval mapping (CIM) program of WinQTLCart version 2.5 (Wang et al., 2005) was used to detect additive QTL for traits related to P efficiency using the 146 RILs. In order to detect significant QTL, a critical LOD threshold was established for all traits by conducting a test of 1000 permutations at a 5% significance level (Churchill and Doerge, 1994), cofactors were taken into account and a window size of 10 cM around the test interval was chosen for CIM analysis. Epistatic QTLs were analyzed using multiple interval mapping (Kao et al., 1999) with the WinQTLCart version 2.5 (Wang et al., 2005). MIM forward search method was chosen to initial models and BIC-M1 was chosen as model selection criteria. The walk speed is 1 cM and window size is also 10 cM. For each QTL, the position corresponding to the maximum LOD and the part of the phenotypic variation it explained was estimated.

The predicted genes in the target QTL region were analyzed according to the annotation of the soybean reference genome (Wm82.a2.v1) in Phytozome v10.3 (http://phytozome.net). Functional predictions of genes were manually confirmed by protein blasting (http://www.ebi.ac.uk/Tools/sss/ncbiblast/). In addition, GO enrichment analysis of predicted genes was performed using the GO website with default setting (http://bioinfo.cau.edu.cn/agriGO/analysis.php) (Du et al., 2010).

## Results

### High-throughput SLAF sequencing and genotyping

In this study, the 146 RILs and their parents were genotyped using SLAF-seq technology. According to the results of the pilot experiment, EcoRI and MseI were used for SLAF librariy construction. The library comprised SLAF fragments ranging from 364 to 414 bp in length. In total, 37.75 Gb of raw data was generated from Illumina sequencing and SLAF library construction. A total of 188.77 M paired-end reads were obtained for both parents and 146 RILs, with an average of 1.29 M reads for each individual line. Reads with low quality were discarded during quality control processing. This dynamic process was repeated until the average genotype quality score of all reads reached the cut-off value of 30 (Q30, indicating a 0.01% likelihood of an error, and thus 99.9% confidence). As a result, 108.09 M qualified reads were used for subsequent analysis (Table 1).

**Table 1:** **SLAF-seq data summary for soybean RIL population**.

**Total reads**	
Number of reads	188,773,688
Reads in high-quality SLAFs	108,090,579
Reads in repeat SLAFs	426,072
Reads in low depth SLAFs	21,962,950
**HIGH-QUALITY SLAFs**	
Number of SLAFs	253,809
Average SLAF depth	425.87
Average depth in parents	20.78
Average depth in individuals	4.48
**POLYMORPHIC SLAFs**	
Number of polymorphic SLAFs	34,720
Average depth in parents	20.05
Average depth in individuals	4.45
Number of SNPs	48,916
SNP ratio per kb	7.04
**HIGH-QUALITY SLAF MARKERS**	
Number of high-quality SLAF markers	6178

In addition to using quality scores to ensure the genotyping quality, sequencing depth was also used to determine qualified markers. In addition, the sequence depth of parents had more impact on allele calling because the parental genotypes were the basis for genotyping each marker. Specifically, 9,116,218 reads were generated for 230,310 SLAFs from the paternal inbred line (Nannong94-156) with an average coverage of 20.69-fold for each SLAF. Accordingly, in the maternal line (Bogao), sequencing of 228,355 SLAFs produced 8,311,407 reads, and the average cover for each SLAF was 20.87-fold. For the analysis of the 146 RILs, 812,374 to 2,091,889 reads were generated for the development of 121,293 to 190,925 SLAF markers for each line; the marker coverage ranged from 2.93 to 7.86-fold, with an average of 4.48-fold (Table 1, Figure S1A).

After filtering the low-quality reads and low-depth SLAFs, a total of 253,809 high-quality SLAFs were identified, of which 34,551 were polymorphic with a polymorphism rate of 13.61% (Table 1, Figure S1B). SLAFs lacking parental information were then filtered out, the resultant 30,354 SLAFs were classified into eight segregation patterns (Figure S2). After further filtering the heterozygous parents, 6178 SLAF markers with over 50-fold sequence depth for parental lines and over 7-fold sequence depth for progeny were qualified to construct a linkage map for the RIL population.

### Genetic map construction and its characteristics

The 6178 high-quality markers were distributed into 20 chromosomes according to their physical locations on the soybean reference genome and the MLOD scores with other markers (at least one MLOD score > 5). In addition, all markers on the map demonstrated 95% integrity (on average), which is a key parameter to control map quality. As a result, a total of 6159 markers (genotype data are listed in Table S1) were used to construct the final linkage map. The information of all markers on the map is organized in Table S2, including marker IDs, genetic position (cM), chromosomes and physical location (bp) in the soybean genome. Following linkage analysis, coverage of the 6159 markers was 54.19-fold in the male parent, 54.02-fold in the female parent and 7.89-fold in each RIL line (on average).

The map spanned a total of 3020.59 cM, with an average interval of 0.49 cM between adjacent markers (Table 2, Figure S3). On average, each chromosome contained 308 markers that spanned an average length of 151.03 cM. The genetic length of 20 chromosomes ranged from 75.64 cM (Chr20) to 207.94 cM (Chr19). The chromosomes were numbered according to the chromosome numbers of the W82 reference genome. Among 20 chromosomes, Chr15 was the most saturated, containing 616 markers with an average marker density of 0.25 cM. In contrast, Chr01 contained the largest intervals of 1.29 cM between adjacent markers. Moreover, the longest chromosome, Chr19, harbored 743 markers, covering a length of 207.94 cM with only a 0.28 cM average inter-marker distance. However, the shortest chromosome, Chr17, contained only 85 markers spanning a length of 102.03 cM, with an average inter-marker distance of 1.20 cM.

**Table 2:** **Characteristics descriptions of constructed 20 chromosomes**.

**Chr. ID**	**Total marker**	**Total distance (cM)**	**Average distance (cM)**	**Max gap**	**Gap < 5 cM**
Chr01	96	123.64	1.29	12.48	95%
Chr02	333	175.36	0.53	10.26	98%
Chr03	457	182.1	0.40	14.28	99%
Chr04	197	141.25	0.72	9.87	99%
Chr05	111	117.73	1.06	7.62	97%
Chr06	245	163.85	0.67	6.84	99%
Chr07	403	176.17	0.44	10.73	99%
Chr08	160	151.35	0.95	8.07	98%
Chr09	241	109.32	0.45	16.13	98%
Chr10	446	151.66	0.34	9.39	98%
Chr11	86	109.43	1.27	11.1	98%
Chr12	251	172.02	0.69	9.2	97%
Chr13	372	162.53	0.44	11.76	99%
Chr14	496	200.84	0.40	12.26	98%
Chr15	616	153.41	0.25	4.56	100%
Chr16	295	176.92	0.60	6.92	100%
Chr17	85	102.03	1.20	8.07	95%
Chr18	438	167.4	0.38	18.27	100%
Chr19	743	207.94	0.28	13.57	98%
Chr20	88	75.64	0.86	3.34	100%
Max group	743	207.94	1.29	18.27	98%
Min group	85	75.64	0.25	3.34	95%
Total	6159	3020.59	0.49	/	/

### Evaluation and comparative analysis of the genetic map

To assess the quality of this genetic map, heat maps were generated to evaluate the genetic map quality by using pair-wise recombination values for the 6159 SLAF markers (Figure S4). These heat maps indicate that the construction of this map was accurate, as the recombination frequency was considerably low among adjacent markers.

Crossover events that occur in an advanced population usually can be reflected by drawing a haplotype map, which can also reflect genotyping errors. Here, haplotype maps for each of the 146 RILs and the parental controls using 6159 SLAF markers were generated (Figure S5) as described by West et al. (2006). As shown in Figure S3, most of the recombination blocks were clearly defined. Only less than 0.1% of these blocks were heterozygous, and less than 0.1% was missing. Moreover, markers allocated on each chromosome were evenly distributed with a mean interval of 0.49 cM between these markers. Thus, this RIL population with high recombination frequency was well purified and suitable for genetic analysis using the marker-density linkage maps.

**Figure 1:**
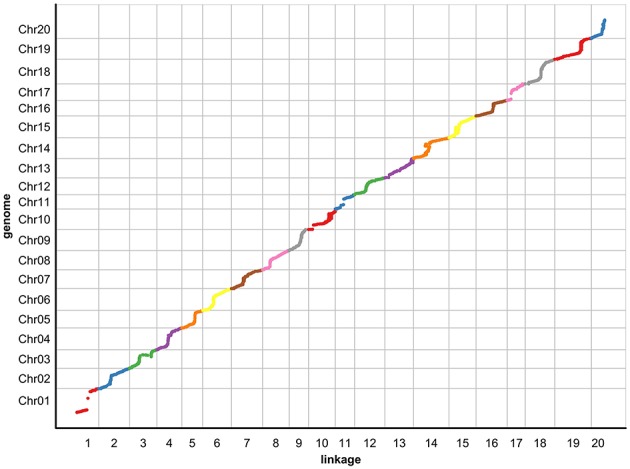
**The collinearity of 20 chromosomes with the soybean reference genome**. The x-axis indicates the genetic distance of soybean chromosomes accordingly, and the y-axis represents the linearity order of the physical position in the soybean genome. All 6159 SLAF markers in these chromosomes are plotted as dots on the Figure. Different colors indicate different chromosomes.

To evaluate the collinearity between the genetic map and the soybean reference genome, all SLAF markers were mapped to the soybean reference genome (Figure 1). As shown in Figure 1, consecutive curves between physical distances and genetic distances were observed in 20 chromosomes except Chr1 and Chr10. The high collinearity suggests that 6159 SLAF markers were accurately placed on 20 chromosomes, and the soybean genome was sufficiently covered with these SLAF markers. Most parts of these curves represent a rising trend, suggesting that their genetic and physical positions follow an identical order.

### Significant phenotypic variation under low-P stress among soybean RILs

To determine the genetic variation of P efficiency in soybean plants, eight P efficiency-related traits during soybean seedling stage were determined using 152 soybean recombination inbred lines (RILs) by Zhang et al. (2009, 2014). In the previous study, compared to the parent Nannong94-156, SDW, RDW, TDW, and PAE of the parent Bogao was higher in different P levels and trials, which was consistent with the fact that Bogao was a high biological-yielding soybean variety. However, PUE, PC, and APA of Nannong94-156 was higher than Bogao under different P levels and trials, which indicate that the former was a low-P stress tolerant variety. The transgressive segregation of P efficiency-related traits was obvious (Figure S6), and the phenotypic variation was significantly (*P* < 0.01 or *P* < 0.001) affected by the genotypes and treatments (Zhang et al., 2009, 2014). For example, the phenotypic RIL values ranged from 12.45 to 22.19 cm for PH, 1.34–3.84 mg g^−1^ for PC, and 0.85–2.94 μmol ρ-NP min^−1^ mg protein^−1^ for APA in the +P condition, while the range of phenotypic values under low-P stress were 10.90–19.81 cm for PH, 0.91–3.34 mg g^−1^ for PC, and 1.14–3.07 μmol ρ-NP min^−1^ mg protein^−1^ for APA, respectively. In addition, the skewness and kurtosis for the distributions of these traits in the RIL population were less than 1.0 in absolute (Figure S6), which indicated that all the eight traits approximately fitted normal distributions and the data was suitable for QTL mapping.

### QTL mapping and candidate genes prediction

**Figure 2:**
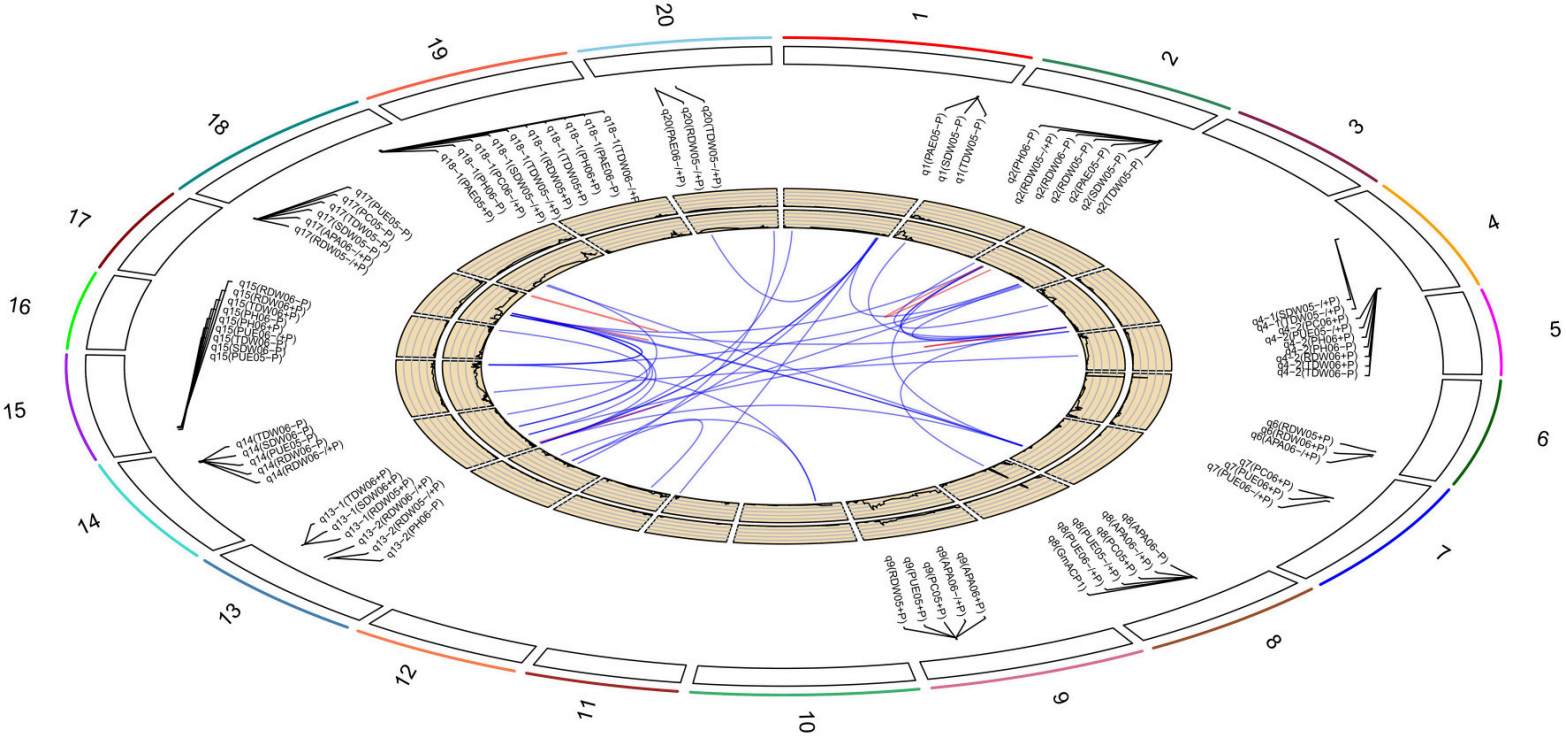
**Soybean chromosomes and main QTLs for investigated traits under high-P (+P), low-P (−P) conditions and the relative value (−∕+P)**. The lines link denotes epistatic associations between QTL and QTL. Blue line denotes two QTLs in different chromosomes, while red line denotes two QTLs in the same chromosome. The outside/inside wheat-colored circle indicates the LOD/*R*^2^ value curve for acid phosphatase activity at −∕+P in 2006, respectively. The outermost circle indicates the 20 soybean chromosomes, main QTLs for investigated traits under high-P (+P), low-P (−P) conditions and the relative value (−∕+P) and the position of these QTLs on the chromosomes.

**Figure 3:**
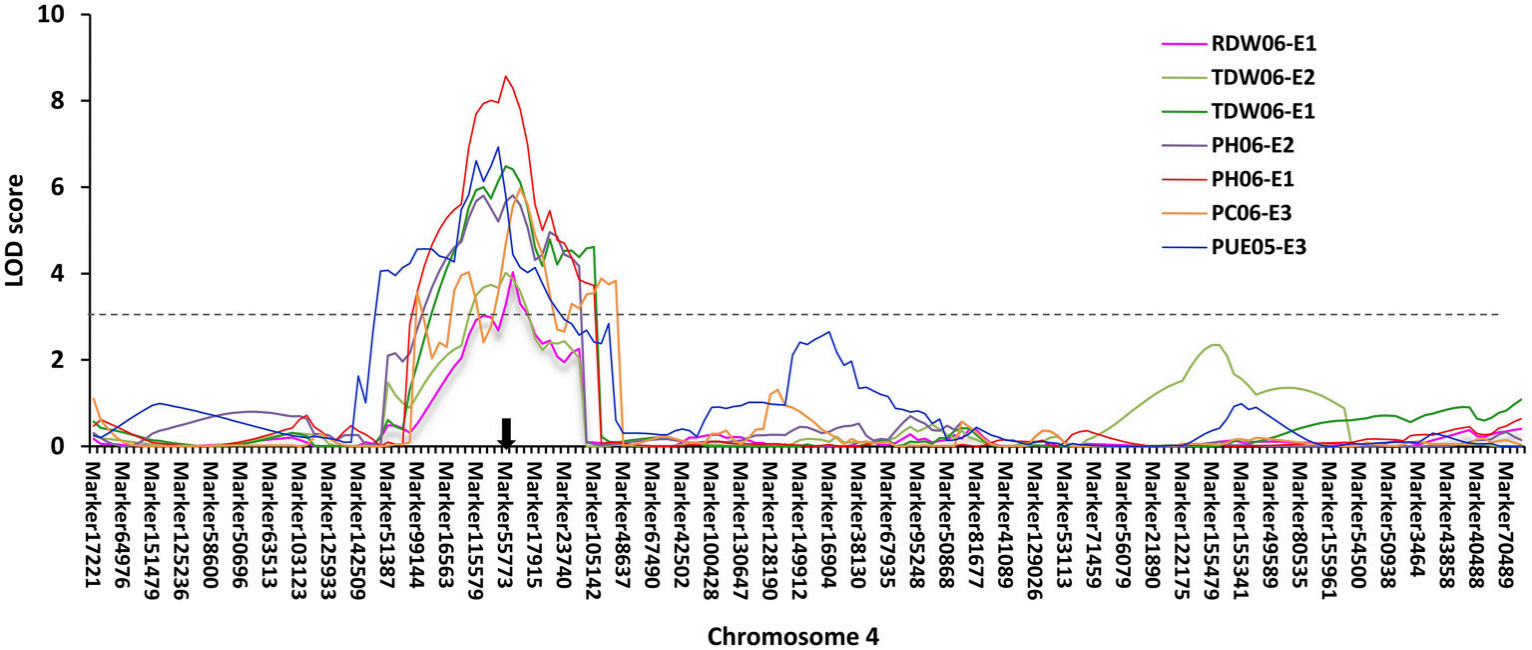
**P efficiency-related QTLs mapped on chromosome 4 using 146 RILs across years and treatments**. The black arrow denotes the P efficiency-related QTLs (*q4-2*) mapped to the location of *Glyma.04G214000* on chromosome 4. The x-axis scales genetic distance on soybean chromosome 4, while the y-axis represents the LOD scores. Different colors represent different traits across years and treatments (+P−∕+P).

Based on the high-density genetic map, a total of 85 QTLs underlying eight P efficiency-related traits (Zhang et al., 2009) were identified across years and treatments (Figure 2, Table S3). In this study, overlapped QTLs or adjacent QTLs with less than 5cM were classify into same loci. Base on this rule, 85 QTLs were classified into 20 genomic regions (loci), of which 12 loci (bold in Table 3) were previously identified using the same RIL population and confirmed in this study (Table 3). The remaining eight QTLs (*q1, q3, q4-1, q4-2, q7, q13-1, q13-2*, and *q16*) were novel. Notably, *q4-2* was associated with several P efficiency-related traits across years and treatments, including PH, PC, PUE, RDW, and TDW. The LOD score of this locus ranged from 3.36 to 8.57 for P efficiency-related traits, and these QTLs could explain 7.42–28.10% of phenotypic variance (Table 3). Therefore, the QTLs with high LOD and phenotypic variance explanation, such as *q4-2*, may be a novel important locus for P efficiency. As an example, the LOD curves of *q4-2* for P efficiency related traits are shown in Figure 3.

**Table 3:** **The characters of 20 consensus loci associated with P efficiency-related traits across various years and treatments**.

**Name^a^**	**Traits-years-treatments^b^**	**Chr.^c^**	**Marker interval^d^**	**Position^e^**	**LOD^f^**	***R*^2^^g^**
*q1*	SDW05-P, TDW05-P, PAE05-P	1	Marker338587–Marker397044	72.21–75.01	3.39	8.40
***q2***	**RDW05-P, SDW05-P, TDW05-P, PAE05-P, RDW05**−∕+**P, RDW06-P, PH06-P**	**2**	**Marker2373489–Marker2377055**	**19.19–20.81**	**3.71**	**8.63**
*q3*	SDW06-P, TDW06-P	3	Marker962011–Marker923093	58.11–59.27	3.57	7.37
*q4-1*	TDW05−∕+P, SDW05−∕+P	4	Marker41910–Marker131379	71.02–84.42	4.14	11.11
*q4-2*	PUE05−∕+P, RDW06+P, TDW06+P, TDW06-P, PH06+P, PH06-P, PC06−∕+P	4	Marker53079–Marker63513	15.21–19.56	5.14	12.59
***q6***	**RDW05+P, RDW06+P, APA06**−∕+**P**	**6**	**Marker2151148–Marker2105391**	**46.19–47.59**	**3.41**	**7.59**
*q7*	PC06+P, PUE06+P, PUE06−∕+P	7	Marker2525208–Marker2596654	89.41–94.55	3.89	9.38
***q8***	**PUE05**−∕+**P, PC05**−∕+**P, PUE06**−∕+**P, APA06-P, APA06**−∕+**P**	**8**	**Marker2634911–Marker2645532**	**85.14–88.42**	**4.51**	**11.95**
***q9***	**RDW05+P, PUE05+P, PC05+P, APA06+P, APA06**−∕+**P**	**9**	**Marker1603422–Marker1540438**	**47.53–49.68**	**3.20**	**7.21**
***q10***	**APA06-P**	**10**	**Marker641231–Marker703395**	**75.05–98.87**	**4.24**	**9.76**
*q13-1*	RDW05+P, SDW06+P, TDW06+P	13	Marker1786250–Marker1796581	27.92–38.03	3.84	9.20
*q13-2*	RDW05−∕+P, RDW06−∕+P, PH06-P	13	Marker1719580–Marker1684017	69.28–71.74	3.43	7.31
***q14***	**PUE05-P, RDW06-P, RDW06**−∕+**P, SDW06-P, TDW06-P**	**14**	**Marker473565–Marker461735**	**144.06–148.67**	**3.80**	**8.44**
***q15***	**PUE05-P, RDW06-P, RDW06+P, SDW06-P, TDW06+P, TDW06-P, PH06+P, PH06-P, PC06+P, PUE06**−∕+**P**	**15**	**Marker1381067–Marker1370961**	**50.34–51.40**	**4.89**	**12.17**
*q16*	PUE05+P, PC05+P	16	Marker1134679–Marker1148700	82.51–83.05	3.66	8.67
***q17***	**SDW05-P, RDW05**−∕+**P, TDW05-P, PUE05-P, PAE05**−∕+**P, PC05-P, PAE06**−∕+**P, APA06**−∕+**P**	**17**	**Marker2031528–Marker1984881**	**0–7.21**	**4.24**	**10.14**
***q18-1***	**PC06**−∕+**P, SDW05**−∕+**P, RDW05+P, TDW05+P, PH06+P, TDW05**−∕+**P, PAE05+P, TDW06**−∕+**P, PAE06-P, PH06-P**	**18**	**Marker2194216–Marker2328462**	**75.99–77.28**	**3.33**	**10.52**
***q18-2***	**RDW06-P, PH06-P**	**18**	**Marker2329091–Marker2270259**	**28.25–32.59**	**3.51**	**7.06**
***q19***	**RDW06-P**	**19**	**Marker1014646–Marker1106196**	**155.48–158.99**	**2.76**	**5.14**
***q20***	**RDW05**−∕+**P, TDW05**−∕+**P, PAE06**−∕+**P**	**20**	**Marker1445001–Marker1419806**	**9.54–15.52**	**3.24**	**7.53**

a *The name of the QTL is defined by the chromosome number*.

b *The traits-years-treatments of QTL is a composite of the influenced trait: root dry weight (RDW), shoot dry weight (SDW), total dry weight (TDW), phosphorus acquisition efficiency (PAE), phosphorus use efficiency (PUE), P concentration (PC), acid phosphatase activity (APA), and plant height (PH) followed by the years and treatments. +P denotes a QTL underlying the influenced trait at high-P condition, −P denotes a QTL at low-P condition and −∕+P denotes the ratio of the influenced trait under low/high P conditions*.

c *Chr indicates chromosome*.

d *Interval indicates confidence interval between two SLAF markers*.

e *Position indicates the interval of confidence in centimorgans*.

f *LOD indicates the average logarithm of odds score*.

g *R^2^ indicates the average phenotypic variance explained by related QTL*.

**Figure 4:**
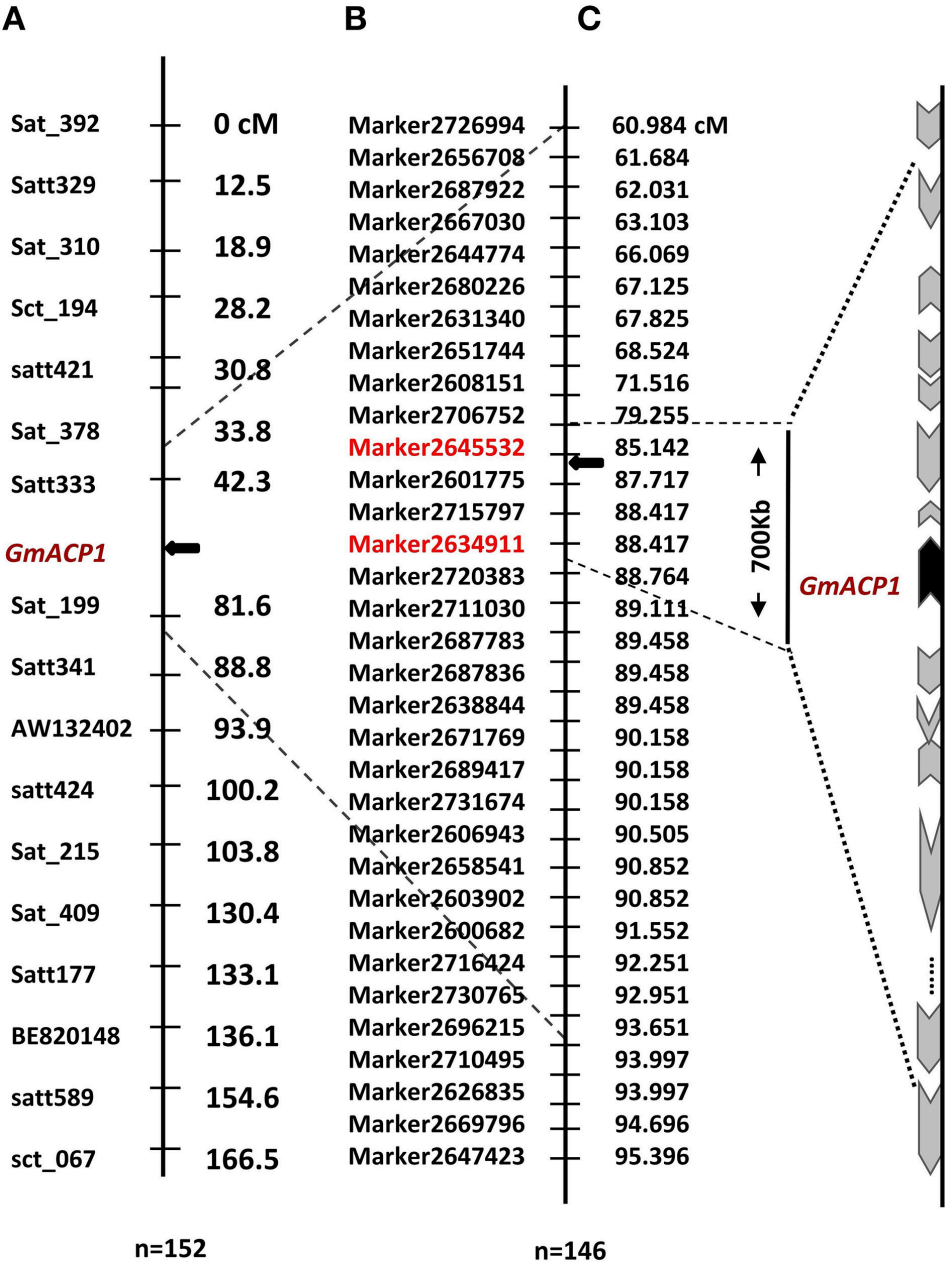
**Distribution of tightly linked markers and plausible P efficiency-related genes on chromosome 8 of soybean. (A)** A P efficiency-related QTL was mapped to the interval between markers Satt333 and Sat_199 on soybean chromosome 8. Left side of the chromosome denotes the marker names and right is the genetic distance (cM). **(B)** This QTL was further delimited to a 700 kb region on chromosome 8 using the present high-density genetic map and the same RIL population. Left side of the chromosome denotes marker names and the right is the genetic distance (cM). **(C)** The black arrow indicates the site of the predicted gene between Marker2634911 and Marker2645532 with lengths ranging from 85.1 to 88.4 cM. The black arrow denotes the position of an anchor gene (*GmACP1*), gray arrows denote the genes that is distributed on both sides of *GmACP1*. The length and direction of the gray arrows, representing gene length in percentage and transcription direction, respectively, were drawn according to the soybean reference genome.

By further analyzing these 20 loci, we found that eight loci could be detected more than 5 times across traits, years or treatments, and these eight loci were defined as the major QTL (Figure 2, Table 3). These QTLs perhaps represent the genetic basis of P efficiency and were thus focused on in subsequent analyses. As shown in Table 3, the eight QTLs (*q2, q4-2, q8, q9, q14, q15, q17*, and *q18-1*) were mapped on chromosomes 2, 4, 8, 9, 14, 15, 17, and 18. The average LOD score of these QTLs ranged from 3.20 to 4.89, and the average phenotypic variance explained by individual locus ranged from 7.21 to 12.17%. In addition, comparative analyses showed that six major QTLs identified in this study were co-localized with the previous identified QTLs (Zhang et al., 2009), and the P efficiency related genes, *GmACP1* and *GmPT1*, were localized to the correct genomic regions. For instance, a major QTL underlying acid phosphatase activity (Figure 2), P content and P use efficiency on chromosome 8 was stably identified across years and treatments, which corresponding to the *GmACP1* gene with LOD scores ranging from 3.02 to 6.31 and explaining 6.64–17.11% of the phenotypic variation. The co-localization of *GmACP1* with *q8* and the narrowed QTL regions provided strong evidence showing the high accuracy of the mapping strategy in the present study (Figure 4).

In addition, in this study, we not only detected more QTLs for P efficiency using the same RIL population based on the high-density genetic map, but the confidence intervals of these QTLs were also narrowed significantly (Table 3). For example, *q18-1*, a QTL underlying eight P efficiency related traits, was narrowed to a 0.71 cM interval compared to a previously reported 3.9 cM interval (Zhang et al., 2009). This 0.71 cM interval physically represents approximately 240Kb in reference genome, containing 58 putative predicted genes according to annotation of W82.a2.v1. Enriched GO categories revealed that seven predicted genes, encoding a phosphatase, protein kinase, protein phosphorylation and phosphotransferase, in this interval might be involved in the phosphate metabolic process (*P* < 0.01; Table S4). Another QTL, *q2*, that was previously mapped to an 18-cM interval (Zhang et al., 2009) was refined to a 0.16 Mb region in physical position. A comprehensive analysis of this region between Marker2373489 and Marker2377055 predicted 18 putative genes. Three of the 18 genes (*Glyma.02G266700, Glyma.02G267500*, and *Glyma.02G267900*) were considered candidates related to P efficiency or plant stress according to BLASTP querying in the protein database. Moreover, the novel major QTL, *q4-2*, were located in an approximate 0.36 Mb genomic region flanked by markers 53079 and 63513 on chromosome 4. This region contains 38 annotated genes. The gene *Glyma.04G214000* that encodes pectin methylesterase was regarded as a candidate for P efficiency, its expression was reported to be root- and stress specific which have reported can organ or stress-specific expression (Pelloux et al., 2007).

In addition to additive QTLs, we also analyzed the significant epistatic loci (*P* < 0.05) for the eight P efficiency-related traits across treatments using the multiple interval mapping (MIM) strategy. As a result, 41 pairs of QTLs, which had epistatic interactions with each other, were mapped on 12 chromosomes (Figure 2, Table S5). Among these epistatic loci, 11 pairs were identified for the eight P efficiency-related traits at +P condition, while 20 pairs of epistatic loci were identified at low-P condition, suggesting that low-P stress may induce the expression of epistatic gene underlying epistatic loci. In addition, 10 pairs epistatic loci associated with ratio of different traits at −P/+P were also identified, these loci might be related to P efficiency.

## Discussion

### Mapping population, density, and accuracy of the soybean genetic map

The selection of mapping populations is important for constructing a high-density map and further QTL analyses. In this study, we used the RIL population, derived from a cross between Nannong94-156 and Bogao, for constructing a high-density genetic map and subsequent QTL mapping of P efficiency. The major reasons using this population for further dissection of P efficiency QTL are the significant variations of many traits between the parental and RILs. In the previously studies, this population was used to construct a linkage map containing 248 SSR markers, which have mapped QTLs for P efficiency, biological yield, apparent harvest index and brachytic stem (Cui et al., 2007a,b, 2008). Using the same population, Zhang et al. (2009) made a denser linkage map with 306 markers to map QTLs underlying P efficiency (Zhang et al., 2009), and a fine map of flowering time QTL by combining linkage and association analysis (Zhang et al., 2013). Overall, the phenotypes investigated in this population exhibited significant variation in plant height, biological yield, number of main stems and 100-seed weight, and especially responses to low-P stress. Although a number of P efficiency QTLs have been identified previously (Zhang et al., 2009, 2010, 2014), few genes responsible for these QTLs have been cloned due to the low-density genetic linkage map. In this study, we significantly refined almost all QTLs and detected eight new QTLs related to P efficiency by using a high-resolution linkage map. Moreover, we can directly predict candidate genes within a narrow region between two adjacent markers based on the high-density genetic maps and the high-quality genome sequences. Accordingly, this strategy provided an efficient way for population genotyping, and the high-resolution linkage map could also be applied for QTL detection of other agronomic valuable traits. The narrowed QTLs also provided the several promising genes for further characterization.

It has been demonstrated that increasing marker density can improve the resolution of genetic maps and QTL in a given mapping population (Gutierrez-Gonzalez et al., 2011; Zou et al., 2012). However, the linkage disequilibrium (LD) of soybean is approximately 150 kb, which is relatively high compared to other crops (Lam et al., 2010), implying a limitation on the effectiveness of increasing marker density to improve the resolution of soybean genetic maps. Therefore, a suitable marker density in genetic maps could be theoretically saturated with 6300 evenly distributed markers. Recently, Hyten et al. (2010) has reported a soybean genetic linkage map, the Consensus Map 4.0, containing 5500 markers spanning a genomic map distance of 2296.4 cM, with a mean genetic distance of 0.6 cM between mapped SNP markers. In this study, by using SLAF-seq strategy, we constructed a linkage map containing 6157 SLAF markers and the average genetic distance between adjacent markers was only 0.49 cM. Moreover, compared to the Consensus Map 4.0 of soybean that was constructed by five populations using the GoldenGate assay SNP detection system (Illumina, USA), our genetic map was constructed based on a single RIL population, leading to efficient QTL mapping for a given trait. Furthermore, although some SLAF markers showed at the same position on this genetic map, the physical position was not actually at the same location (Table S2), which can be used for the QTL fine mapping and P efficiency-related genes prediction.

The gene prediction, however, should depend on a high accuracy and collinearity of this region between the genetic map and the soybean reference genome. We verified the quality and accuracy of this map from the QTL mapping studies in two particular cases where the underlying genes responsible for the QTL are known, *GmACP1* for P efficiency (Zhang et al., 2014) and *GmPT1* for phosphate transporter in soybean (Song et al., 2014). This accuracy was also indicated by the coincident location of six major QTLs identified here with their counterparts in previous studies, such as *q8* that was stably identified across traits, years and treatments. The co-location of *GmACP1* with *q8* provided strong evidence showing the high accuracy of the mapping strategy in the present study (Figure 4). Moreover, high collinearity between 20 chromosomes with the soybean reference genome enable us to analyze putative gene candidates associated with P efficiency within narrowed QTL containing a handful of predicted genes using the reference genome. Here, most of the eight major QTLs were mapped to intervals within 1.5 cM, and the narrowest region spanned only 0.16 Mb in physical position of the soybean genome (Table 3), containing only 18 predicted genes. In this regard, markers closely associated with P efficiency could be useful not only for marker-assisted selection (MAS) but also for further identification of P efficiency candidate genes through comparative mapping. Thus, this high-density genetic map exhibited higher efficiency and accuracy for P efficiency QTL mapping compared to the previous genetic map (Cui et al., 2007a; Zhang et al., 2009). Moreover, our study also demonstrated the usefulness of this high-density genetic map for dissection of P efficiency, a complex trait, which will be beneficial for studying other complex quantitative traits in this RIL population.

### Novel P efficiency QTL and candidate genes of interest

Of the 20 identified loci, eight QTLs (*q1, q3, q4-1, q4-2, q7, q13-1, q13-2*, and *q16*) were not found in previous reports, representing novel loci for P efficiency. It is noted that *q4-2* was associated with multiple P efficiency-related traits across various treatments. As shown in Table 3, Figure 2, the LOD score and phenotypic variation of this locus reached a maximum of 8.57 and 28.10%, respectively. Therefore, the loci were identified as an importantly novel major QTL associated with P efficiency. The release of the soybean reference genome has facilitated the gene prediction for QTL associated with various traits. In this study, a pectin methylesterase gene (*Glyma.04G214000*) in this 0.36 cM region was regarded as a candidate, as it was proposed to be required for plant tolerance to various abiotic stress (An et al., 2008), such as response to drought and oxidative stress (Legay et al., 2011), salt stress (Walia et al., 2005), heat stress (Kagan-Zur et al., 1995), and cold stress etc. (Lee and Lee, 2003). Moreover, previous studies have shown that the pectin methylesterase genes can have organ or stress-specific expression patterns (Pelloux et al., 2007). Similarly, our transcriptome analysis results showed that the expression of *Glyma.04G214000* increased 1000-fold in soybean leaves under low-P conditions relative to the control (Zhang et al., 2015, in review). Thus, *Glyma.04G214000* was putatively thought to be related to P efficiency and will be further experimentally verified.

The novel QTL, *q13-2*, were detected only under −P or −/+P conditions on chromosome 13, suggesting that this QTL's expression may be induced expression by low-P stress. A comprehensive analysis reveals that the target QTL was located in a region of approximately 1.5 Mb between Marker1719580 and Marker1684017, and 146 annotated genes were predicted. Six annotated genes (*Glyma.13G161800, 161900, 162800, 167600*, and *172100*) of 46 annotated genes within this QTL were considered candidates because of their relevance for P efficiency or plant stress. For example, one gene *Glyma.13G161900*, encodes a protein kinase with 86% of its amino acid sequence being identical to a rice protein kinase (*PSTOL1*). Overexpression of *PSTOL1* in rice significantly enhanced grain yield in phosphorus-deficient soil (Gamuyao et al., 2012). In addition, we also found another *PSTOL1*-like gene, *Glyma.13G280500* (74% identical to *PSTOL1*) within the region of the new QTL (*q13-1*), which is physically close to *q13-2* on chromosome 13. Although, the promising genes were provided within these loci, more studies such as characterizing the expression pattern of these genes are needed to uncover the genetic mechanism underlying P efficiency.

In summary, we reported the construction of a high-density genetic map in soybean, which demonstrates that the SLAF-seq strategy is an efficient method for marker discovery and high-density linkage map construction. Comparative analysis of QTL and fine mapping analyses suggest the high efficiency and accuracy of this genetic map. Using this high-density genetic map, we identified eight novel loci associated with P efficiency-related traits, and predicted several P efficiency-related candidate genes within extremely narrowed QTL intervals. One of eight loci was defined as a major QTL underlying multiple P efficiency-related traits across mulitple years and treatments. The high-phenotypic variation explained by this QTL demonstrated its importance for soybean P efficiency. In addition, several candidate genes, such as a pectin methylesterase-encoding gene (*Glyma.04G214000*) and a protein kinase gene (*Glyma.13G161900*) may be considered as promising candidates. Moreover, the markers closely linked to P efficiency will be potentially used for MAS in soybean P efficient breeding and facilitate the identification of genes underlying these loci for P efficiency. This study also illustrates the advantages of high-density genetic mapping analysis for deeper dissection of QTL underlying a complex trait.

## Author contributions

DZ, HL conceived and designed the experiments. DZ, HL, HZ, and SC performed the experiments, including DNA extraction, library preparation. DZ, JW, and HL performed SNP data analyses and linkage map construction. DZ and HZ wrote the manuscript. All authors read and approved the final version of the manuscript to be published.

### Conflict of interest statement

The authors declare that the research was conducted in the absence of any commercial or financial relationships that could be construed as a potential conflict of interest. The reviewer TV and handling Editor declared their shared affiliation, and the handling Editor states that the process nevertheless met the standards of a fair and objective review.

## Supplementary material

Figure S1 **Average sequencing depth and number of markers for each recombinant inbred line (RIL). (A)** Average sequencing depth of SLAF markers. **(B)** Number of SLAF markers. The x-axes indicate the individual RILs. The y-axes indicate the average depth and the number of markers.

Figure S2 **Number of each marker segregation pattern on the linkage map of soybean**.

Figure S3 **Soybean high-density genetic map**. The map was constructed based 6159 SLAF markers and markers position in Table S2. The SLAF markers and their location are shown on the right and left, respectively.

Figure S4 **Heat map of the high-density genetic map**. Each cell represents the recombination rate of two markers. Yellow indicates a lower recombination rate and purple a higher one.

Figure S5 **Haplotype map of the genetic map**. Blue represents Nannong94-156, red represents Bogao, white means the parent could not be estimated, gray represents deletions.

Figure S6 **Frequency distribution of P efficiency-related traits at high P (+P), low P (−P) and the ratio of low/high P (−∕+P)**. The red/green arrows denote the values of the two parents (Bogao and Nannong94-156) on the charts. SDW, shoot dry weight; RDW, root dry weight; TDW, total dry weight; PAE, P acquisition efficiency; PUE, P use efficiency; PH, plant height; APA, acid phosphatase activity; PC, P concentration; +P, high-P; −P, low-P; −∕+P, the ratio of the traits under low P to high P.

Table S1 **List of genotype data of 6159 SLAF markers**.

Table S2 **The SLAF marker names, genetic position, chromosomes and physical location in soybean genome**.

Table S3 **The characters of 85 QTLs associated with P efficiency-related traits**.

Table S4 **Several predicted candidate genes associated with P efficiency in QTL region**.

Table S5 **Epistatic loci for investigated traits under high-P, low-P, and the ratio of high/low-P conditions**.
