# Supplementary figures and images for: High-Density Genetic Mapping Identifies New Major Loci for Tolerance to Low-Phosphorus Stress in Soybean
Source: Front Plant Sci. 2016 Mar 30;7:372. doi: 10.3389/fpls.2016.00372 (PMC4811872; doi:10.3389/fpls.2016.00372)

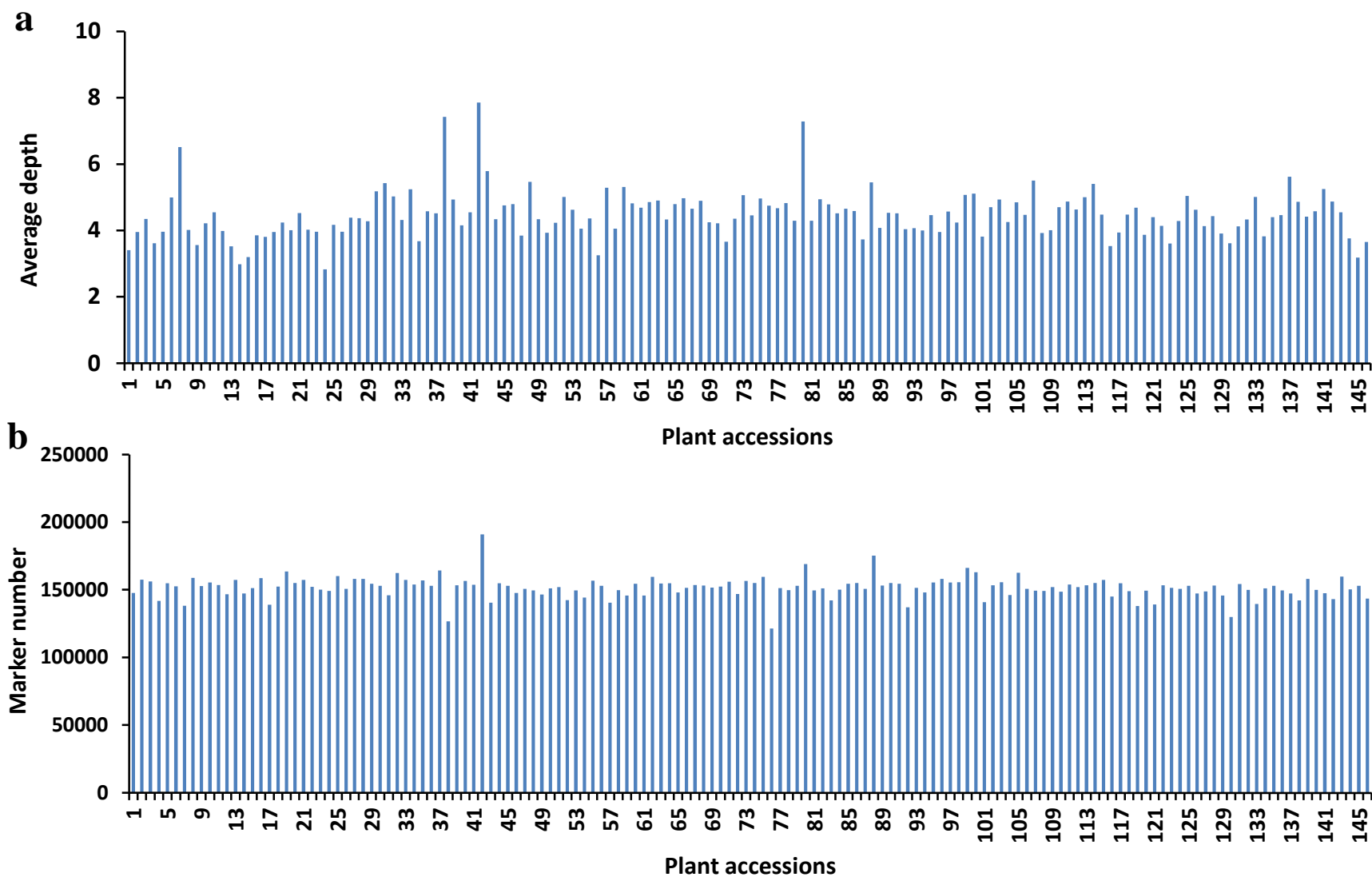

**Figure S1**

Supplement: Figure S1 — Average sequencing depth and number of markers for each recombinant inbred line (RIL). (A) Average sequencing depth of SLAF markers. (B) Number of SLAF markers. The x-axes indicate the individual RILs. The y-axes indicate the average depth and the number of markers. [file FigureS1.PDF]

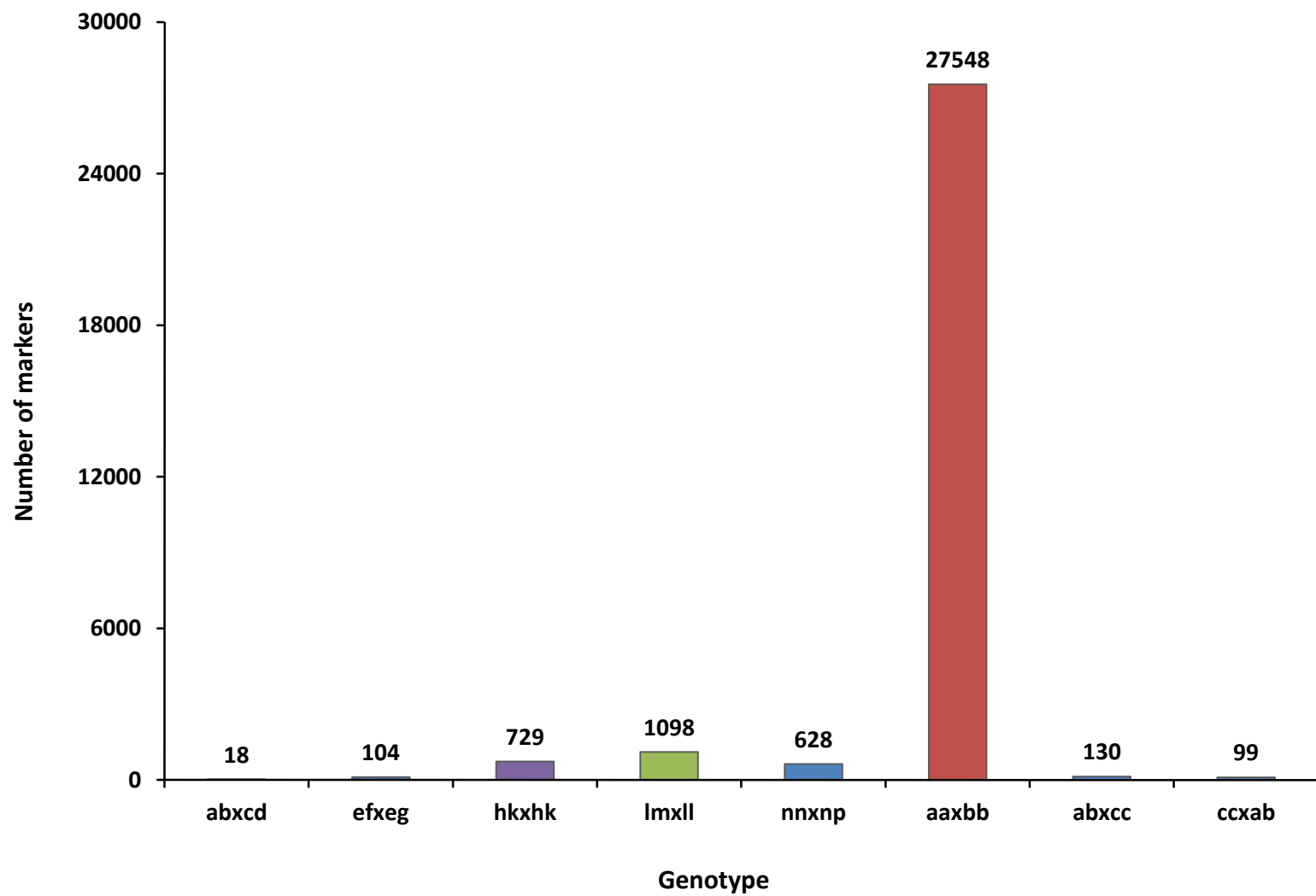

**Figure S2**

Supplement: Figure S2 — Number of each marker segregation pattern on the linkage map of soybean. [file FigureS2.PDF]

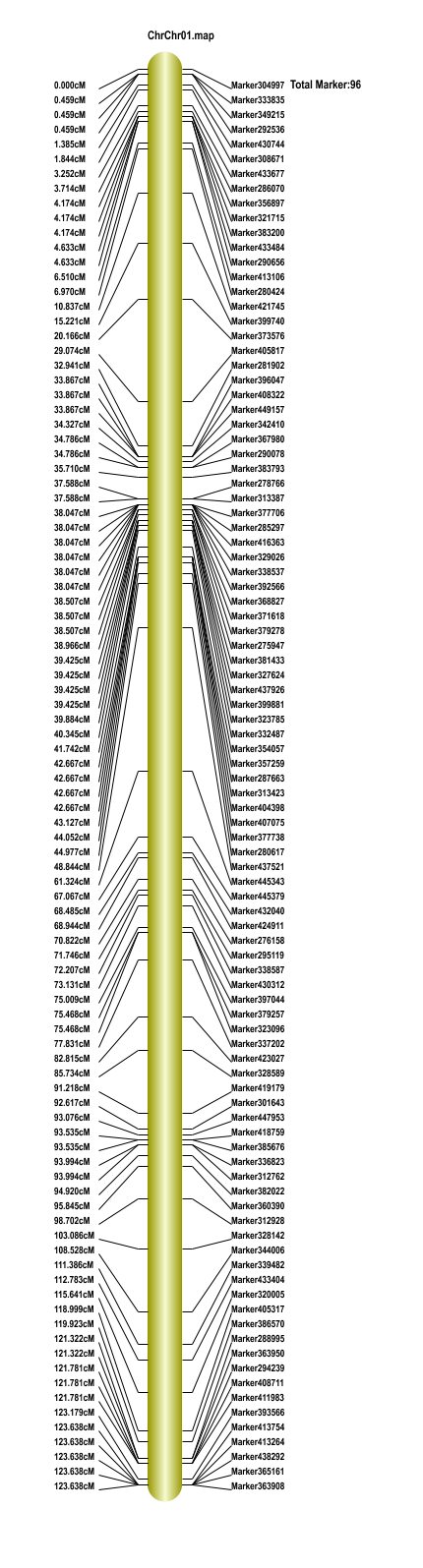

Supplement: Figure S3 — Soybean high-density genetic map. The map was constructed based 6159 SLAF markers and markers position in Table S2. The SLAF markers and their location are shown on the right and left, respectively. [file FigureS3.ZIP › soybeanChr01.map.png]

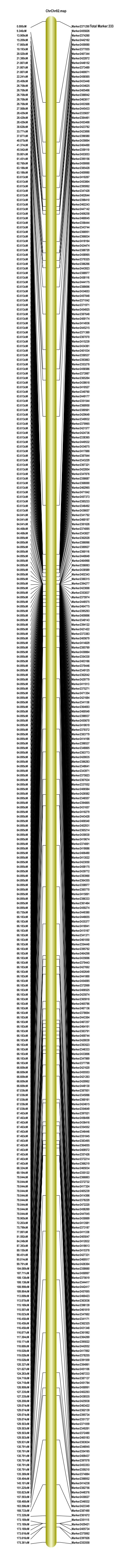

Supplement: Figure S3 — Soybean high-density genetic map. The map was constructed based 6159 SLAF markers and markers position in Table S2. The SLAF markers and their location are shown on the right and left, respectively. [file FigureS3.ZIP › soybeanChr02.map.png]

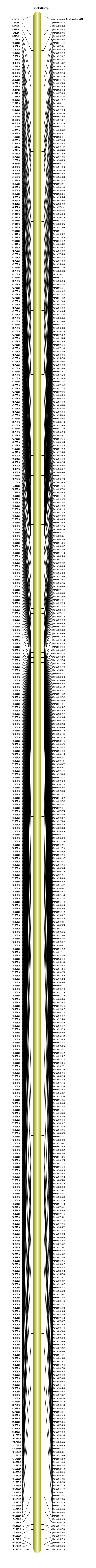

Supplement: Figure S3 — Soybean high-density genetic map. The map was constructed based 6159 SLAF markers and markers position in Table S2. The SLAF markers and their location are shown on the right and left, respectively. [file FigureS3.ZIP › soybeanChr03.map.png]

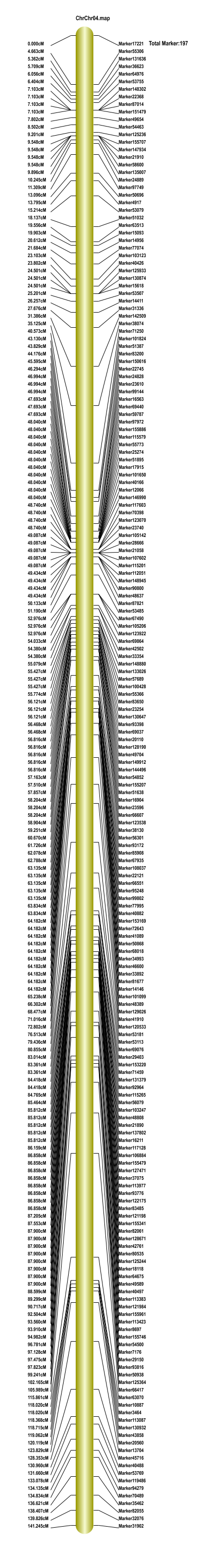

Supplement: Figure S3 — Soybean high-density genetic map. The map was constructed based 6159 SLAF markers and markers position in Table S2. The SLAF markers and their location are shown on the right and left, respectively. [file FigureS3.ZIP › soybeanChr04.map.png]

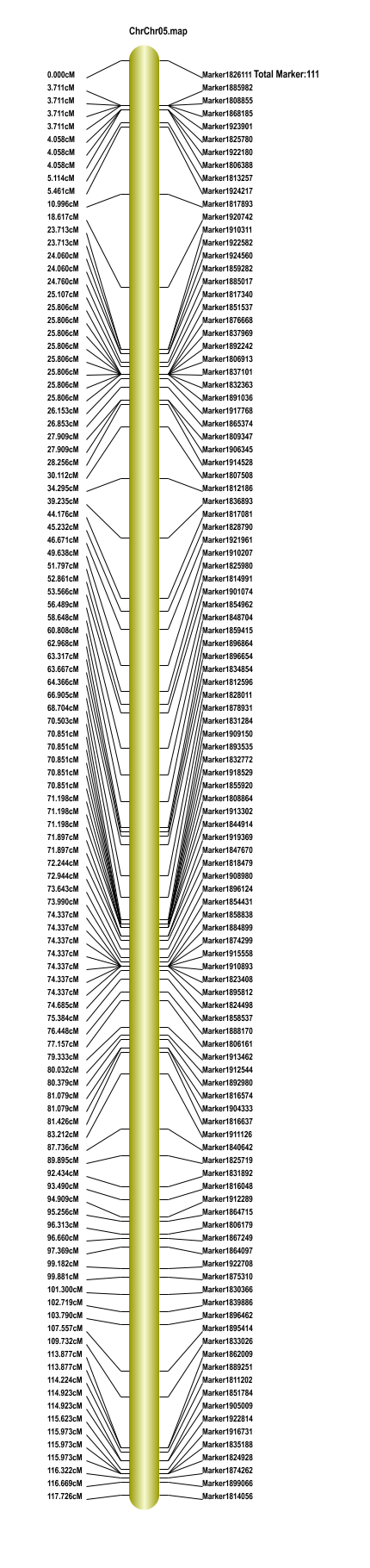

Supplement: Figure S3 — Soybean high-density genetic map. The map was constructed based 6159 SLAF markers and markers position in Table S2. The SLAF markers and their location are shown on the right and left, respectively. [file FigureS3.ZIP › soybeanChr05.map.png]

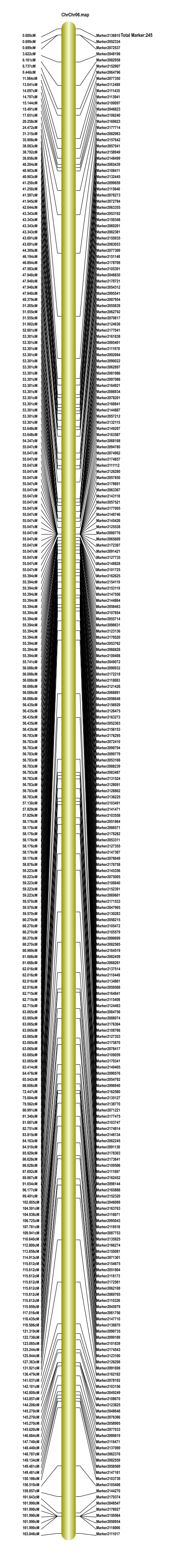

Supplement: Figure S3 — Soybean high-density genetic map. The map was constructed based 6159 SLAF markers and markers position in Table S2. The SLAF markers and their location are shown on the right and left, respectively. [file FigureS3.ZIP › soybeanChr06.map.png]

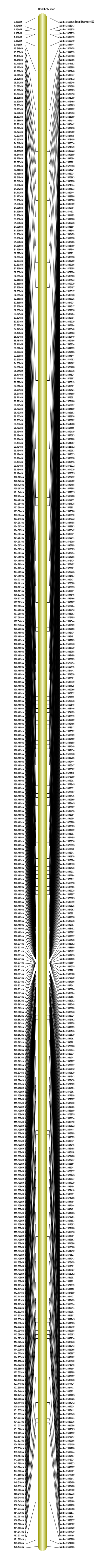

Supplement: Figure S3 — Soybean high-density genetic map. The map was constructed based 6159 SLAF markers and markers position in Table S2. The SLAF markers and their location are shown on the right and left, respectively. [file FigureS3.ZIP › soybeanChr07.map.png]

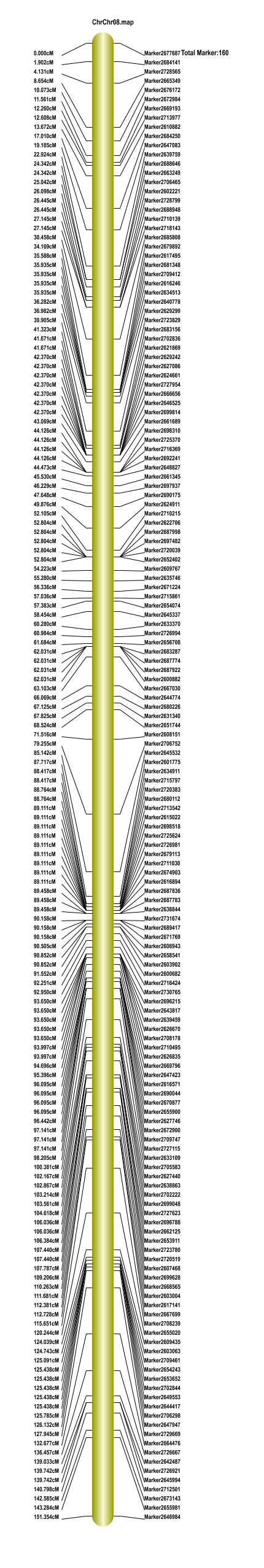

Supplement: Figure S3 — Soybean high-density genetic map. The map was constructed based 6159 SLAF markers and markers position in Table S2. The SLAF markers and their location are shown on the right and left, respectively. [file FigureS3.ZIP › soybeanChr08.map.png]

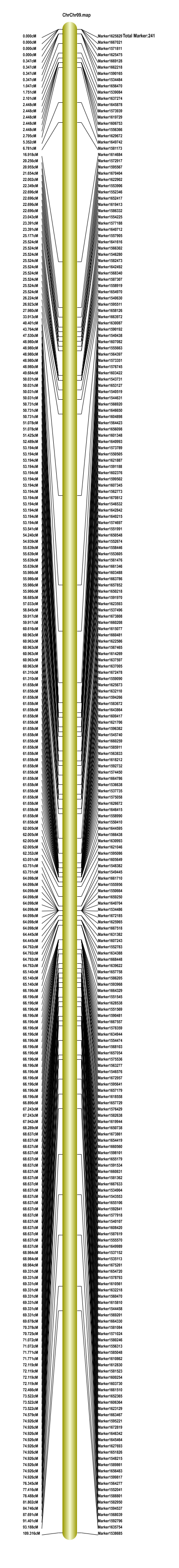

Supplement: Figure S3 — Soybean high-density genetic map. The map was constructed based 6159 SLAF markers and markers position in Table S2. The SLAF markers and their location are shown on the right and left, respectively. [file FigureS3.ZIP › soybeanChr09.map.png]

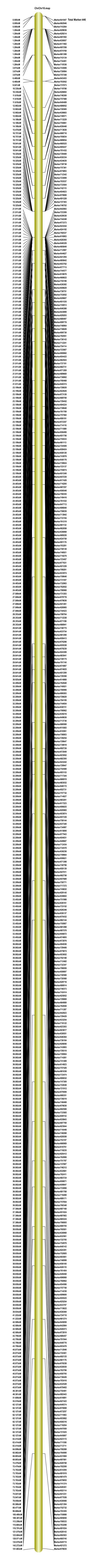

Supplement: Figure S3 — Soybean high-density genetic map. The map was constructed based 6159 SLAF markers and markers position in Table S2. The SLAF markers and their location are shown on the right and left, respectively. [file FigureS3.ZIP › soybeanChr10.map.png]

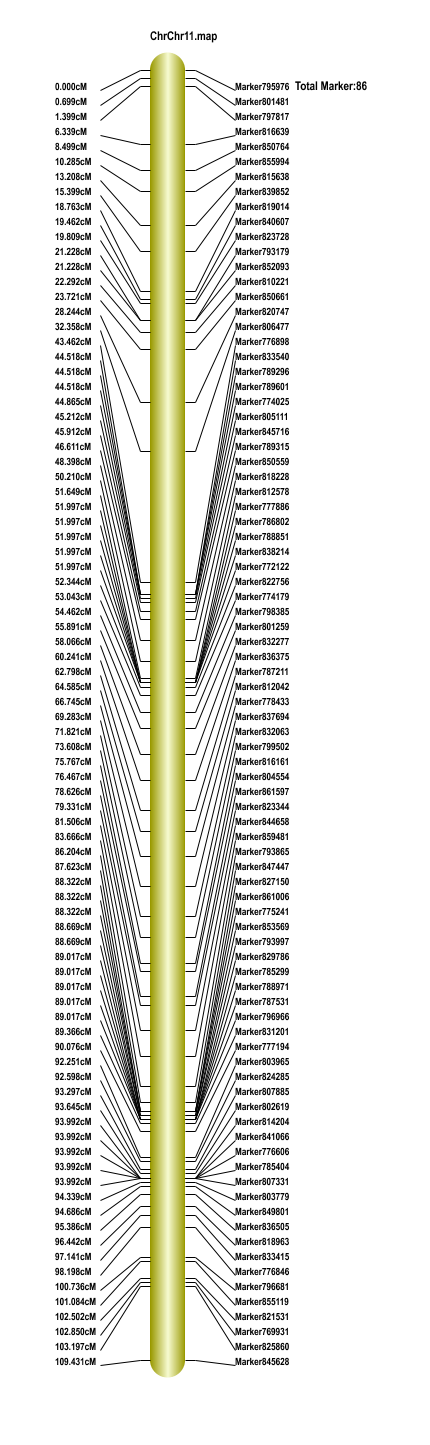

Supplement: Figure S3 — Soybean high-density genetic map. The map was constructed based 6159 SLAF markers and markers position in Table S2. The SLAF markers and their location are shown on the right and left, respectively. [file FigureS3.ZIP › soybeanChr11.map.png]

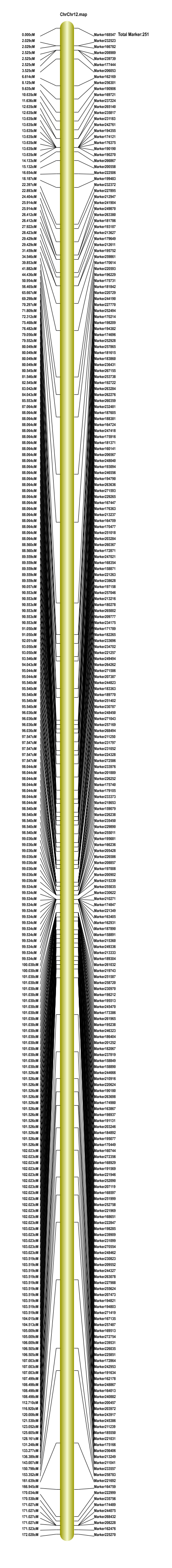

Supplement: Figure S3 — Soybean high-density genetic map. The map was constructed based 6159 SLAF markers and markers position in Table S2. The SLAF markers and their location are shown on the right and left, respectively. [file FigureS3.ZIP › soybeanChr12.map.png]

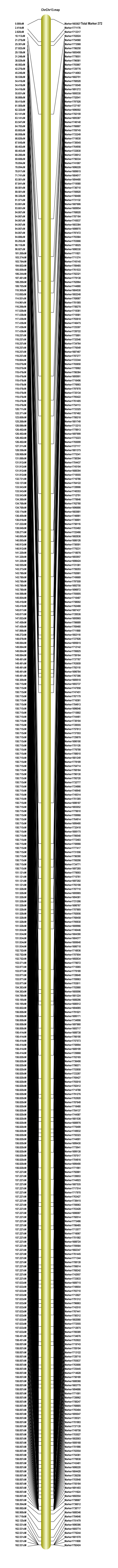

Supplement: Figure S3 — Soybean high-density genetic map. The map was constructed based 6159 SLAF markers and markers position in Table S2. The SLAF markers and their location are shown on the right and left, respectively. [file FigureS3.ZIP › soybeanChr13.map.png]

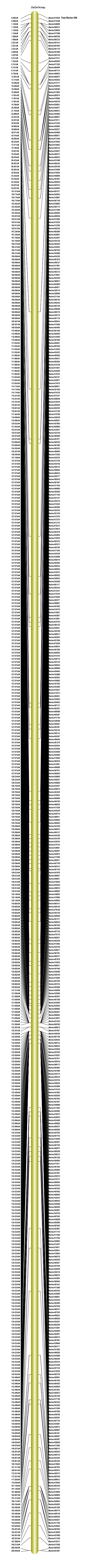

Supplement: Figure S3 — Soybean high-density genetic map. The map was constructed based 6159 SLAF markers and markers position in Table S2. The SLAF markers and their location are shown on the right and left, respectively. [file FigureS3.ZIP › soybeanChr14.map.png]

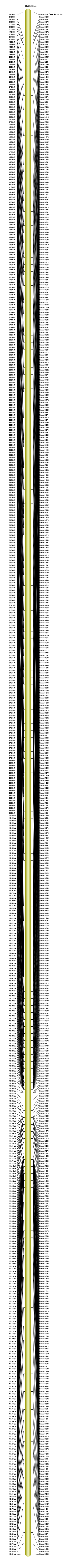

Supplement: Figure S3 — Soybean high-density genetic map. The map was constructed based 6159 SLAF markers and markers position in Table S2. The SLAF markers and their location are shown on the right and left, respectively. [file FigureS3.ZIP › soybeanChr15.map.png]

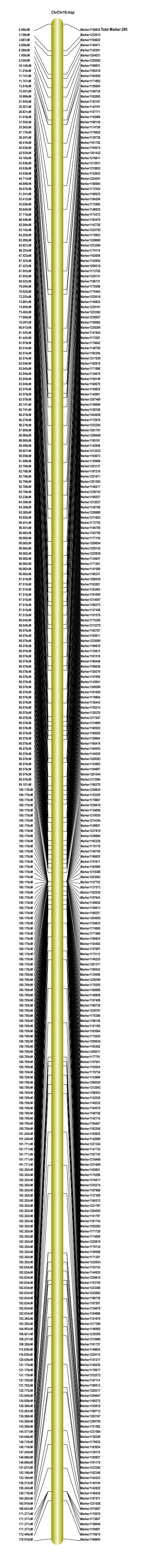

Supplement: Figure S3 — Soybean high-density genetic map. The map was constructed based 6159 SLAF markers and markers position in Table S2. The SLAF markers and their location are shown on the right and left, respectively. [file FigureS3.ZIP › soybeanChr16.map.png]

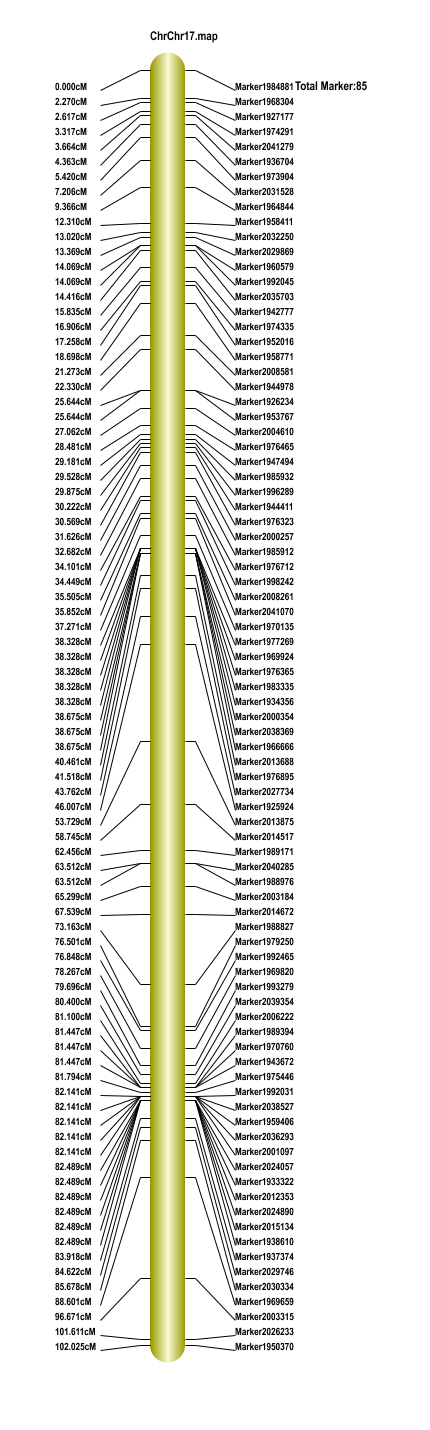

Supplement: Figure S3 — Soybean high-density genetic map. The map was constructed based 6159 SLAF markers and markers position in Table S2. The SLAF markers and their location are shown on the right and left, respectively. [file FigureS3.ZIP › soybeanChr17.map.png]

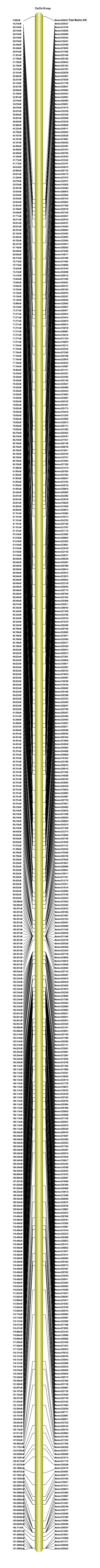

Supplement: Figure S3 — Soybean high-density genetic map. The map was constructed based 6159 SLAF markers and markers position in Table S2. The SLAF markers and their location are shown on the right and left, respectively. [file FigureS3.ZIP › soybeanChr18.map.png]

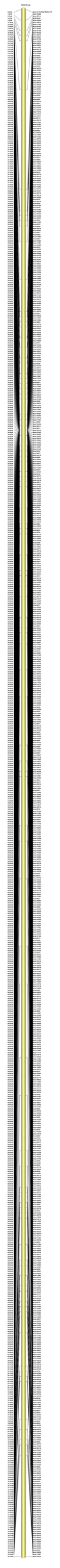

Supplement: Figure S3 — Soybean high-density genetic map. The map was constructed based 6159 SLAF markers and markers position in Table S2. The SLAF markers and their location are shown on the right and left, respectively. [file FigureS3.ZIP › soybeanChr19.map.png]

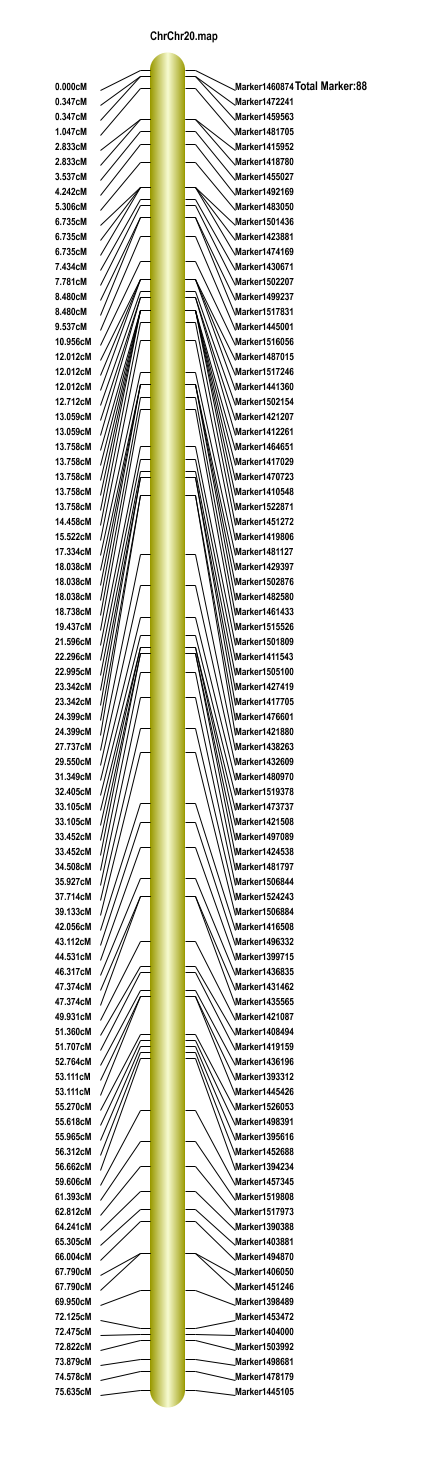

Supplement: Figure S3 — Soybean high-density genetic map. The map was constructed based 6159 SLAF markers and markers position in Table S2. The SLAF markers and their location are shown on the right and left, respectively. [file FigureS3.ZIP › soybeanChr20.map.png]

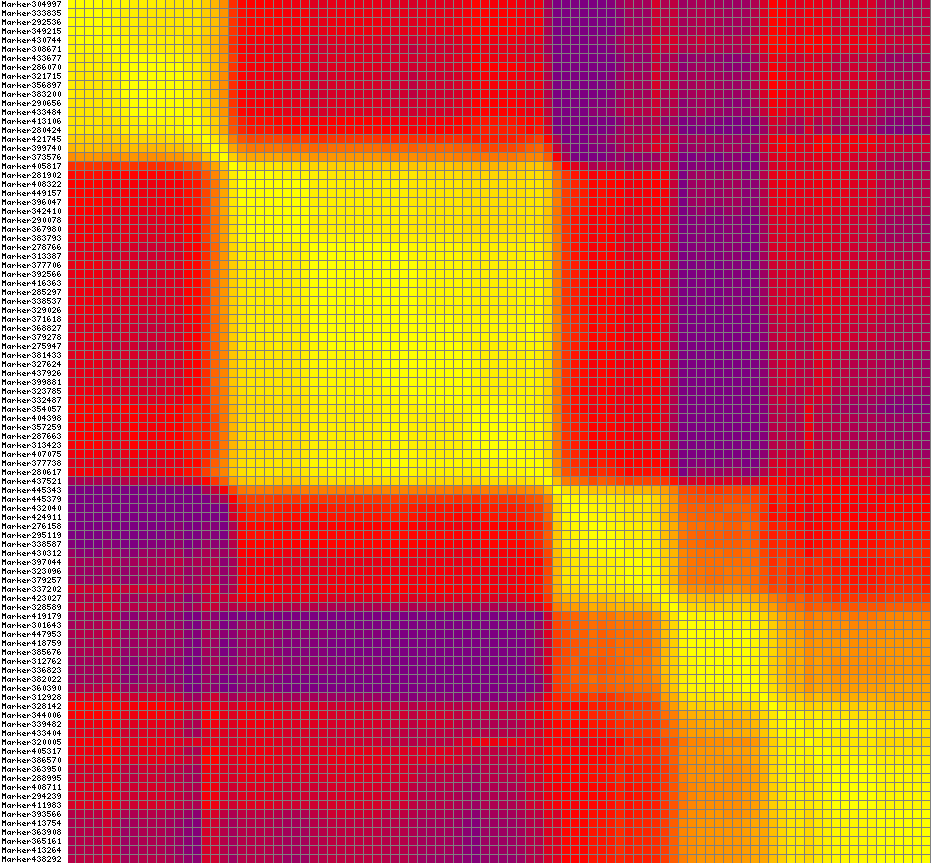

Supplement: Figure S4 — Heat map of the high-density genetic map. Each cell represents the recombination rate of two markers. Yellow indicates a lower recombination rate and purple a higher one. [file FigureS4.ZIP › Chr01.heatMap.png]

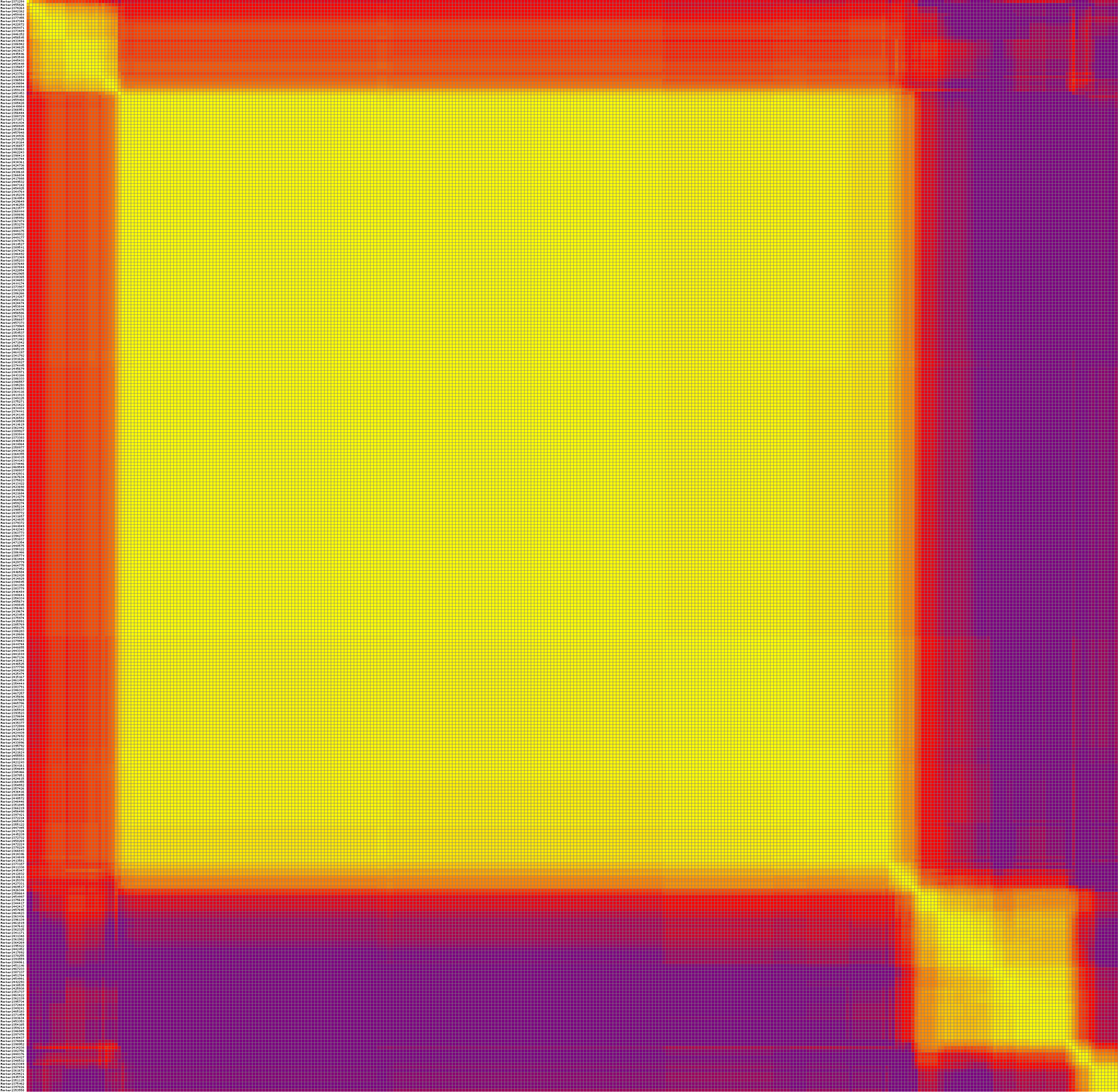

Supplement: Figure S4 — Heat map of the high-density genetic map. Each cell represents the recombination rate of two markers. Yellow indicates a lower recombination rate and purple a higher one. [file FigureS4.ZIP › Chr02.heatMap.png]

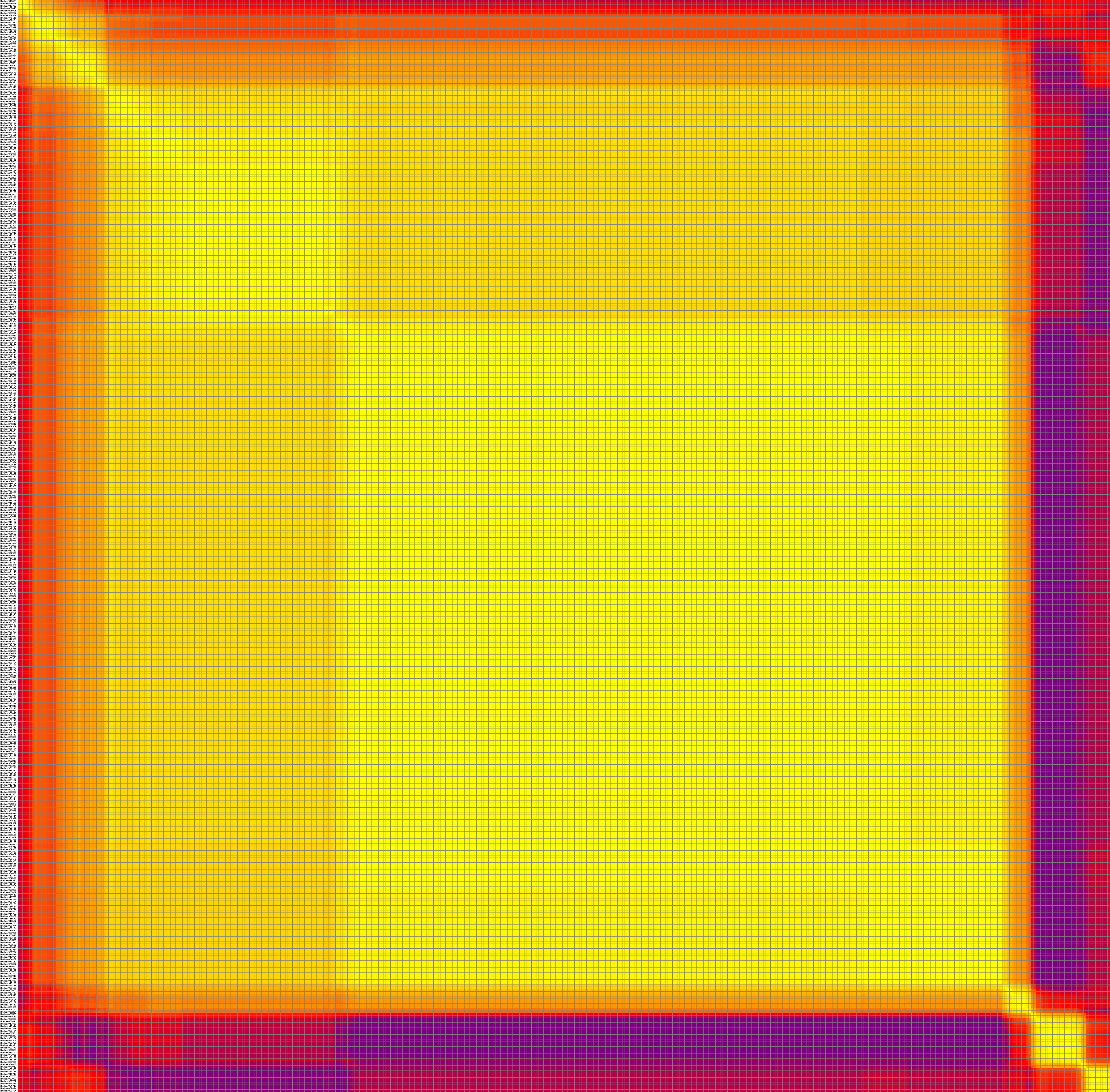

Supplement: Figure S4 — Heat map of the high-density genetic map. Each cell represents the recombination rate of two markers. Yellow indicates a lower recombination rate and purple a higher one. [file FigureS4.ZIP › Chr03.heatMap.png]

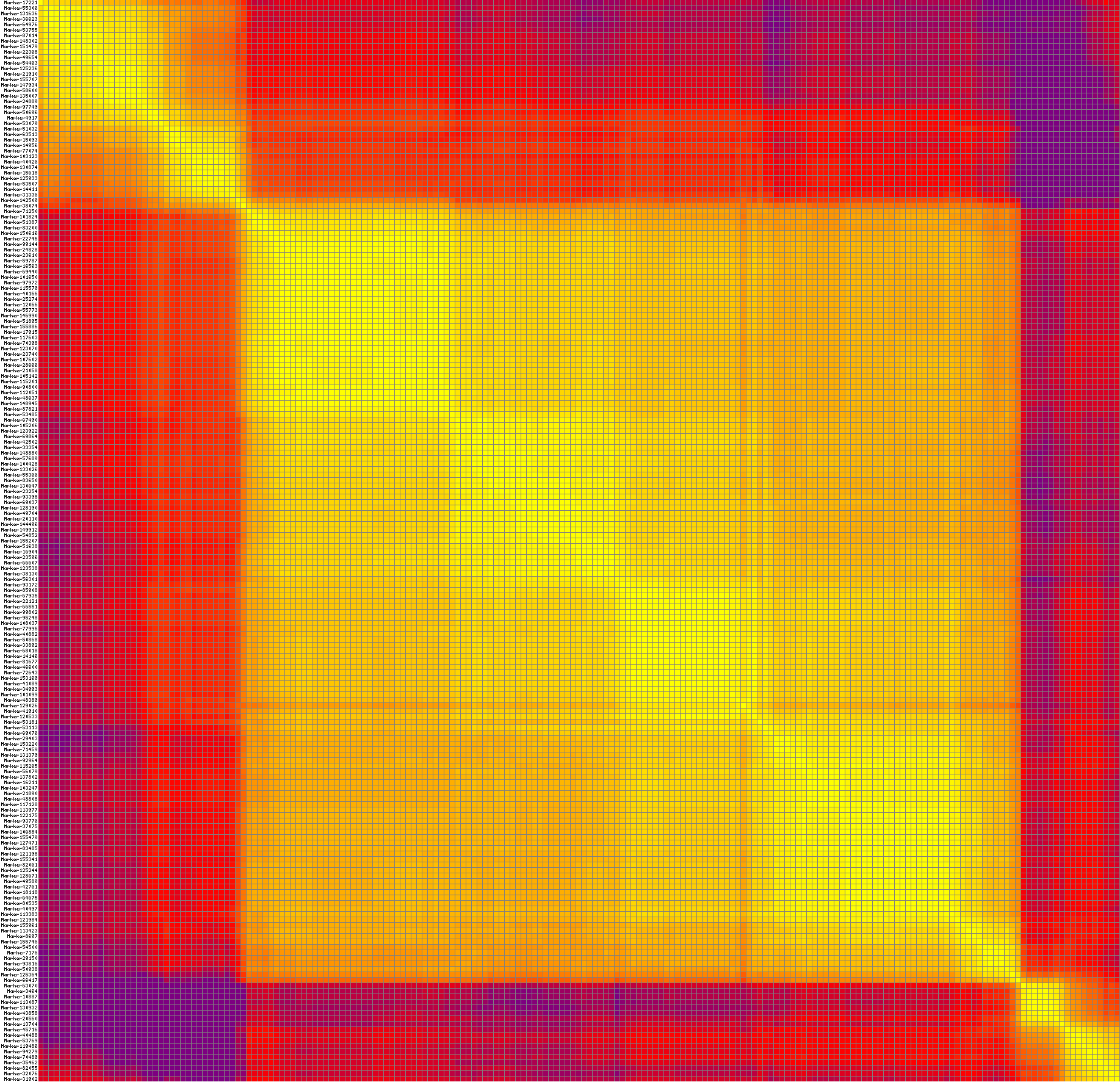

Supplement: Figure S4 — Heat map of the high-density genetic map. Each cell represents the recombination rate of two markers. Yellow indicates a lower recombination rate and purple a higher one. [file FigureS4.ZIP › Chr04.heatMap.png]

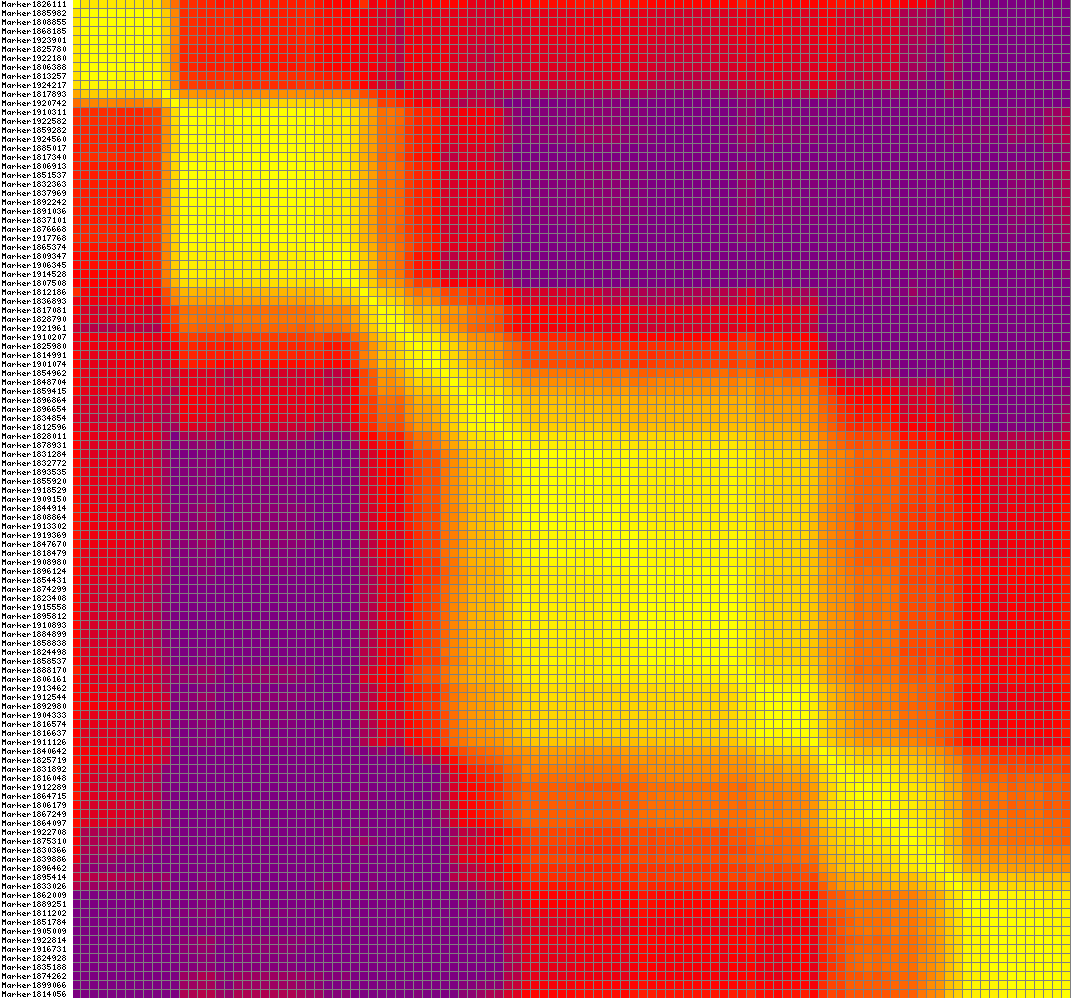

Supplement: Figure S4 — Heat map of the high-density genetic map. Each cell represents the recombination rate of two markers. Yellow indicates a lower recombination rate and purple a higher one. [file FigureS4.ZIP › Chr05.heatMap.png]

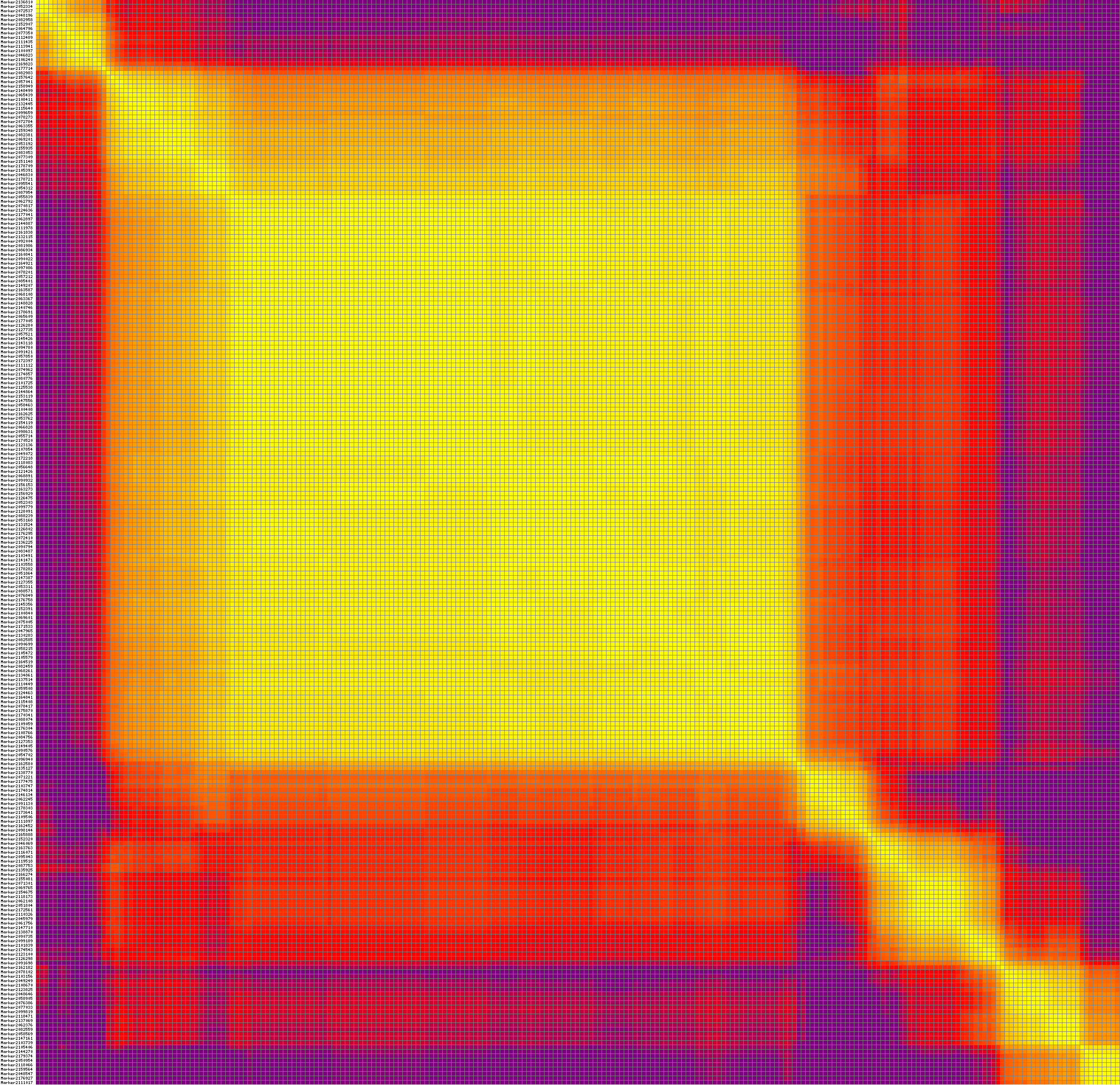

Supplement: Figure S4 — Heat map of the high-density genetic map. Each cell represents the recombination rate of two markers. Yellow indicates a lower recombination rate and purple a higher one. [file FigureS4.ZIP › Chr06.heatMap.png]

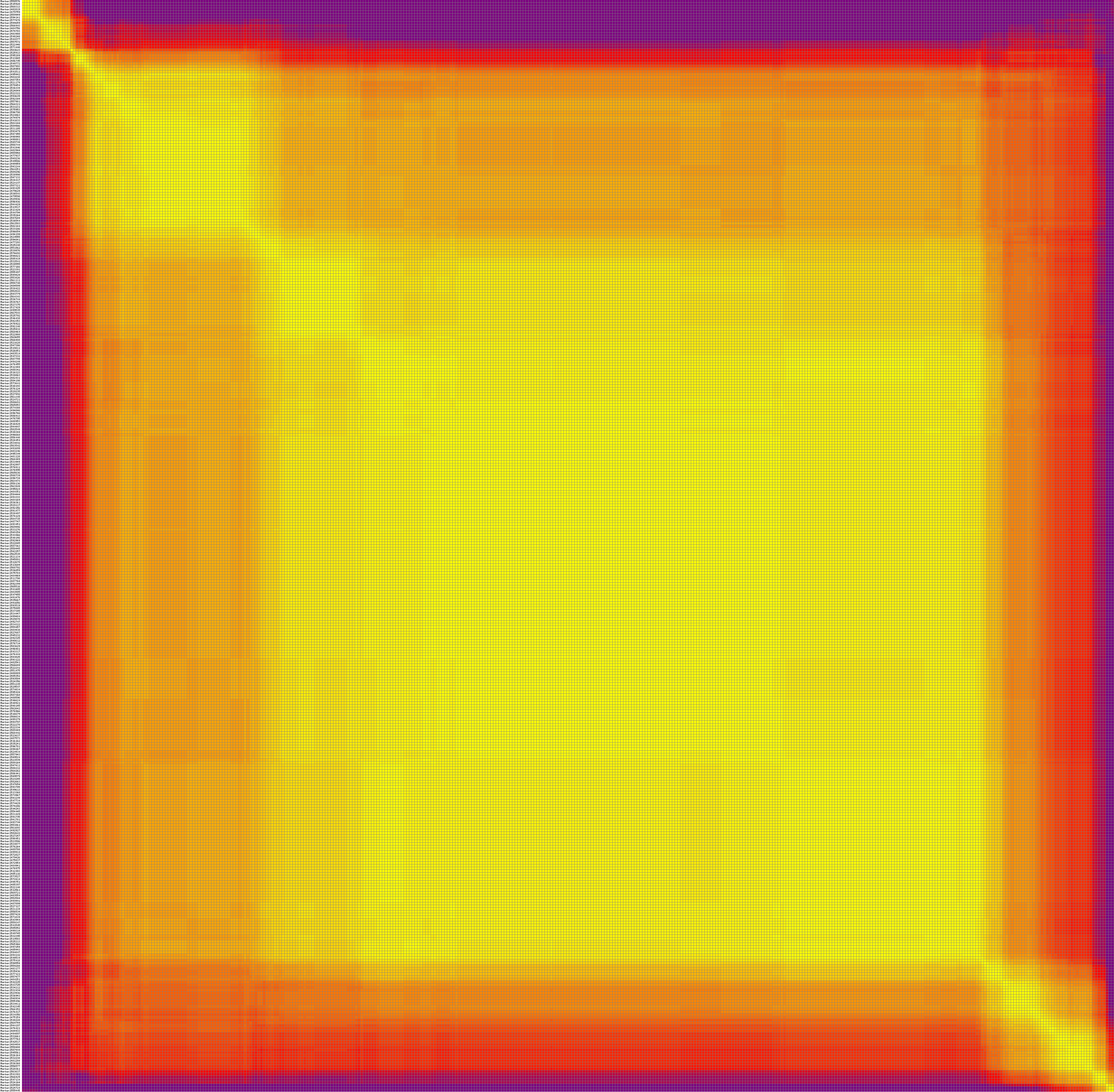

Supplement: Figure S4 — Heat map of the high-density genetic map. Each cell represents the recombination rate of two markers. Yellow indicates a lower recombination rate and purple a higher one. [file FigureS4.ZIP › Chr07.heatMap.png]

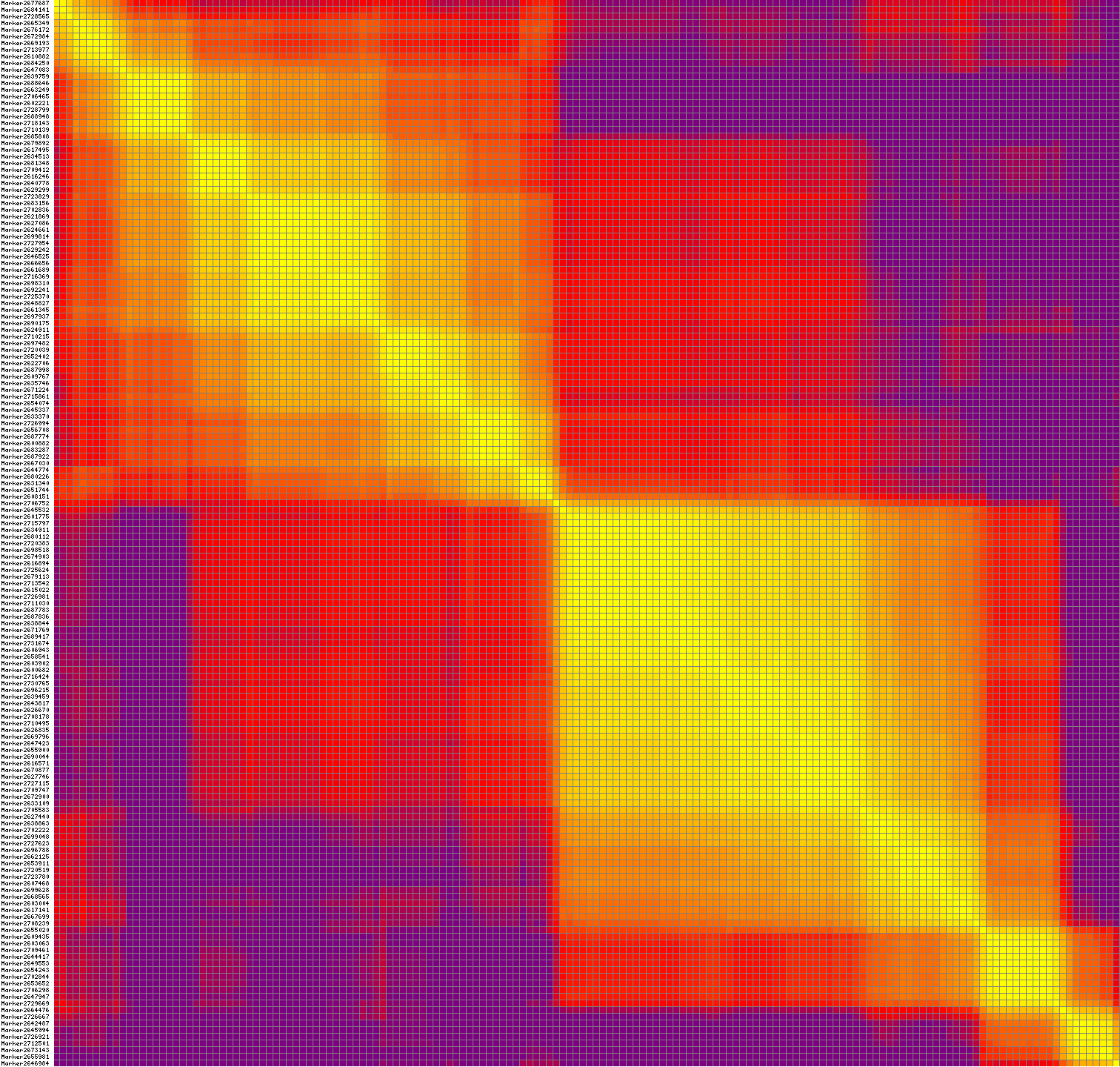

Supplement: Figure S4 — Heat map of the high-density genetic map. Each cell represents the recombination rate of two markers. Yellow indicates a lower recombination rate and purple a higher one. [file FigureS4.ZIP › Chr08.heatMap.png]

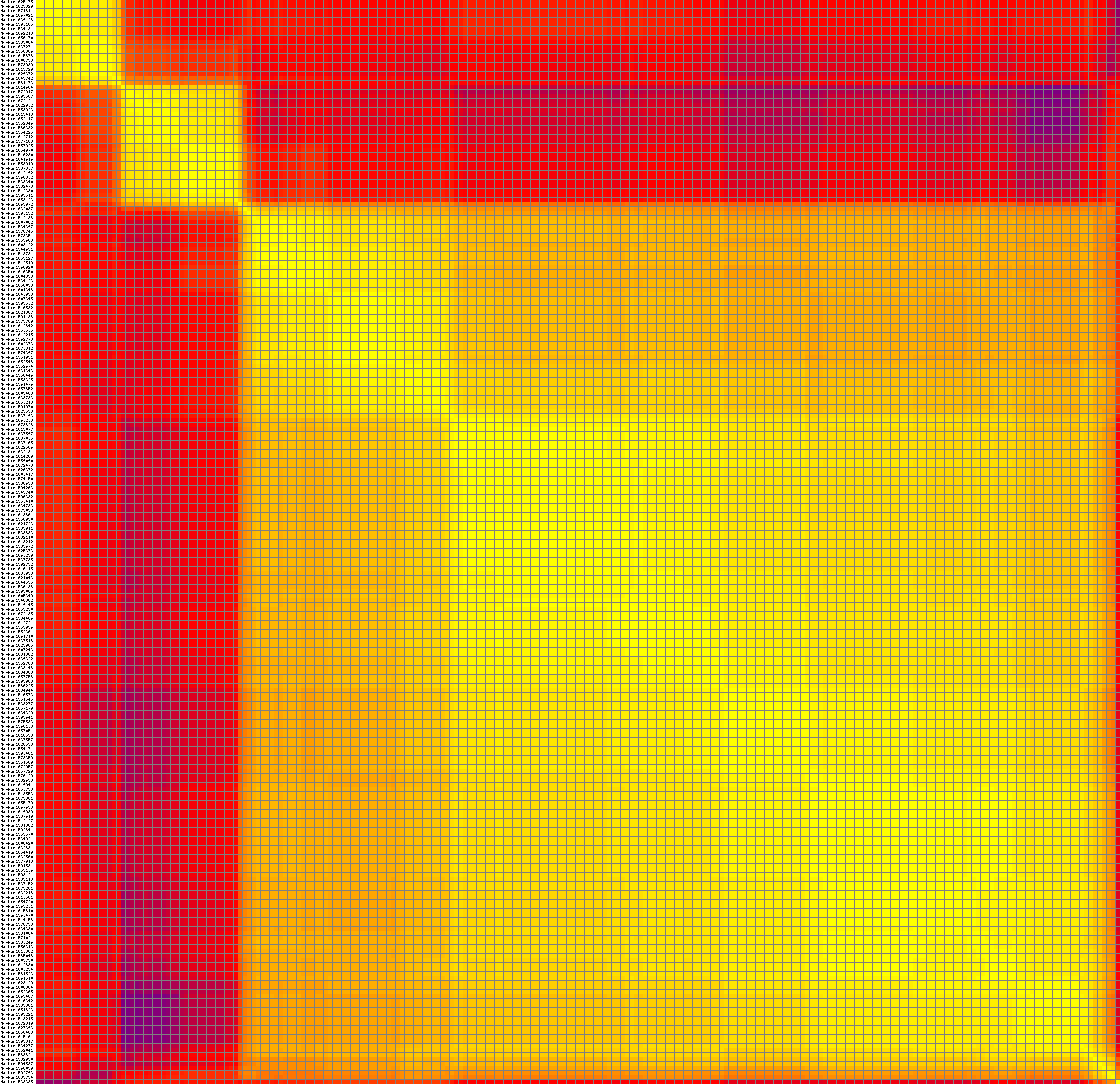

Supplement: Figure S4 — Heat map of the high-density genetic map. Each cell represents the recombination rate of two markers. Yellow indicates a lower recombination rate and purple a higher one. [file FigureS4.ZIP › Chr09.heatMap.png]

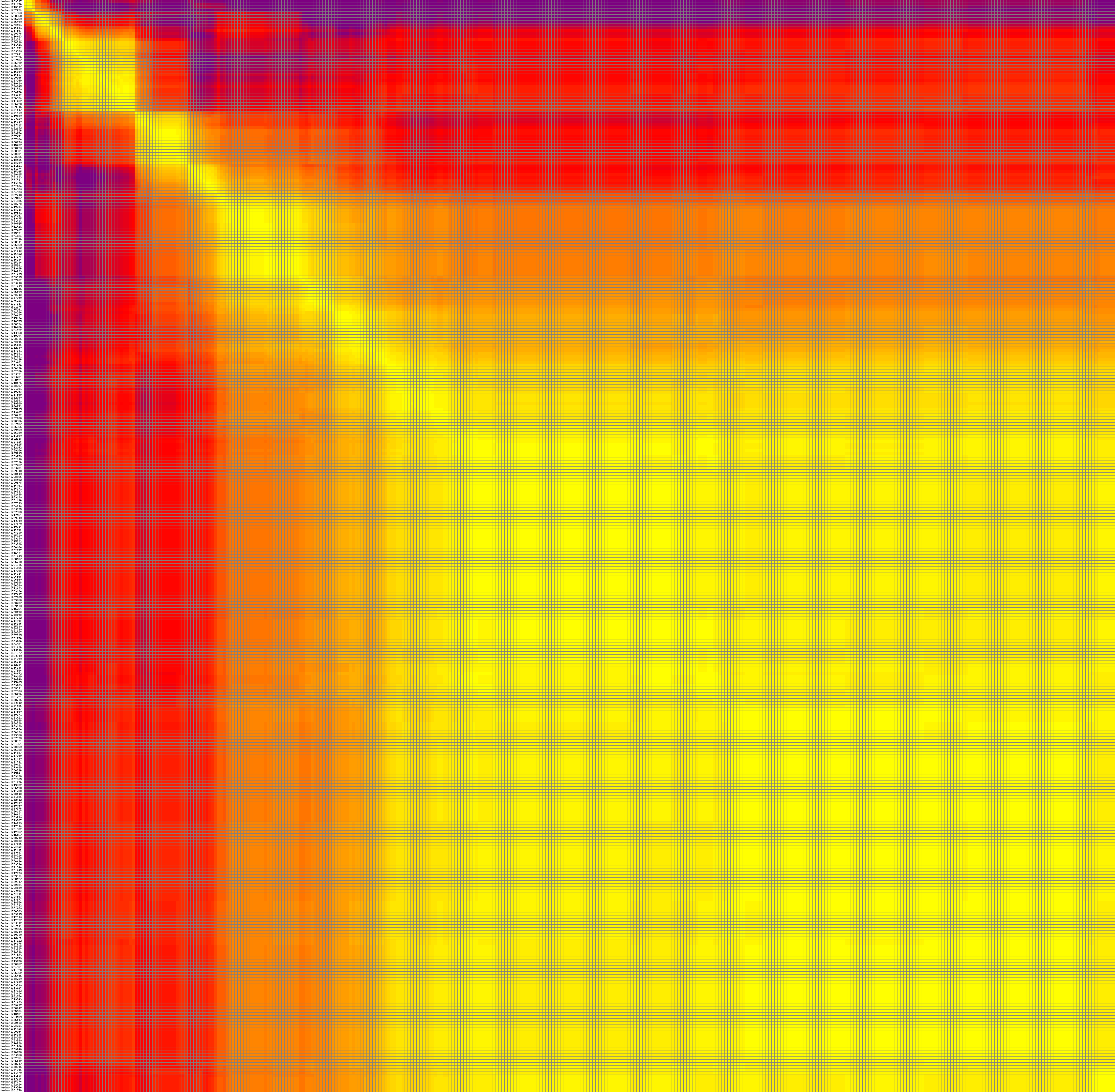

Supplement: Figure S4 — Heat map of the high-density genetic map. Each cell represents the recombination rate of two markers. Yellow indicates a lower recombination rate and purple a higher one. [file FigureS4.ZIP › Chr13.heatMap.png]

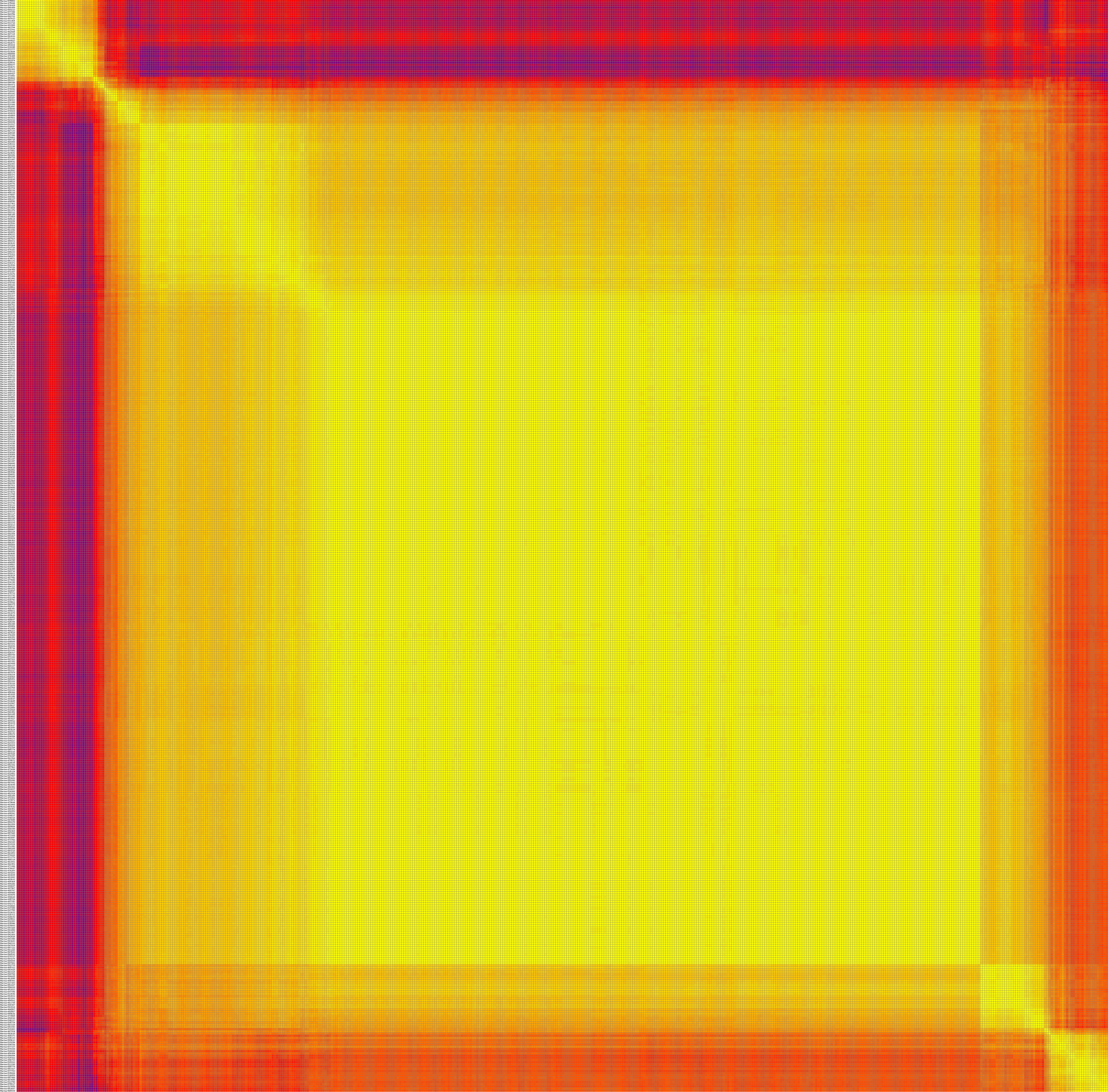

Supplement: Figure S4 — Heat map of the high-density genetic map. Each cell represents the recombination rate of two markers. Yellow indicates a lower recombination rate and purple a higher one. [file FigureS4.ZIP › Chr14.heatMap.png]

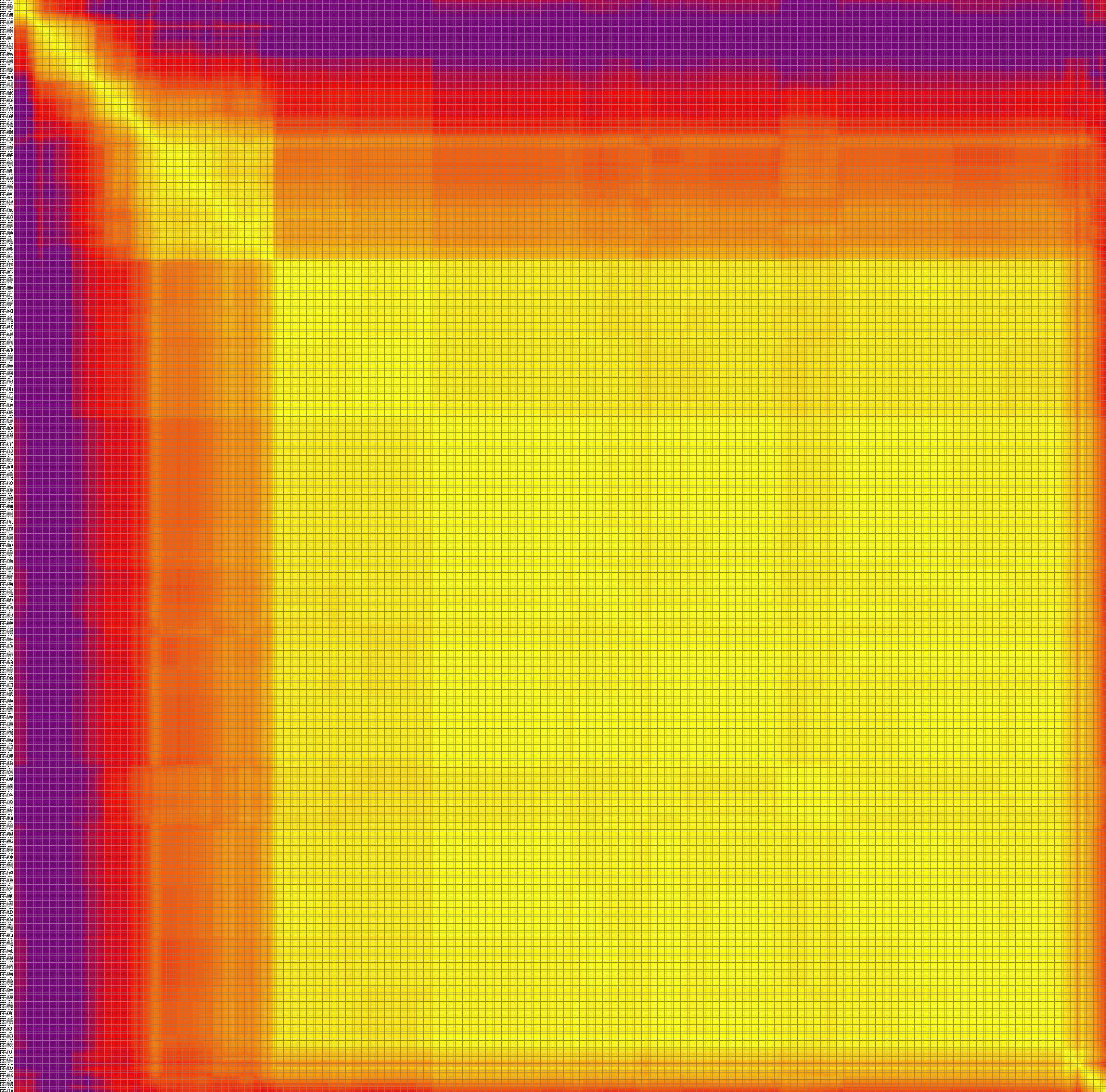

Supplement: Figure S4 — Heat map of the high-density genetic map. Each cell represents the recombination rate of two markers. Yellow indicates a lower recombination rate and purple a higher one. [file FigureS4.ZIP › Chr15.heatMap.png]

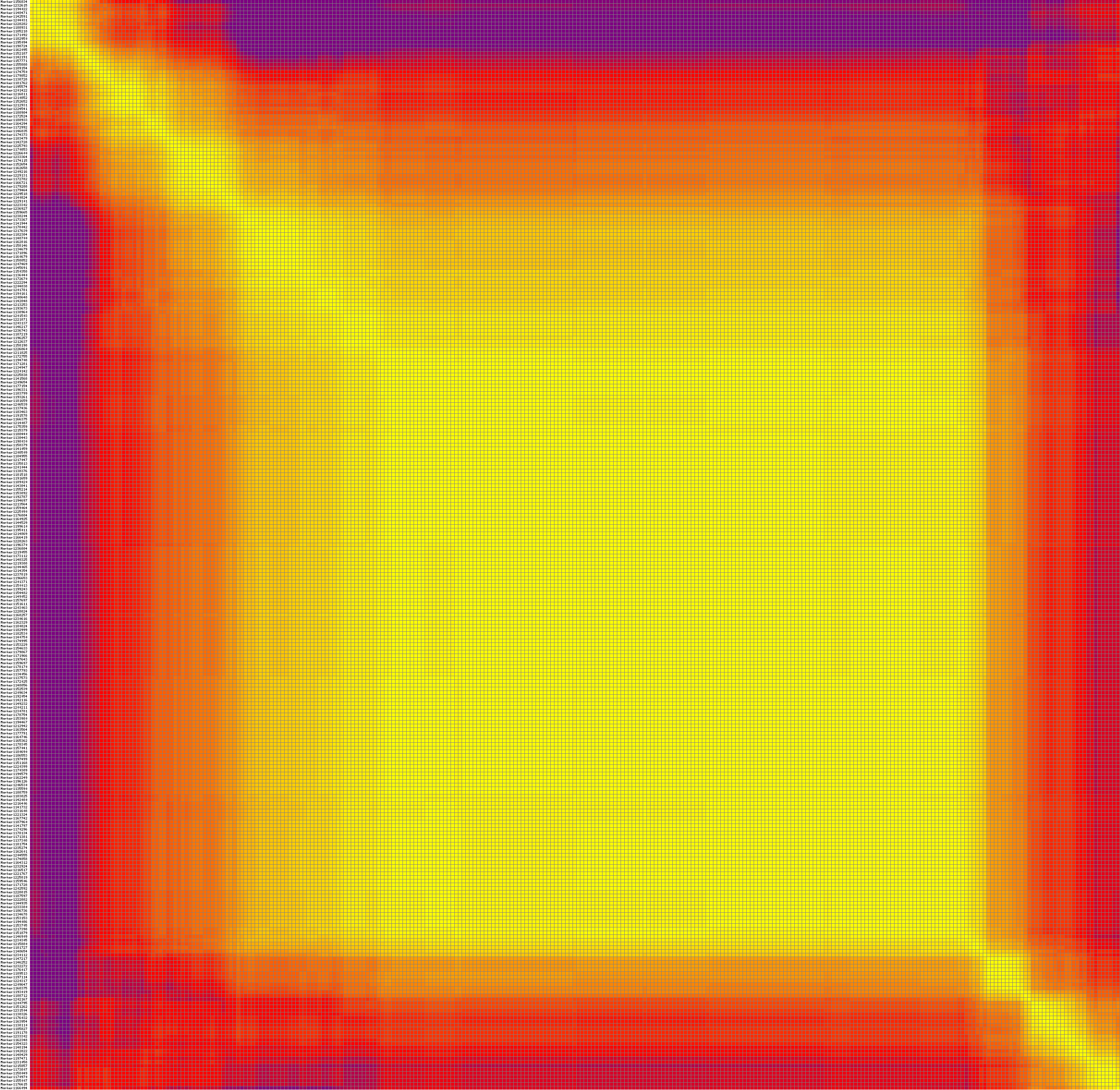

Supplement: Figure S4 — Heat map of the high-density genetic map. Each cell represents the recombination rate of two markers. Yellow indicates a lower recombination rate and purple a higher one. [file FigureS4.ZIP › Chr16.heatMap.png]

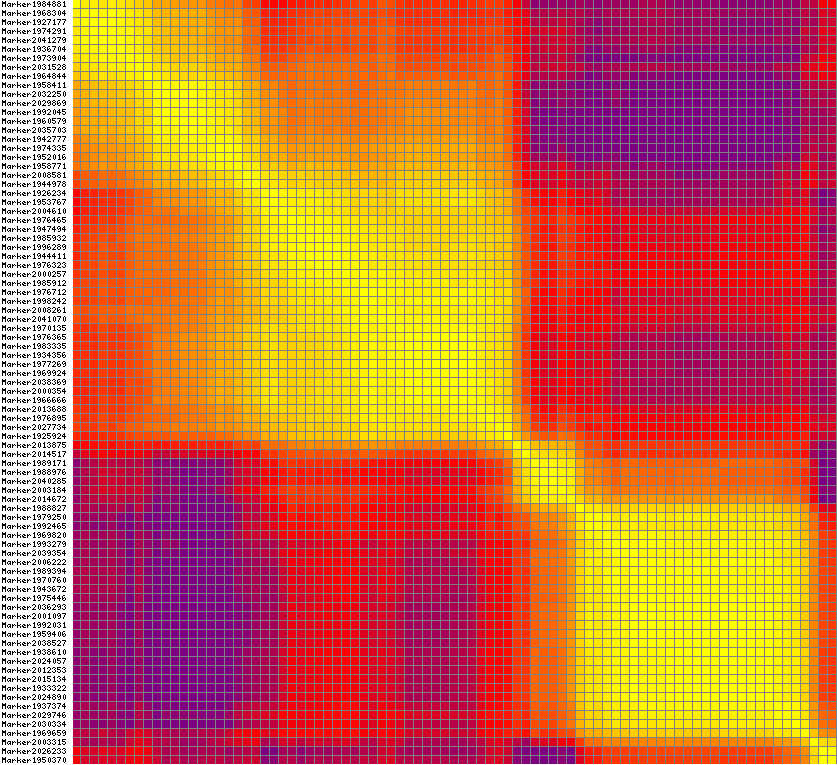

Supplement: Figure S4 — Heat map of the high-density genetic map. Each cell represents the recombination rate of two markers. Yellow indicates a lower recombination rate and purple a higher one. [file FigureS4.ZIP › Chr17.heatMap.png]

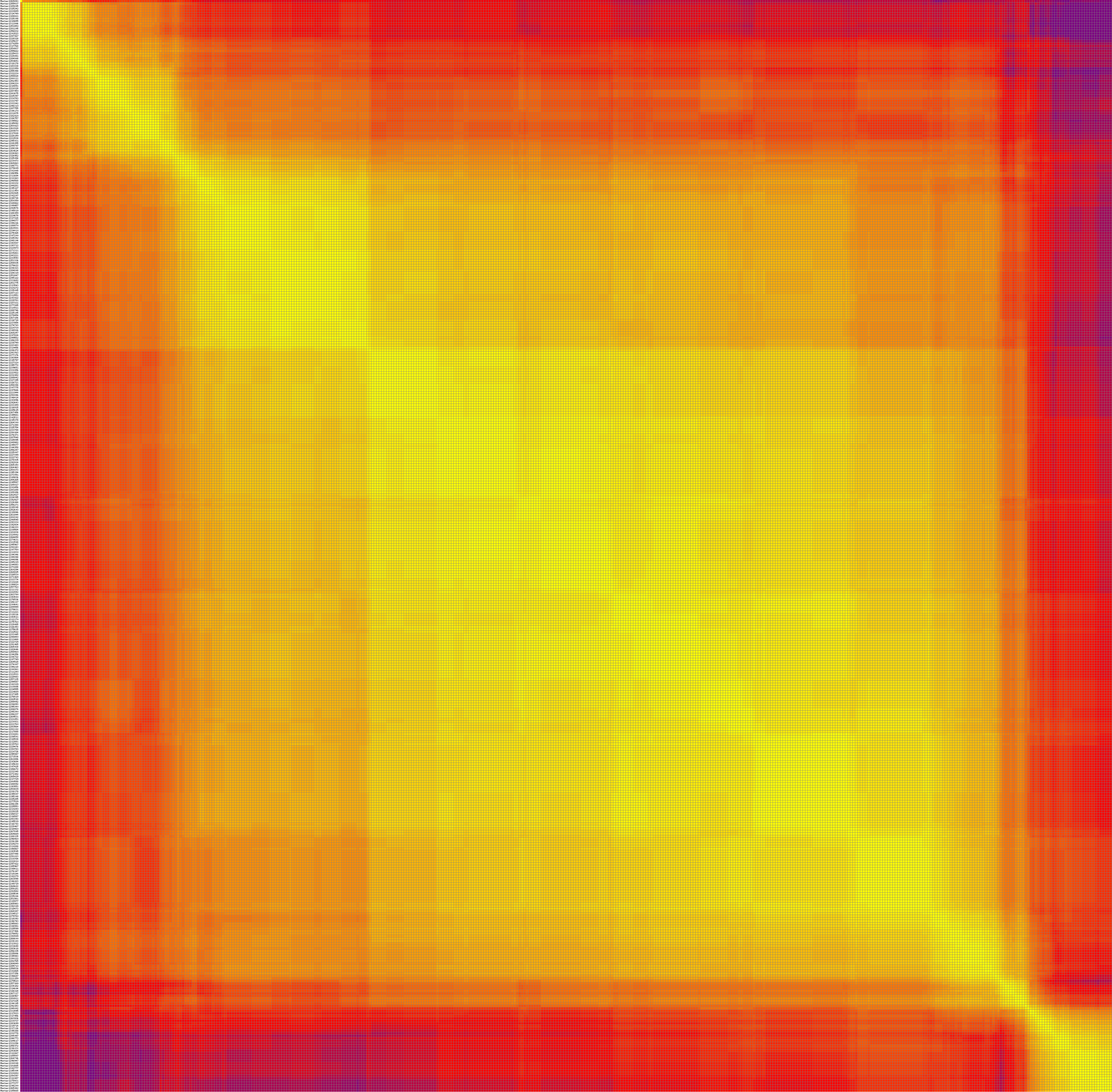

Supplement: Figure S4 — Heat map of the high-density genetic map. Each cell represents the recombination rate of two markers. Yellow indicates a lower recombination rate and purple a higher one. [file FigureS4.ZIP › Chr18.heatMap.png]

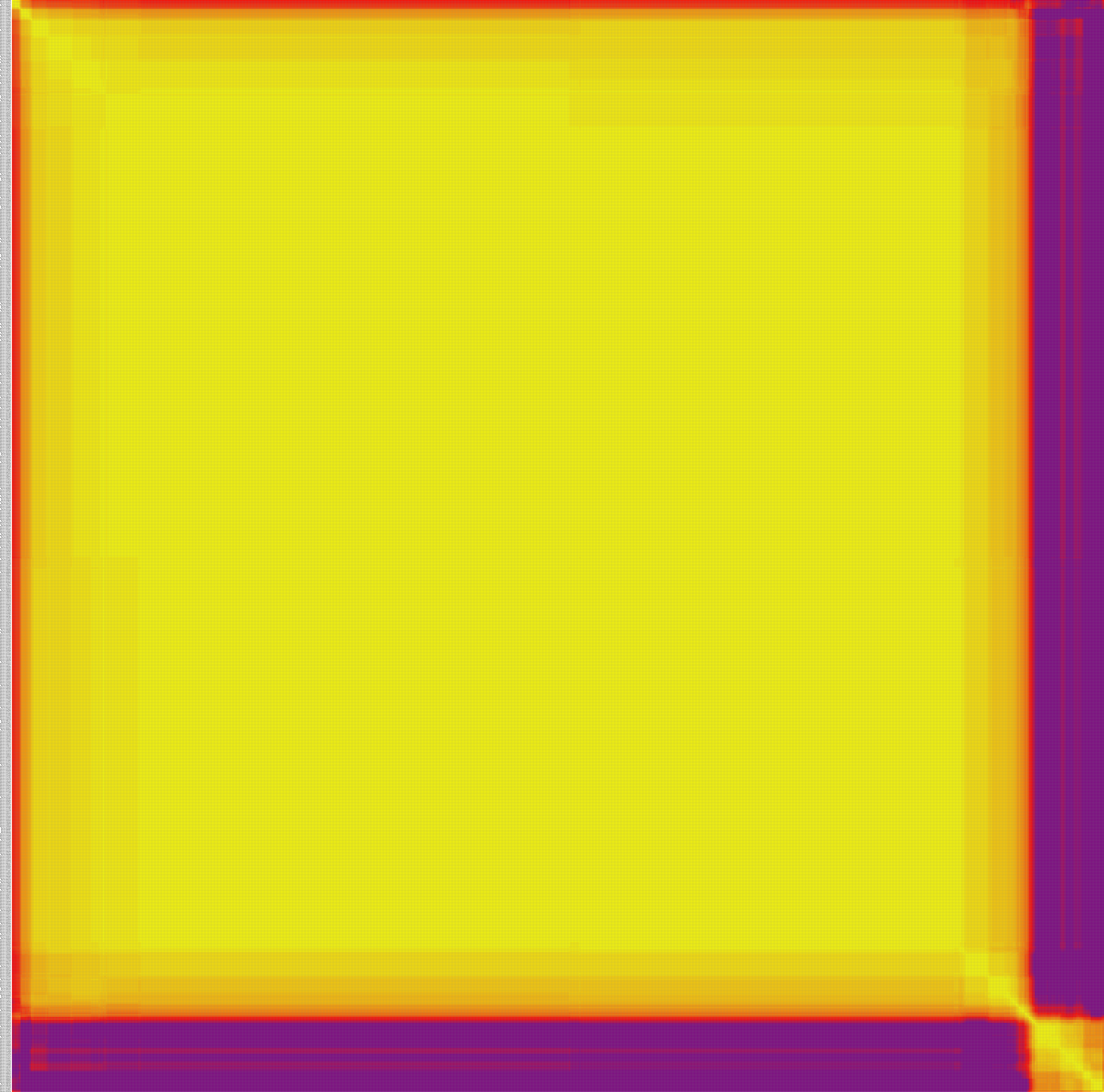

Supplement: Figure S4 — Heat map of the high-density genetic map. Each cell represents the recombination rate of two markers. Yellow indicates a lower recombination rate and purple a higher one. [file FigureS4.ZIP › Chr19.heatMap.png]

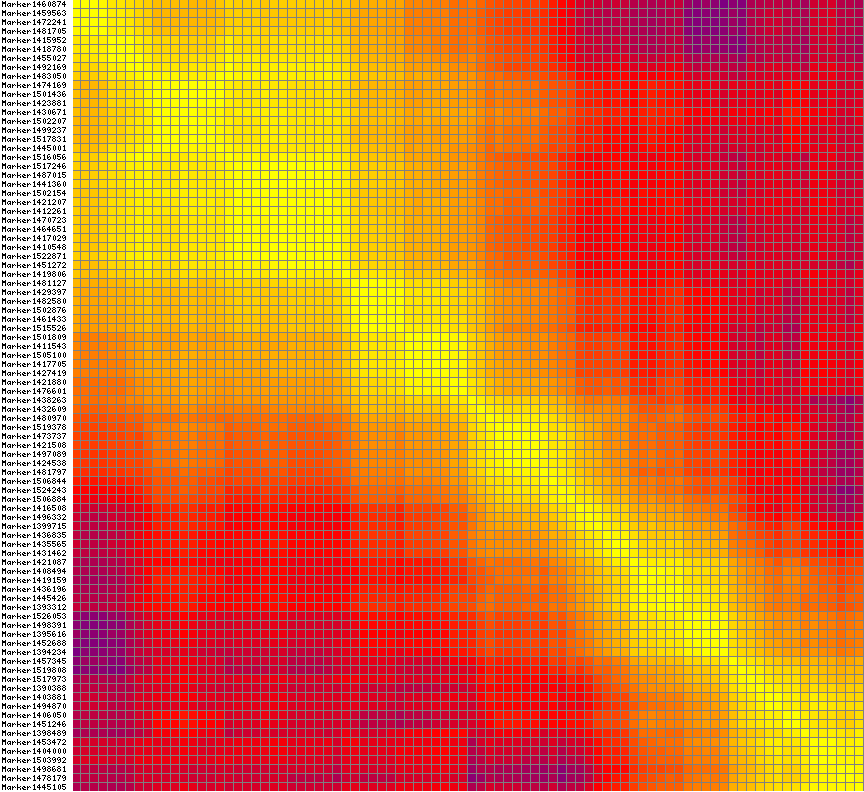

Supplement: Figure S4 — Heat map of the high-density genetic map. Each cell represents the recombination rate of two markers. Yellow indicates a lower recombination rate and purple a higher one. [file FigureS4.ZIP › Chr20.heatMap.png]

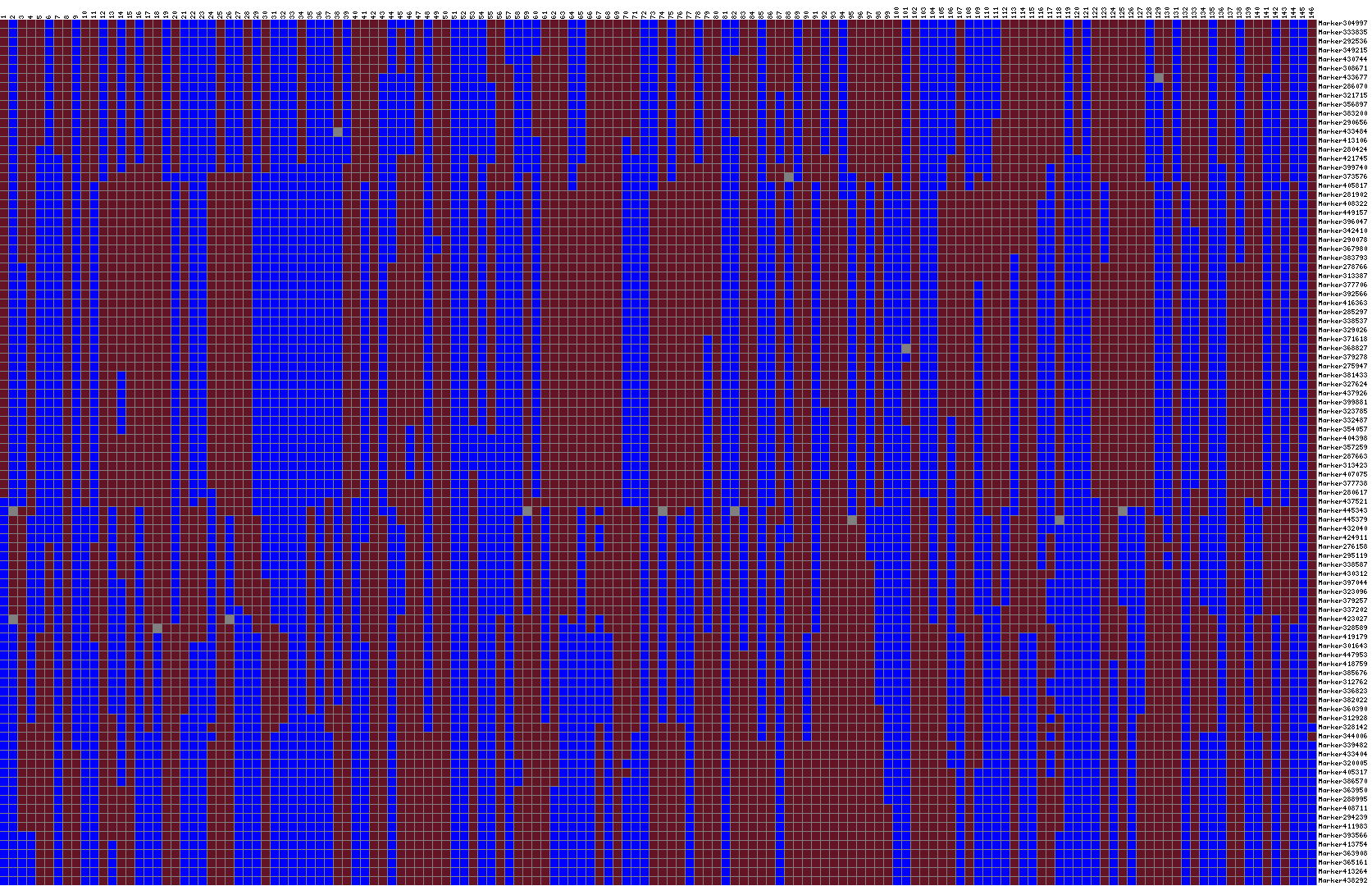

Supplement: Figure S5 — Haplotype map of the genetic map. Blue represents Nannong94-156, red represents Bogao, white means the parent could not be estimated, gray represents deletions. [file FigureS5.ZIP › Chr01.loc.haplo.png]

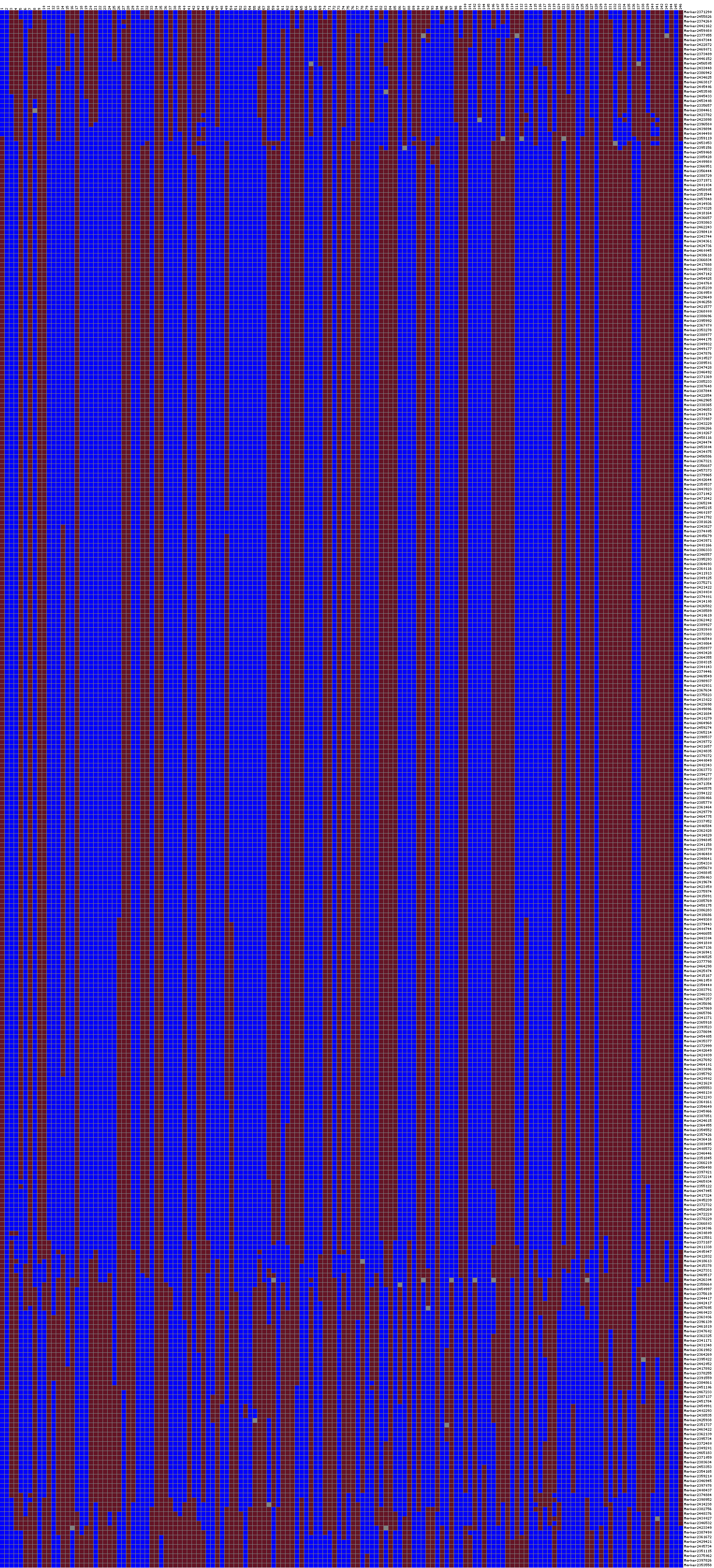

Supplement: Figure S5 — Haplotype map of the genetic map. Blue represents Nannong94-156, red represents Bogao, white means the parent could not be estimated, gray represents deletions. [file FigureS5.ZIP › Chr02.loc.haplo.png]

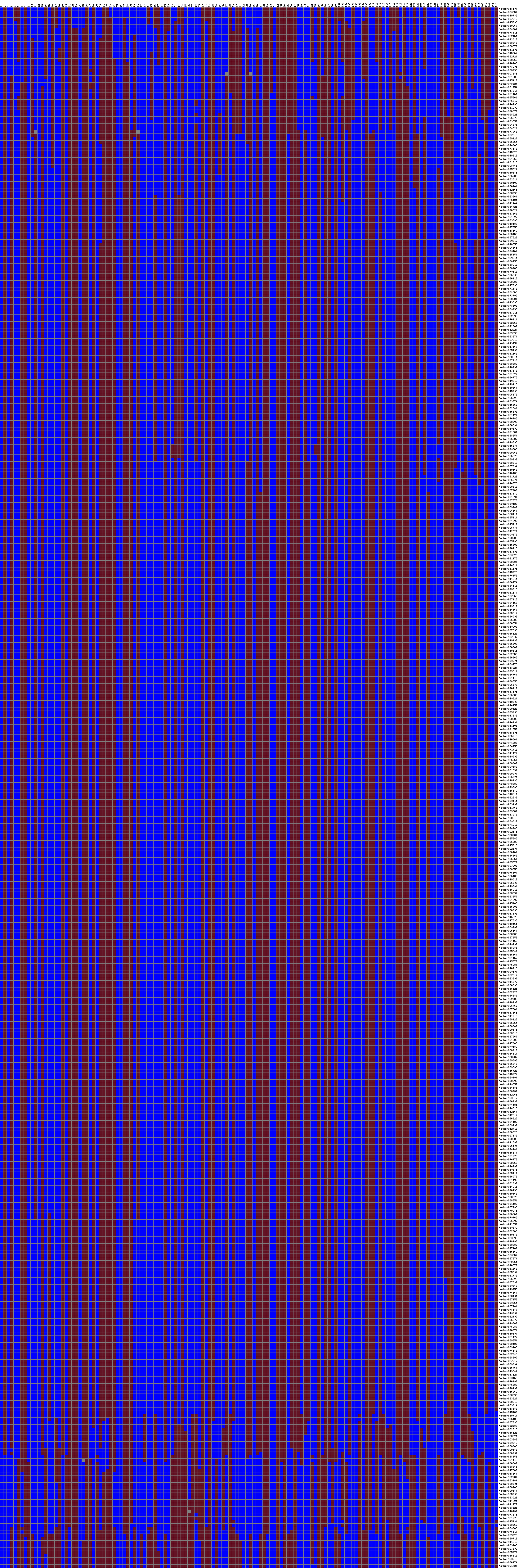

Supplement: Figure S5 — Haplotype map of the genetic map. Blue represents Nannong94-156, red represents Bogao, white means the parent could not be estimated, gray represents deletions. [file FigureS5.ZIP › Chr03.loc.haplo.png]

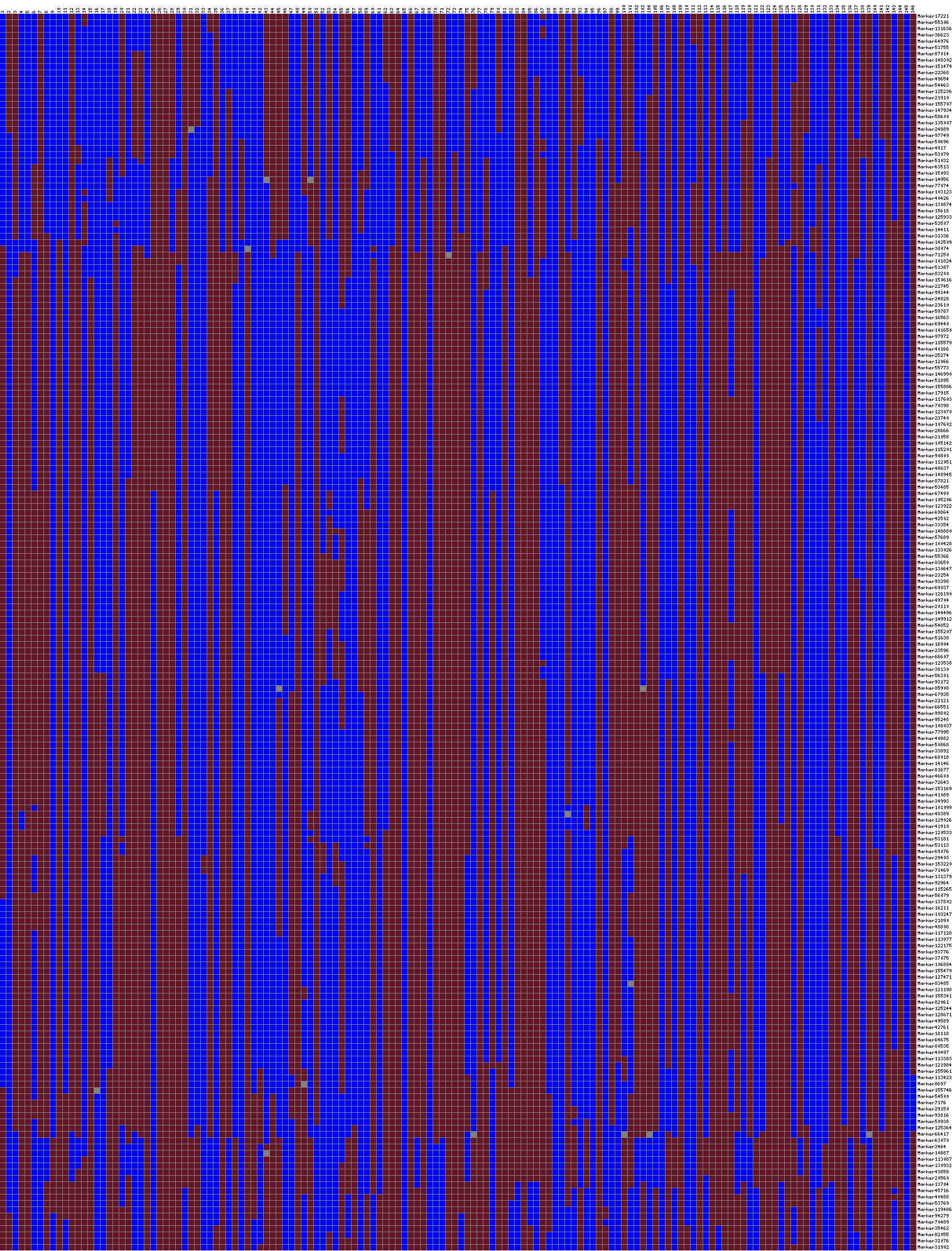

Supplement: Figure S5 — Haplotype map of the genetic map. Blue represents Nannong94-156, red represents Bogao, white means the parent could not be estimated, gray represents deletions. [file FigureS5.ZIP › Chr04.loc.haplo.png]

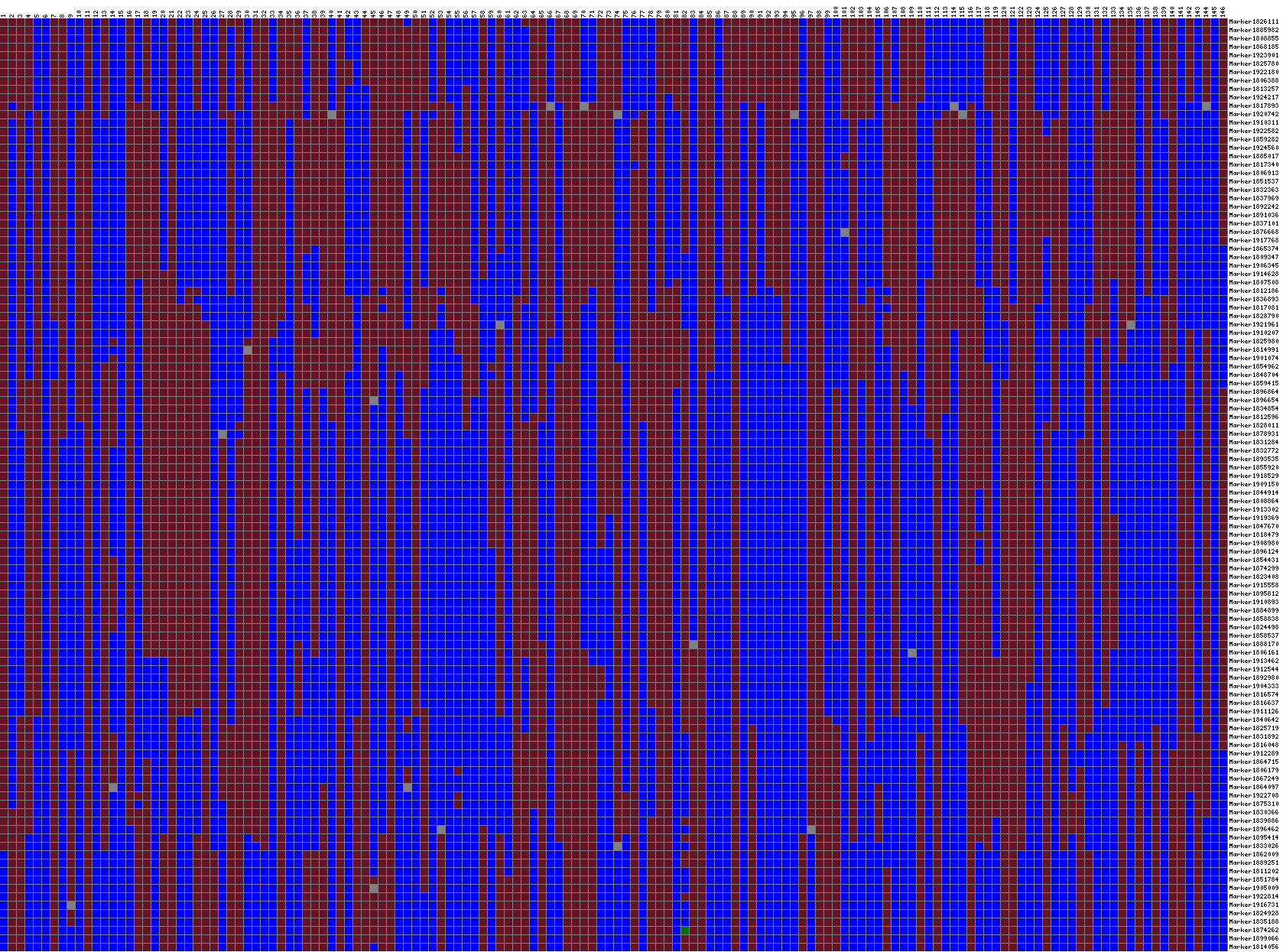

Supplement: Figure S5 — Haplotype map of the genetic map. Blue represents Nannong94-156, red represents Bogao, white means the parent could not be estimated, gray represents deletions. [file FigureS5.ZIP › Chr05.loc.haplo.png]

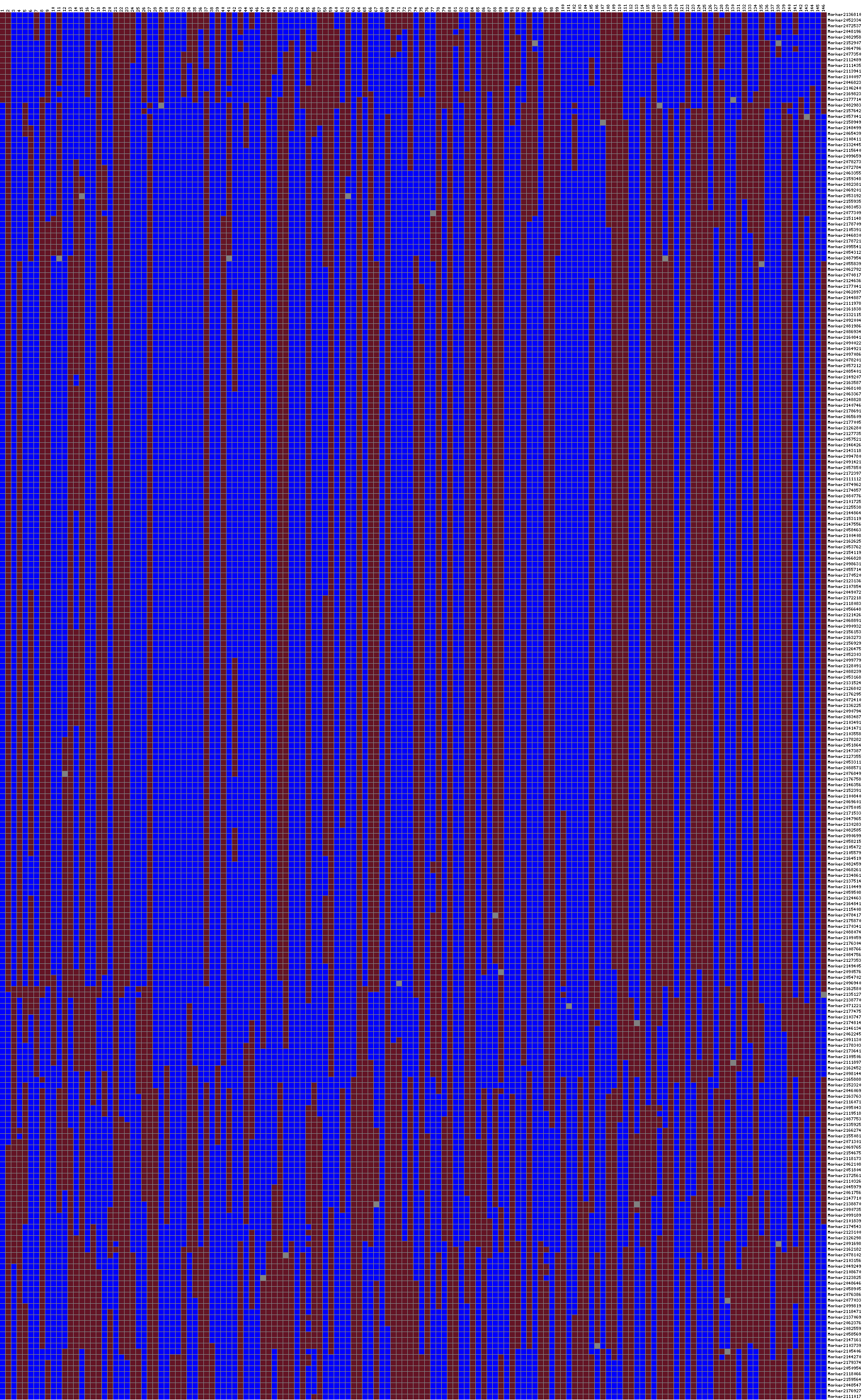

Supplement: Figure S5 — Haplotype map of the genetic map. Blue represents Nannong94-156, red represents Bogao, white means the parent could not be estimated, gray represents deletions. [file FigureS5.ZIP › Chr06.loc.haplo.png]

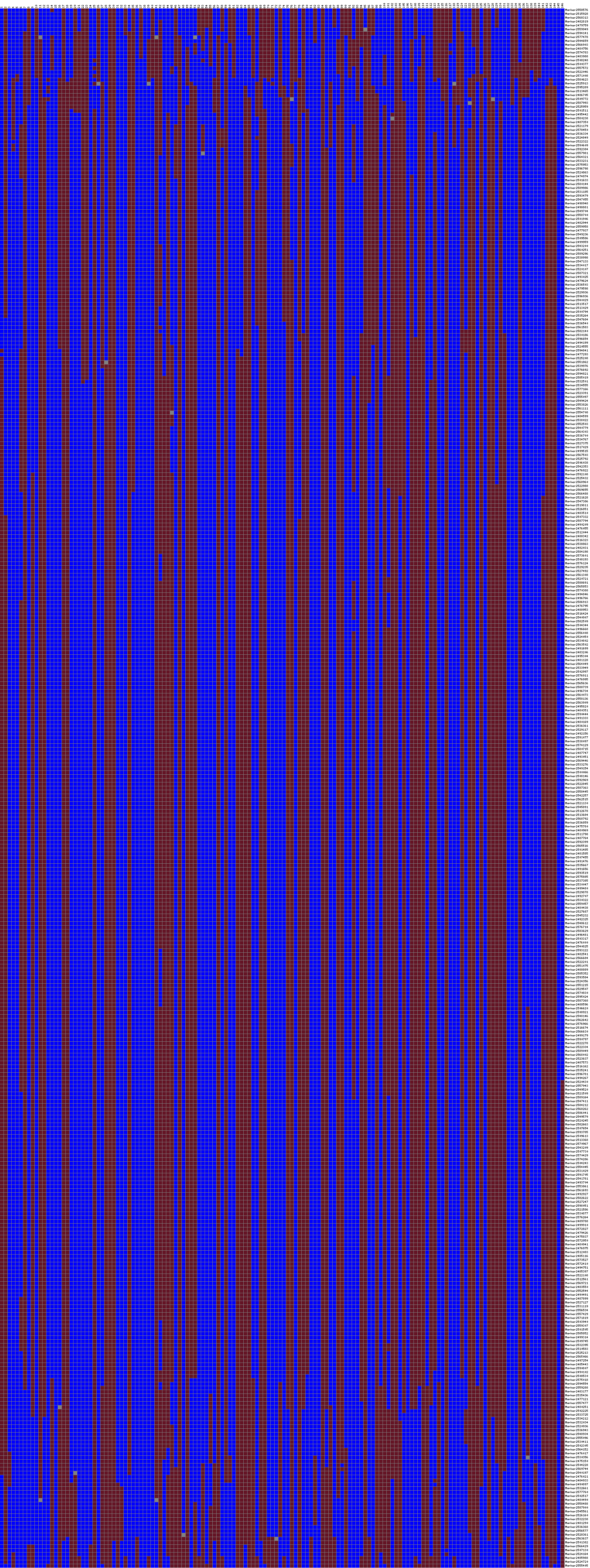

Supplement: Figure S5 — Haplotype map of the genetic map. Blue represents Nannong94-156, red represents Bogao, white means the parent could not be estimated, gray represents deletions. [file FigureS5.ZIP › Chr07.loc.haplo.png]

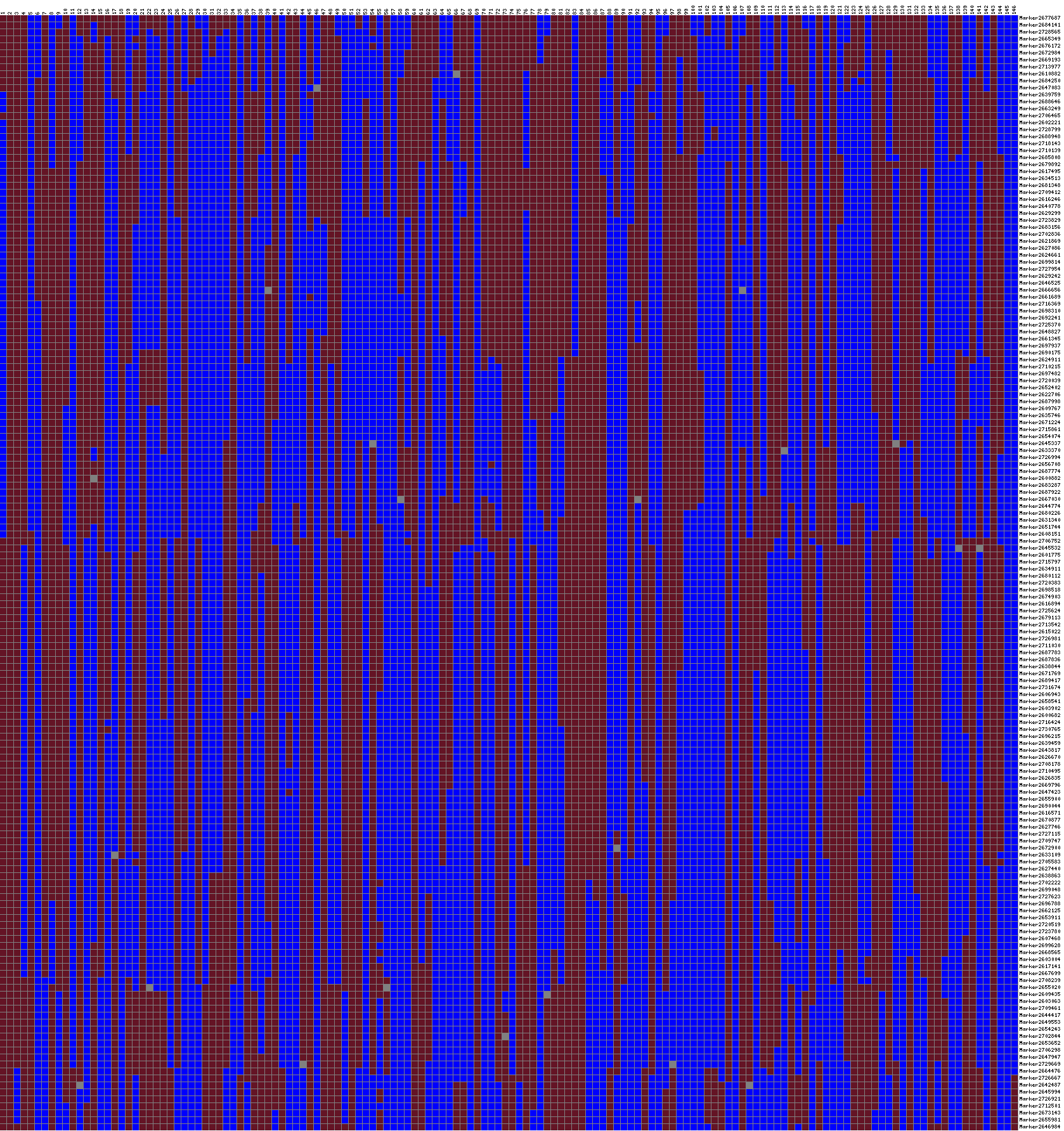

Supplement: Figure S5 — Haplotype map of the genetic map. Blue represents Nannong94-156, red represents Bogao, white means the parent could not be estimated, gray represents deletions. [file FigureS5.ZIP › Chr08.loc.haplo.png]

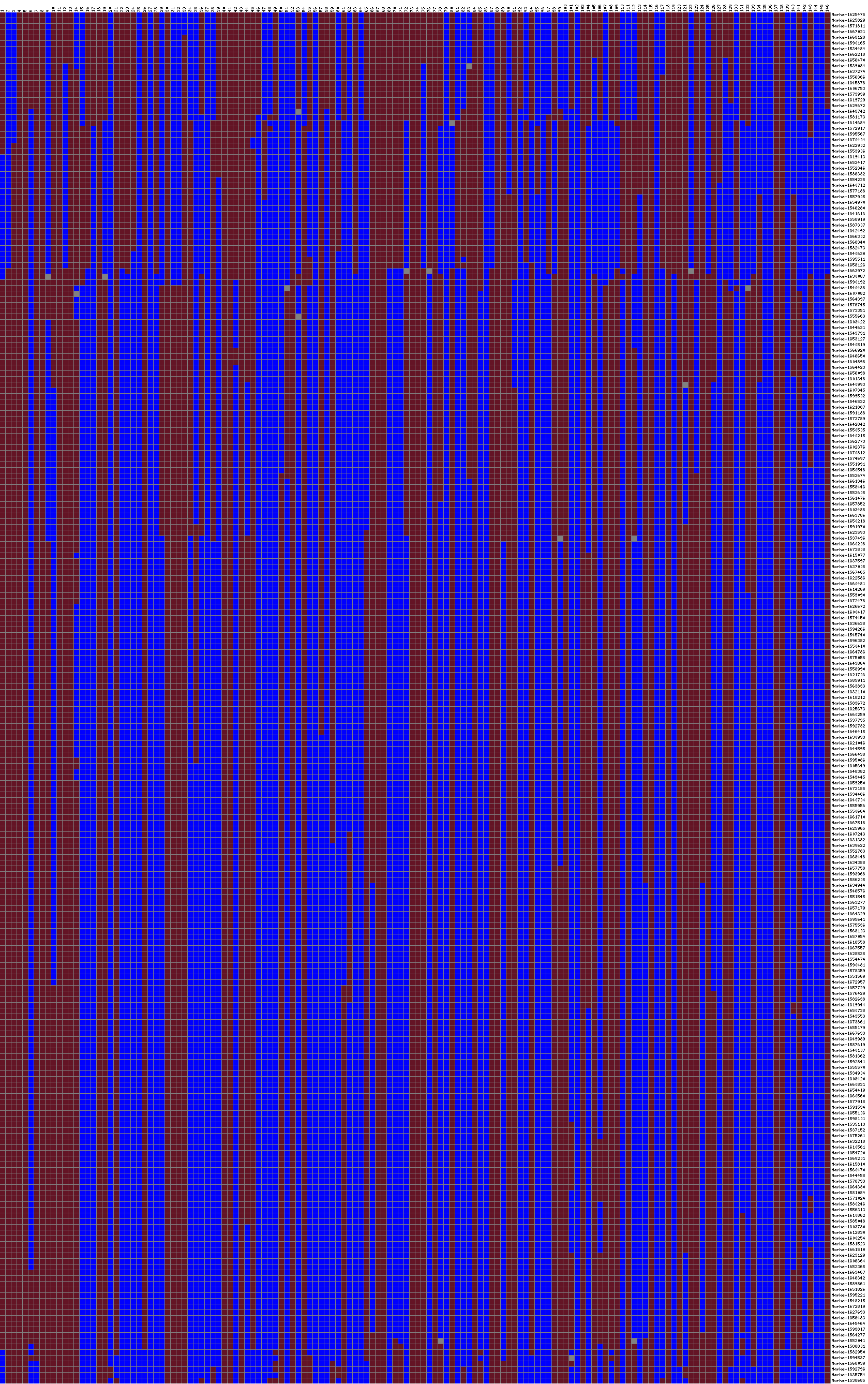

Supplement: Figure S5 — Haplotype map of the genetic map. Blue represents Nannong94-156, red represents Bogao, white means the parent could not be estimated, gray represents deletions. [file FigureS5.ZIP › Chr09.loc.haplo.png]

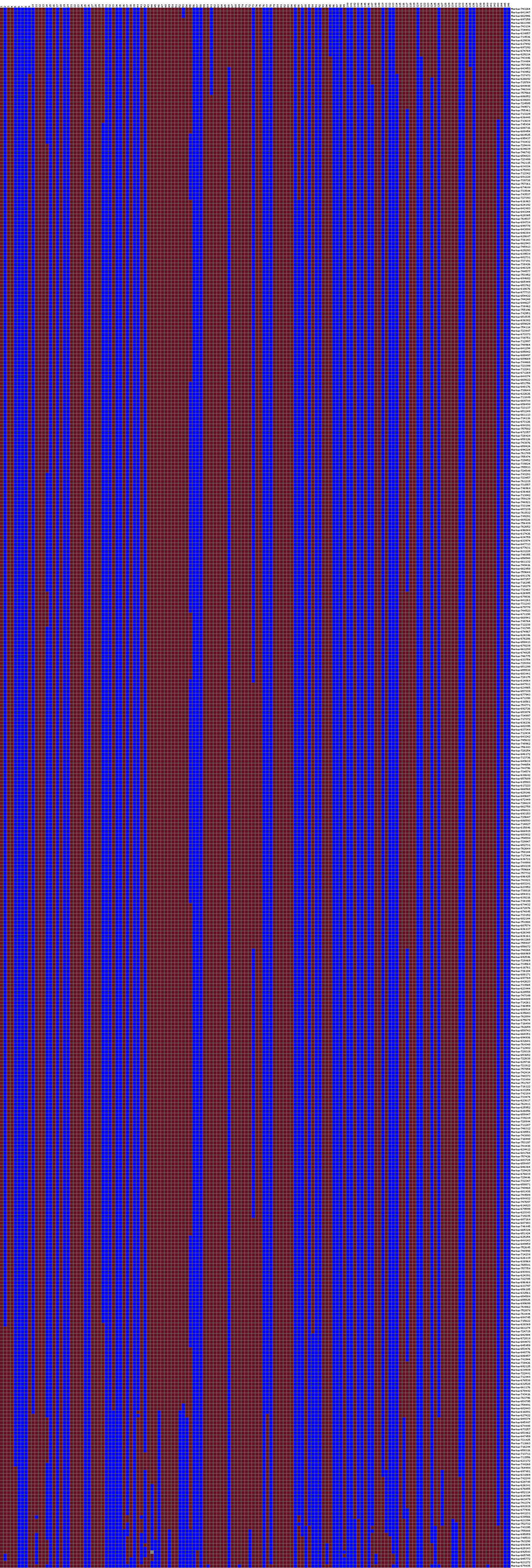

Supplement: Figure S5 — Haplotype map of the genetic map. Blue represents Nannong94-156, red represents Bogao, white means the parent could not be estimated, gray represents deletions. [file FigureS5.ZIP › Chr10.loc.haplo.png]

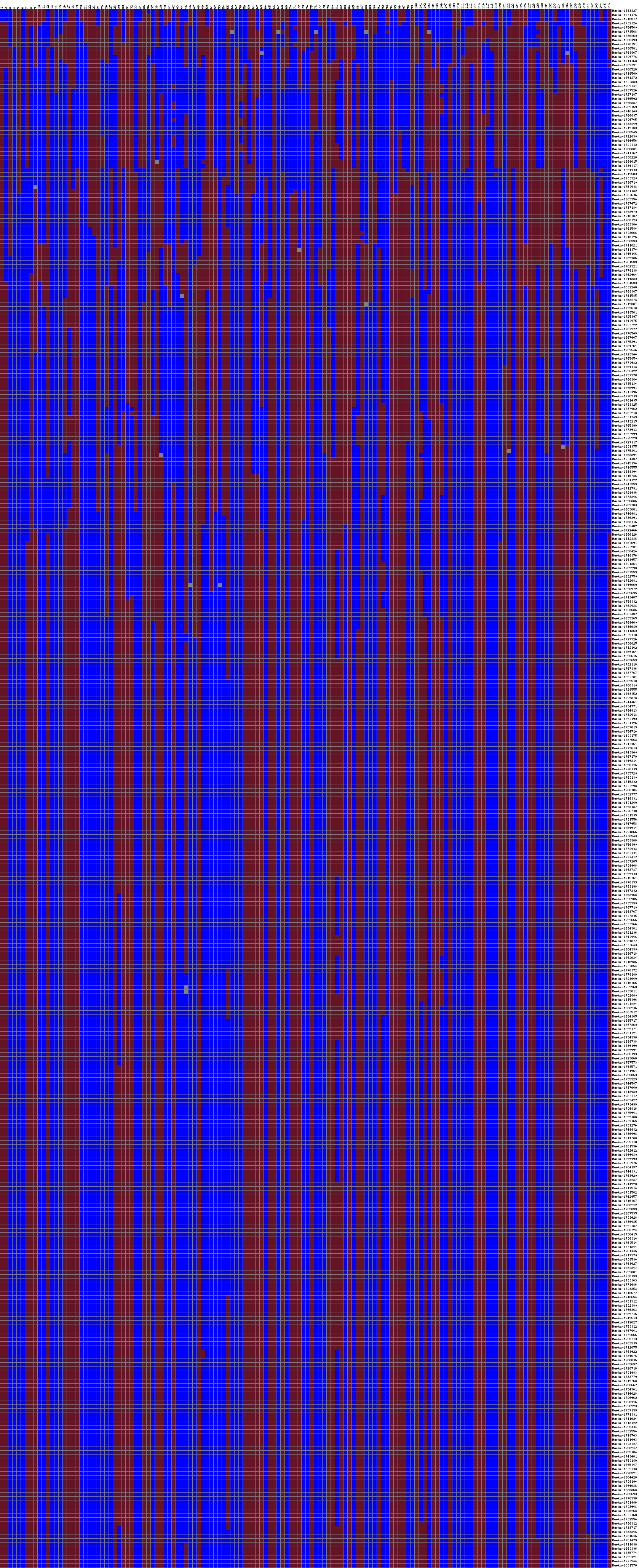

Supplement: Figure S5 — Haplotype map of the genetic map. Blue represents Nannong94-156, red represents Bogao, white means the parent could not be estimated, gray represents deletions. [file FigureS5.ZIP › Chr13.loc.haplo.png]

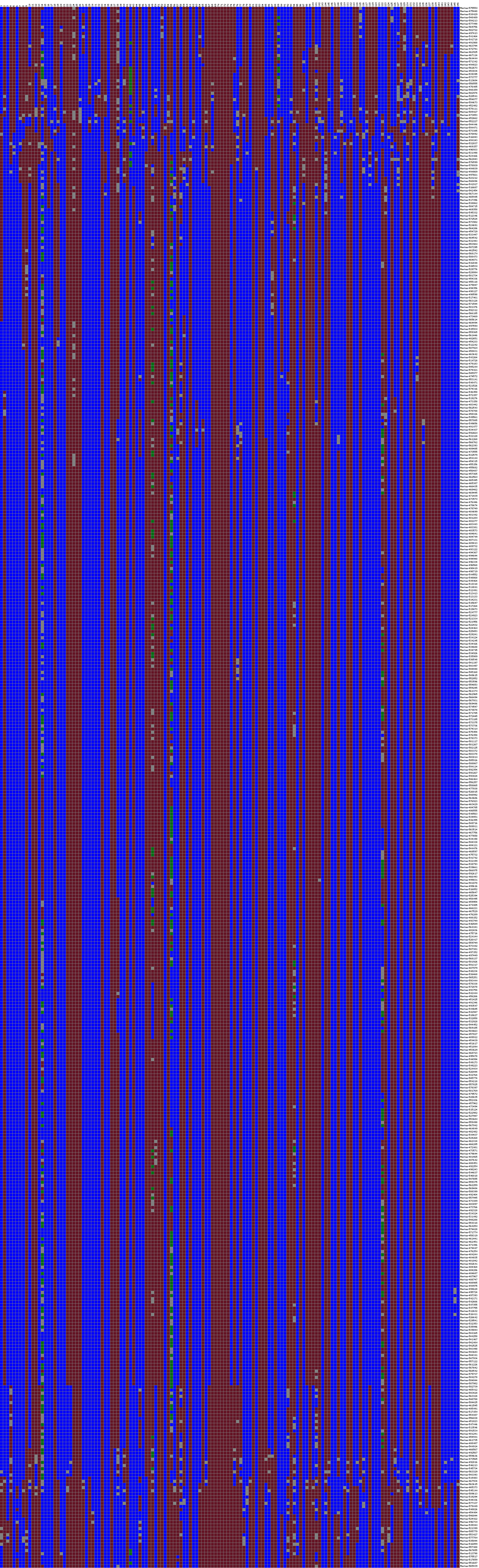

Supplement: Figure S5 — Haplotype map of the genetic map. Blue represents Nannong94-156, red represents Bogao, white means the parent could not be estimated, gray represents deletions. [file FigureS5.ZIP › Chr14.loc.haplo.png]

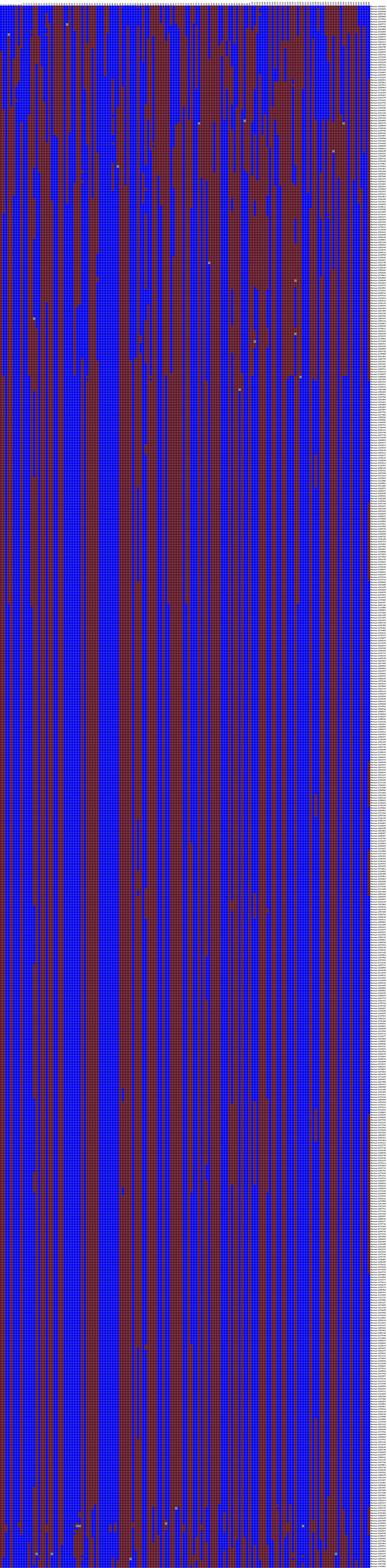

Supplement: Figure S5 — Haplotype map of the genetic map. Blue represents Nannong94-156, red represents Bogao, white means the parent could not be estimated, gray represents deletions. [file FigureS5.ZIP › Chr15.loc.haplo.png]

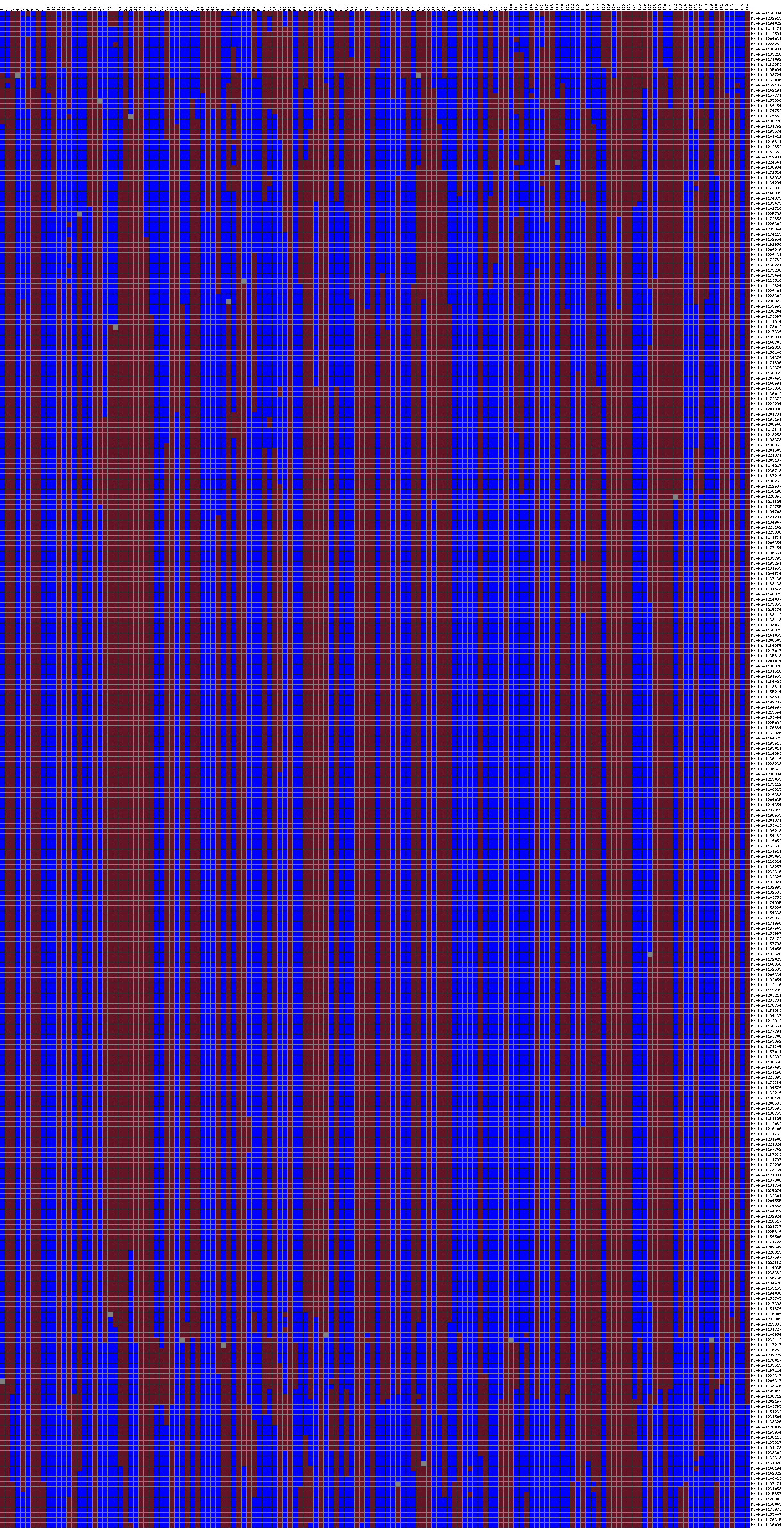

Supplement: Figure S5 — Haplotype map of the genetic map. Blue represents Nannong94-156, red represents Bogao, white means the parent could not be estimated, gray represents deletions. [file FigureS5.ZIP › Chr16.loc.haplo.png]

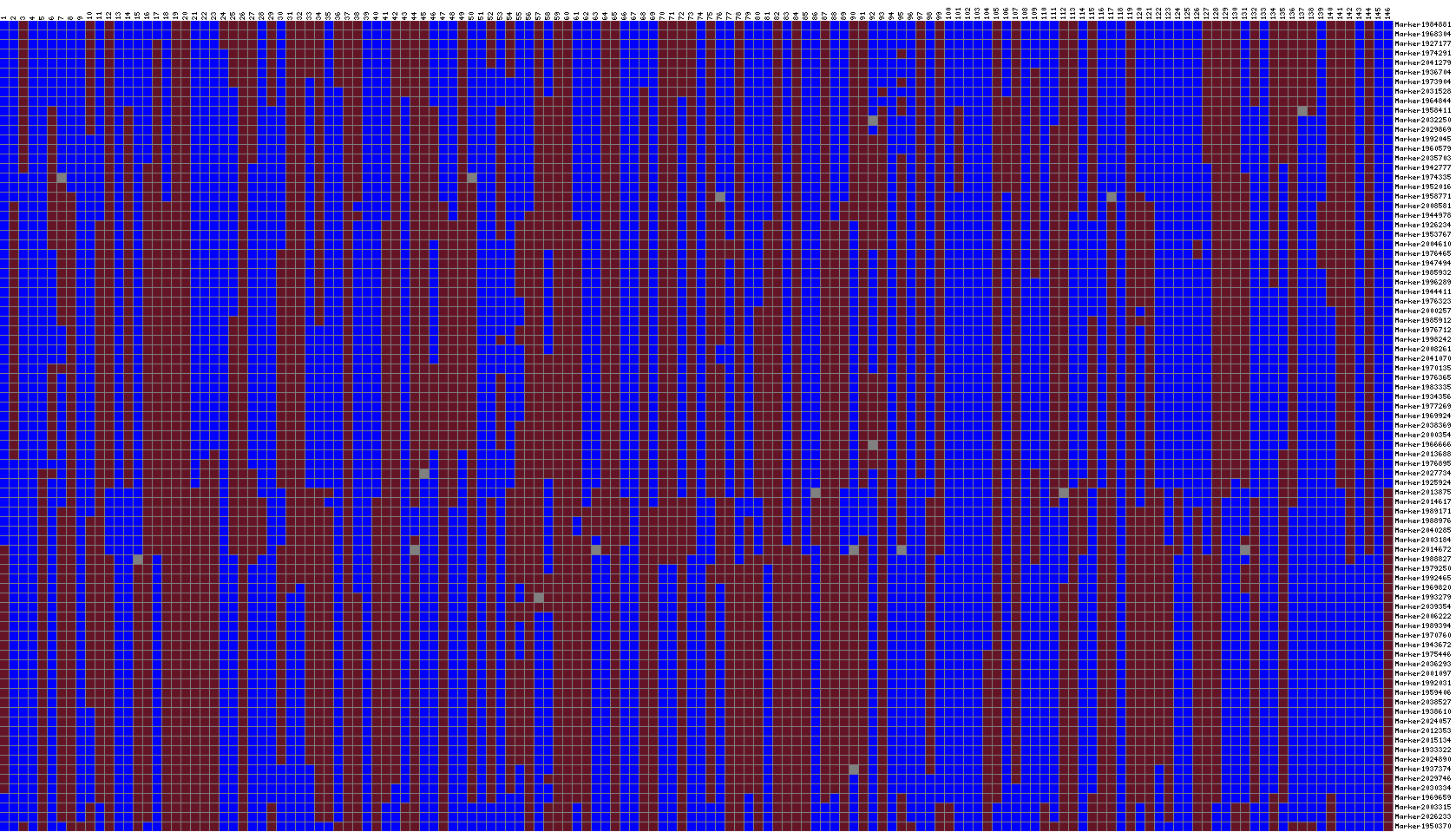

Supplement: Figure S5 — Haplotype map of the genetic map. Blue represents Nannong94-156, red represents Bogao, white means the parent could not be estimated, gray represents deletions. [file FigureS5.ZIP › Chr17.loc.haplo.png]

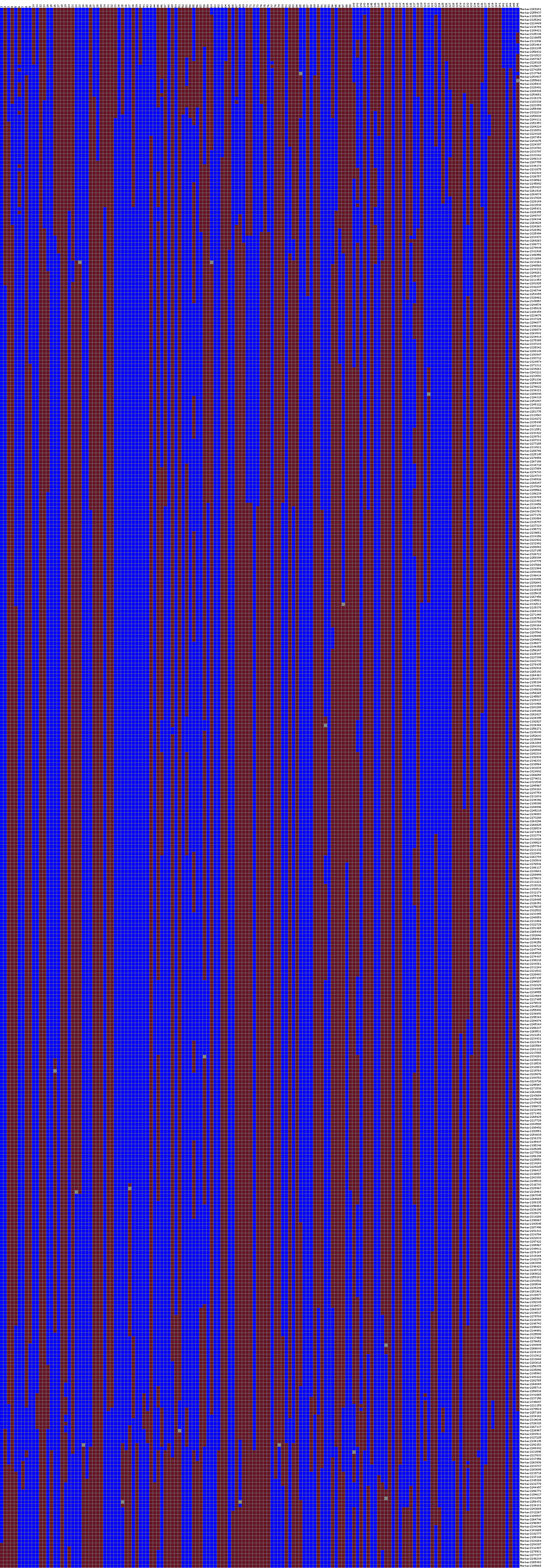

Supplement: Figure S5 — Haplotype map of the genetic map. Blue represents Nannong94-156, red represents Bogao, white means the parent could not be estimated, gray represents deletions. [file FigureS5.ZIP › Chr18.loc.haplo.png]

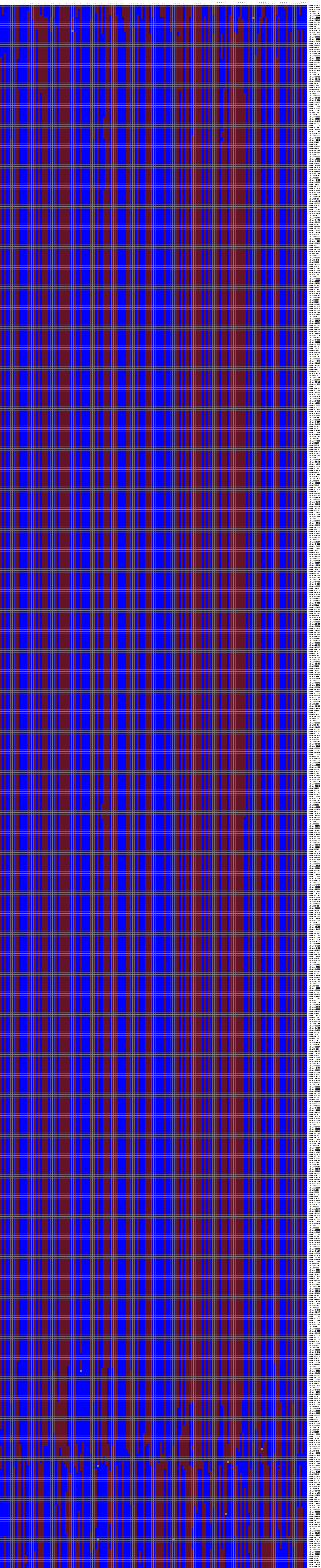

Supplement: Figure S5 — Haplotype map of the genetic map. Blue represents Nannong94-156, red represents Bogao, white means the parent could not be estimated, gray represents deletions. [file FigureS5.ZIP › Chr19.loc.haplo.png]

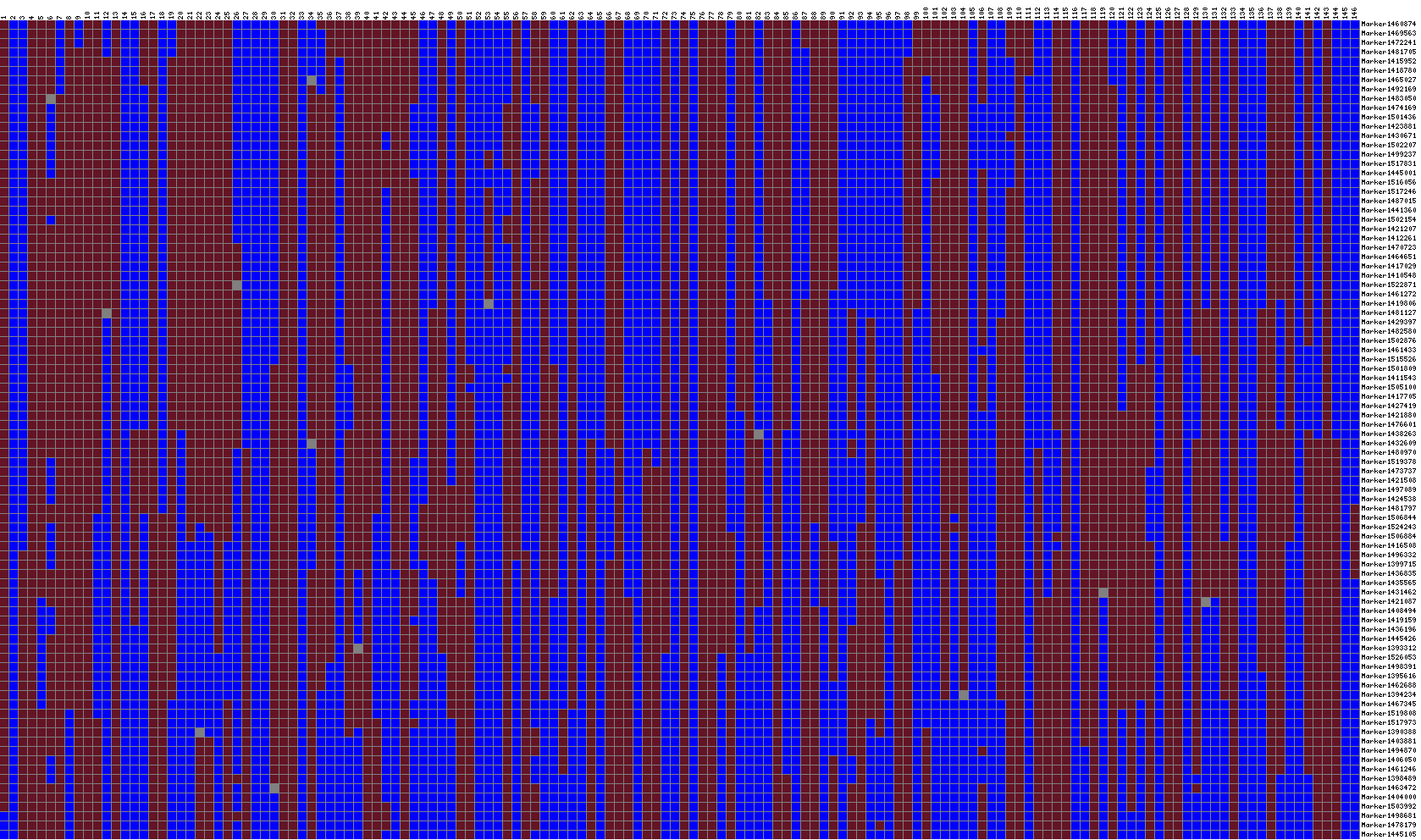

Supplement: Figure S5 — Haplotype map of the genetic map. Blue represents Nannong94-156, red represents Bogao, white means the parent could not be estimated, gray represents deletions. [file FigureS5.ZIP › Chr20.loc.haplo.png]

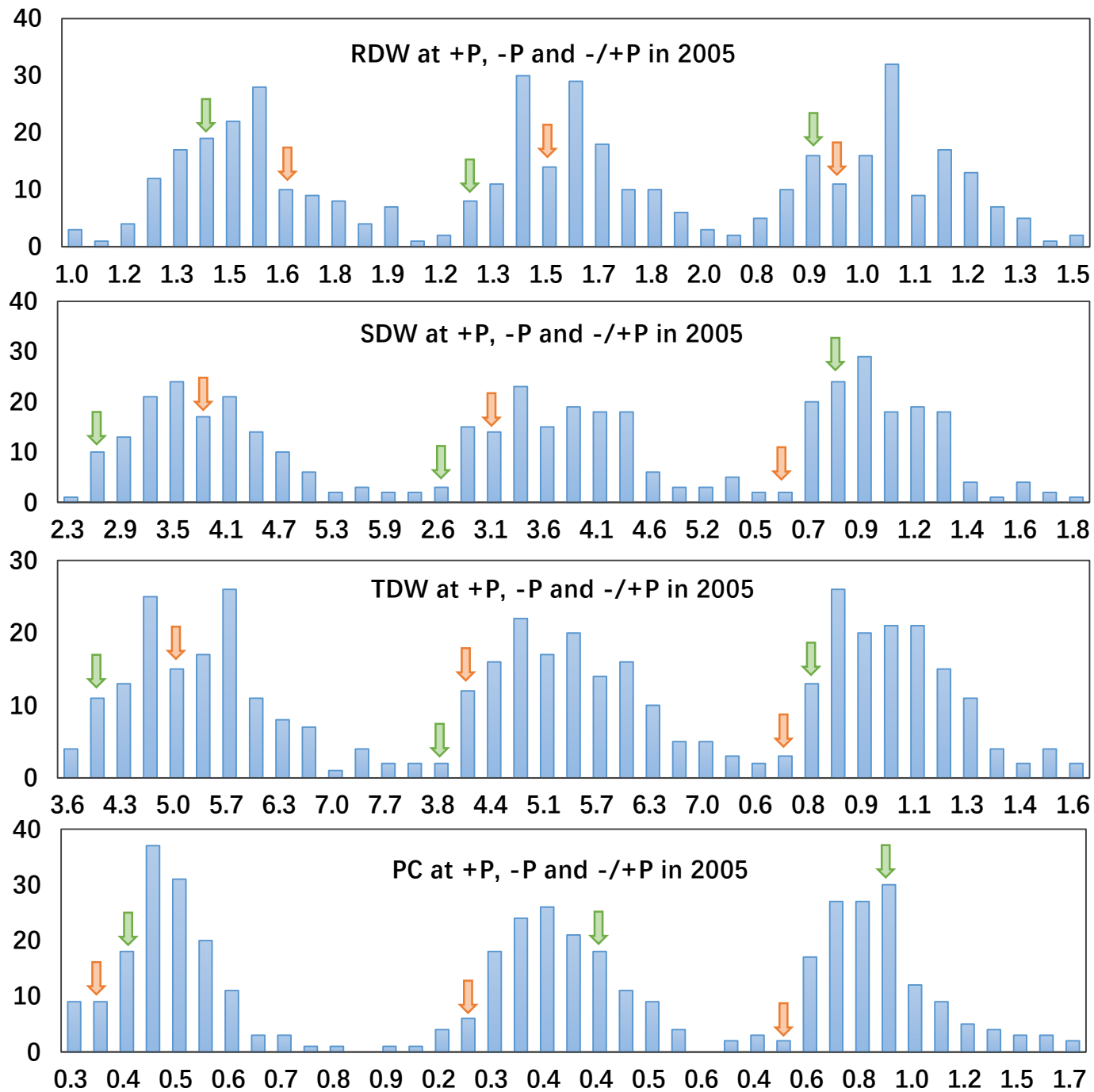

↓ Baogao

↓ Nannong94-156

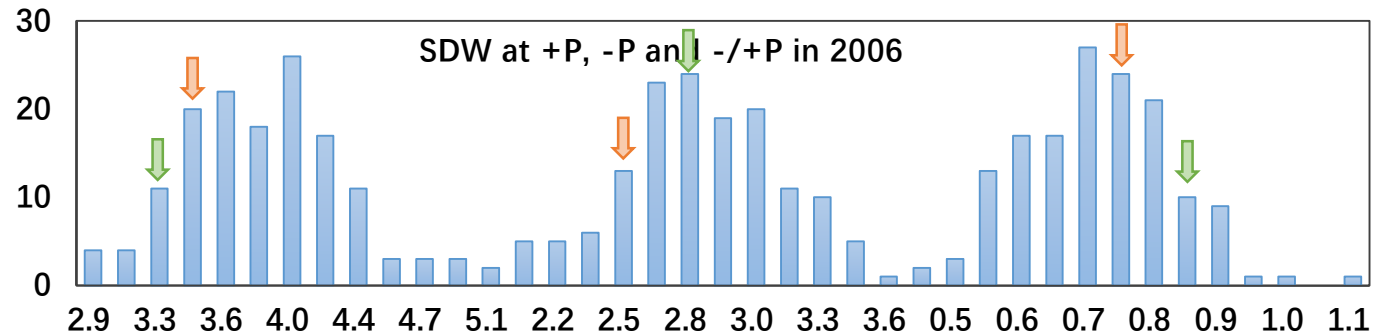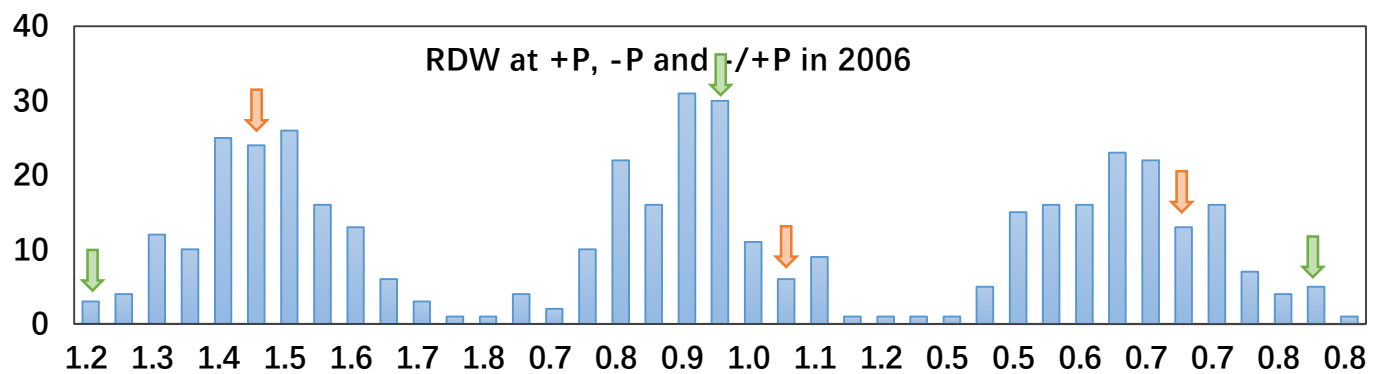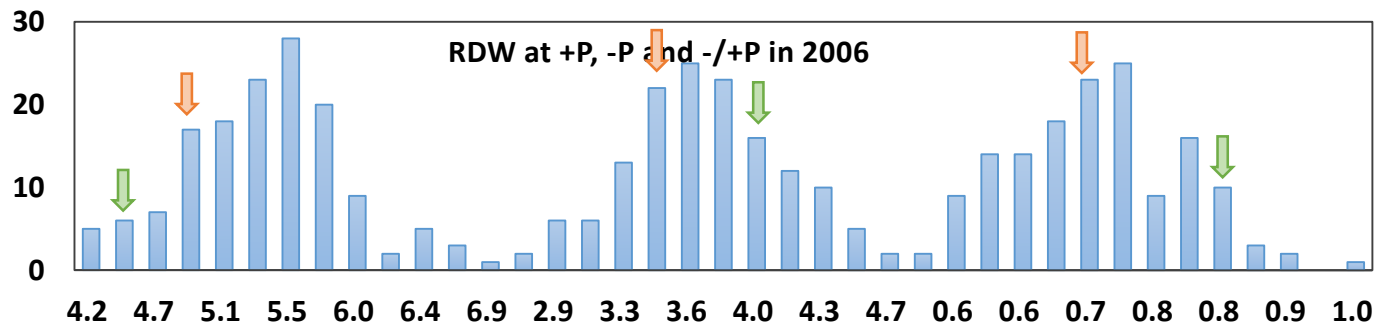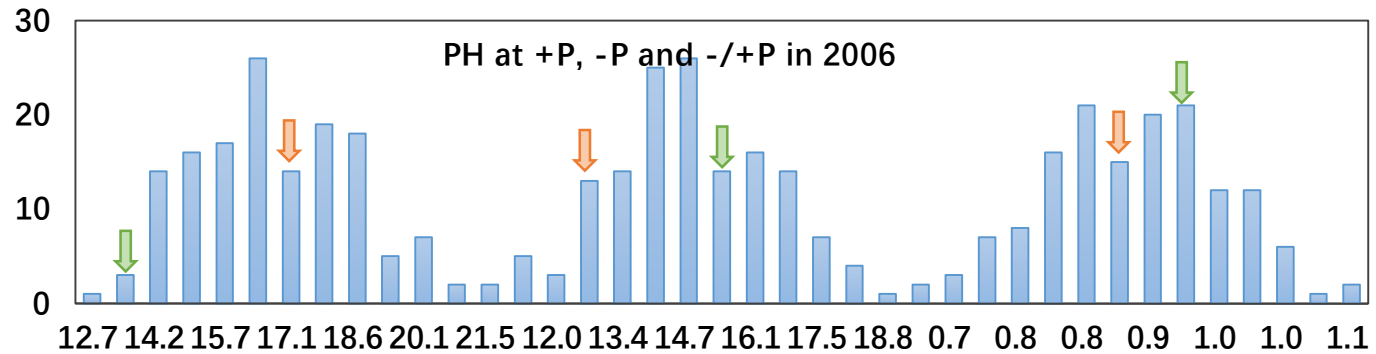

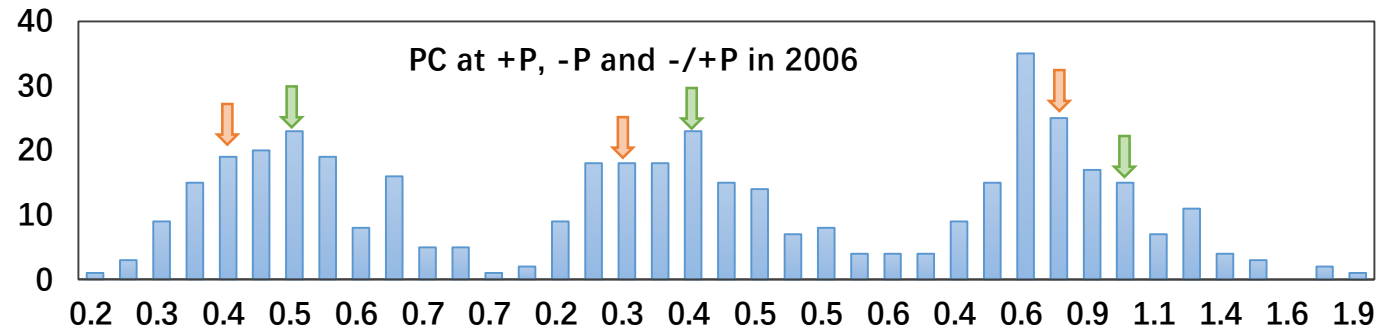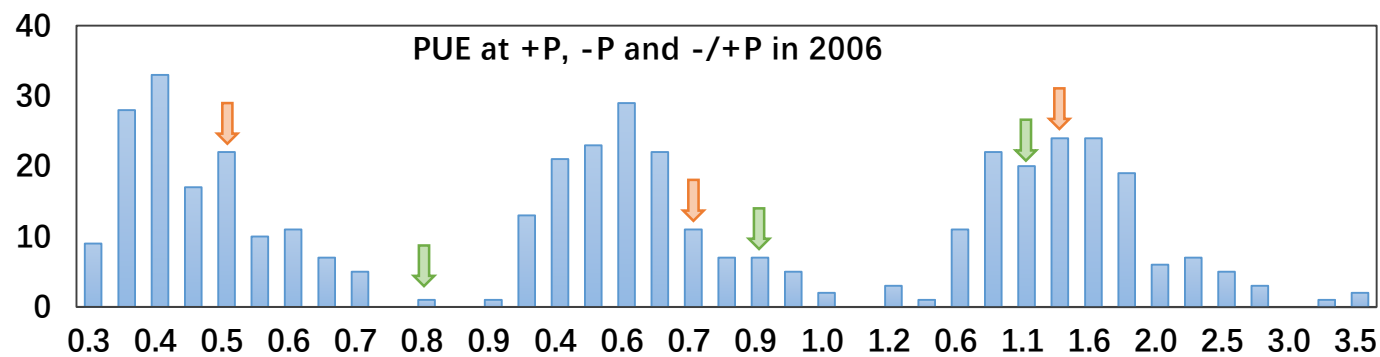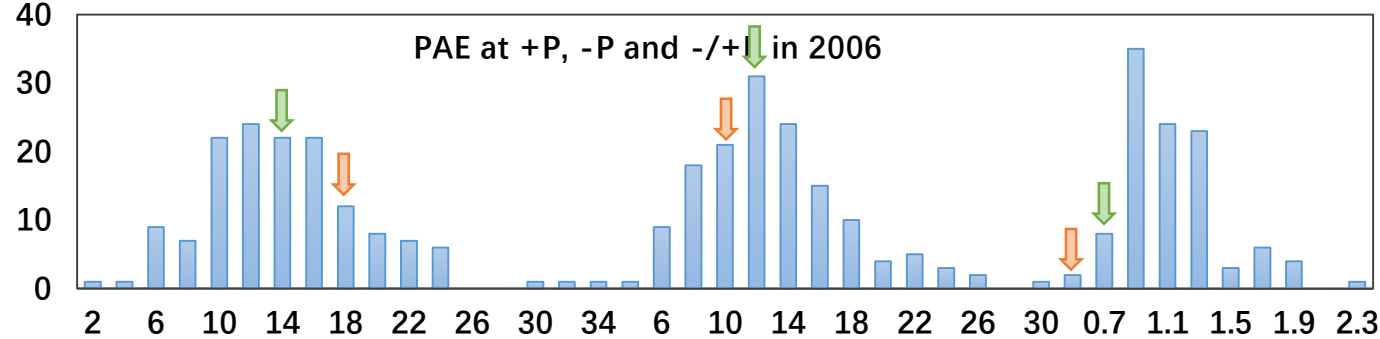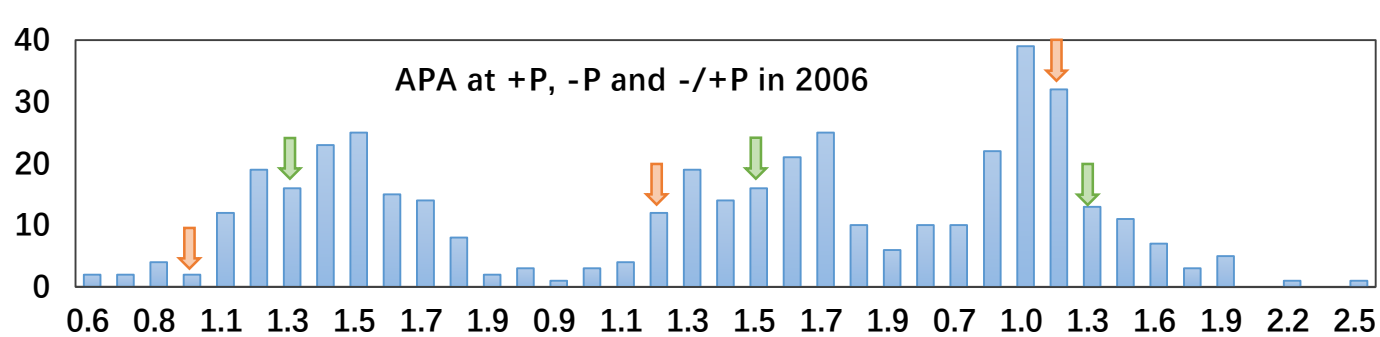

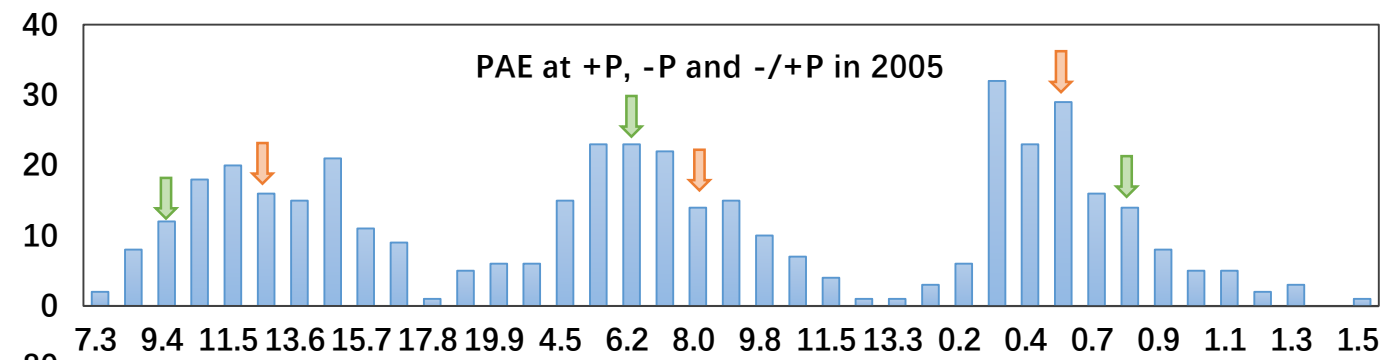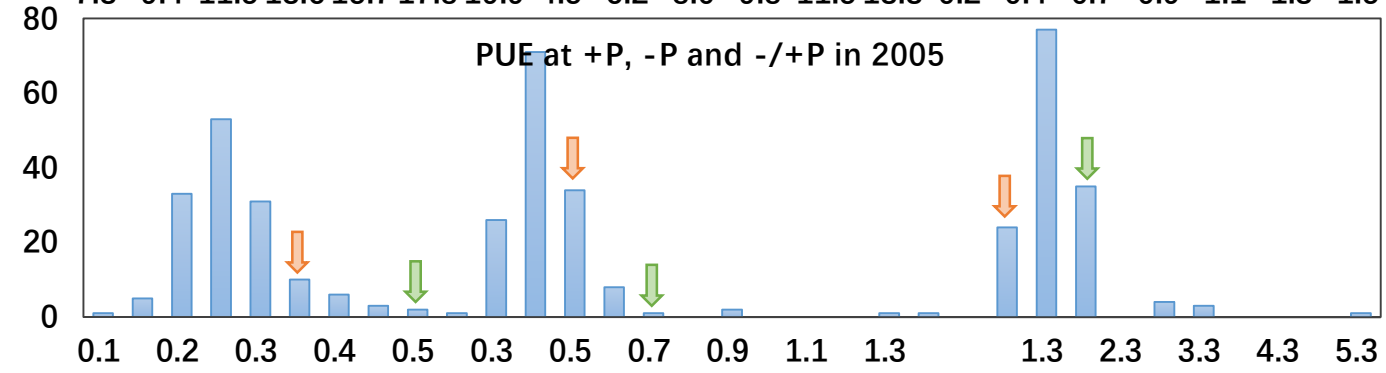

Supplement: Figure S6 — Frequency distribution of P efficiency-related traits at high P (+P), low P (−P) and the ratio of low/high P (−∕+P). The red/green arrows denote the values of the two parents (Bogao and Nannong94-156) on the charts. SDW, shoot dry weight; RDW, root dry weight; TDW, total dry weight; PAE, P acquisition efficiency; PUE, P use efficiency; PH, plant height; APA, acid phosphatase activity; PC, P concentration; +P, high-P; −P, low-P; −∕+P, the ratio of the traits under low P to high P. [file FigureS6.PDF]
